# Understanding Trade-Offs in Continuous Neural Representations for Diffeomorphic Image Registration: A Comparative Study of Implicit Neural Representations and Neural Ordinary Differential Equations

**DOI:** 10.3390/jimaging12080384

**Published:** 2026-08-14

**Authors:** Salvador Rodriguez-Sanz, Carlos Paesa-Lia, Monica Hernandez

**Affiliations:** 1 Aragon Institute of Engineering Research (I3A), University of Zaragoza (UZ), 50018 Zaragoza, Spain; srodsanz@unizar.es (S.R.-S.); cpaesa@unizar.es (C.P.-L.); 2 Computer Science and Systems Engineering Department, University of Zaragoza (UZ), 50018 Zaragoza, Spain

**Keywords:** Large Deformation Diffeomorphic Metric Mapping (LDDMM), diffeomorphic image registration, implicit neural representations (INRs), neural ordinary differential equations (NODEs), neural representations, continuous transformation modeling

## Abstract

Non-rigid image registration is a fundamental problem in medical imaging and a representative example of continuous transformation modeling in image processing. Diffeomorphic registration methods, such as Large Deformation Diffeomorphic Metric Mapping (LDDMM) and its PDE-constrained variants (PDE-LDDMM), provide mathematically grounded formulations with strong geometric guarantees for transformation quality. However, existing approaches face persistent trade-offs between numerical stability, accuracy, and computational efficiency. Recent work has explored implicit neural representations (INRs) and neural ordinary differential equations (NODEs) as flexible neural representations for modeling continuous transformations. Despite their increasing adoption, their practical behavior and limitations in diffeomorphic registration remain insufficiently understood. In this paper, we present a unified formulation of INR- and NODE-based registration methods within LDDMM and PDE-LDDMM, enabling a systematic and controlled comparison across architectures, sampling strategies, and numerical solvers. Our analysis reveals fundamental trade-offs between these approaches. In particular, we show that MLP-based INR formulations introduce significant computational overhead and rely on sampling strategies that can degrade smoothness and lead to the increased occurrence of non-diffeomorphic transformations at higher resolutions. Moreover, these approximations do not fully alleviate the computational cost, with some variants exceeding the costs of expensive classical optimization-based methods. In contrast, NODE-based formulations and downsampling strategies consistently provide transformations with more controlled Jacobian extrema while maintaining competitive computational performance. Among the evaluated methods, the original NODE-LDDMM and NODE-PDE-LDDMM formulations achieve the most favorable trade-offs between registration accuracy, geometric consistency, and computational efficiency. These findings provide clear insights into the design of neural representations for continuous transformation modeling, with practical implications for diffeomorphic registration and computational anatomy applications.

## 1. Introduction

Non-rigid image registration is a fundamental problem in medical imaging and a representative example of continuous transformation modeling in image processing [1]. Diffeomorphic registration methods provide smooth and invertible transformations that preserve the topology of anatomical structures, making them particularly suitable for the analysis of spatial and temporal variability in computational anatomy [2,3,4,5,6,7]. Among these approaches, methods based on the Large Deformation Diffeomorphic Metric Mapping (LDDMM) framework [8,9,10] and its PDE-constrained extensions [11,12,13] offer strong theoretical guarantees for modeling physically consistent transformations. However, despite their solid mathematical foundations, these methods rely on computationally intensive optimization procedures, which can limit their scalability and practical applicability, particularly in high-dimensional settings.

At the same time, learning-based approaches have emerged as an alternative for modeling complex transformations, given the expressive power of neural networks. These methods can provide efficient inference but often introduce trade-offs in terms of generalization and consistency, especially when dealing with large deformations or limited training data. These limitations reveal a key challenge in continuous transformation modeling: achieving a balance between expressiveness, computational efficiency, and geometric consistency.

Implicit neural representations (INRs) have recently emerged as a flexible approach for modeling continuous signals, including images, shapes, and physical processes [14,15]. By representing functions directly through neural networks, INRs enable continuous and differentiable formulations that differ fundamentally from traditional grid-based representations. This has motivated their adoption in non-rigid [16,17,18] and diffeomorphic registration [18,19,20,21], where they offer a flexible alternative for modeling complex transformations.

Despite their increasing use, the practical behavior of INR-based approaches in diffeomorphic registration remains insufficiently understood. In particular, it is unclear how these methods scale with resolution, how their computational cost compares to those of classical and modern formulations, and to what extent they preserve the geometric properties required for diffeomorphic transformations.

To date, INRs have been incorporated into non-rigid registration methods through two qualitatively distinct approaches. In the first one, INRs are used to represent spatial vector fields, such as displacement or velocity functions, by learning a mapping from spatial coordinates to function values [16]. In the second one, neural networks parameterize the right-hand side of a differential equation, giving rise to neural ordinary differential equation (NODE) formulations [18,19,21]. These approaches model transformations as dynamical systems, capturing their evolution through continuous trajectories and enabling integration within PDE-constrained frameworks [21].

While both formulations rely on neural networks to model continuous functions, they differ fundamentally in how transformations are represented: either as spatial vector fields or as dynamical systems. Despite their conceptual differences, these approaches have largely been developed independently, and their relative advantages and limitations in diffeomorphic registration have not been systematically characterized. In this work, we address this gap by providing a unified and systematic analysis of INR- and NODE-based formulations within the LDDMM and PDE-LDDMM frameworks.

The main contributions of this work are summarized as follows:

- We provide a unified formulation of implicit neural representations (INRs) and neural ODEs (NODEs) within the LDDMM and PDE-LDDMM frameworks, enabling a direct and consistent comparison of neural representations for diffeomorphic registration.
- We conduct a systematic analysis of INR- and NODE-based registration methods across a broad range of network architectures (SIRENs and CNNs), spatial sampling strategies (downsize, patch, hybrid, and downsample), and ODE solvers (Scaling and Squaring, Euler, Runge–Kutta, and NODE-based integration schemes).
- We reveal the key limitations of INR-based formulations, showing that commonly used sampling strategies can degrade smoothness and compromise diffeomorphic consistency, leading to the increased occurrence of non-diffeomorphic transformations at higher resolutions.
- We demonstrate that NODE-based formulations and downsampling strategies provide more stable and geometrically consistent transformations, and their combination achieves the most favorable evaluation trade-off across all configurations.
- We provide practical insights into the design of neural representations for continuous transformation modeling, clarifying the trade-offs between expressiveness, computational efficiency, and geometric consistency in signal processing settings.

Our manuscript proceeds as follows. Section 2 provides an overview of the state-of-the-art methods most related to our work. Section 3 summarizes the most important aspects of the LDDMM and PDE-LDDMM methodologies. Section 4 describes the modules of the different variants of LDDMM and PDE-LDDMM based on INRs. Section 5 provides a description of the datasets used for the evaluation of the considered methods, presents the methods selected as baselines, provides the most relevant implementation details, and indicates the parameter values used in this study. Section 6 shows the obtained evaluation results. Finally, Section 7 discusses the most relevant findings of this study, and Section 8 provides the most important conclusions.

## 2. Related Work

In this section, we describe the methods most closely related to our work. Table 1 gathers the most important details of these methods in terms of the transformation parametrization, the implicit neural representation (INR) model, the sampling, and the ordinary differential equation (ODE) solver. These methods are mostly pairwise, learning a different neural network for each image pair.

### 2.1. IDIR: Implicit Neural Representations for Deformable Image Registration

IDIR represents the first approach to non-rigid registration utilizing implicit neural representations [16]. The objective is to find a transformation between the fixed and moving images using a small deformation parametrization, ϕ(x)=x+u(x), where u(x) is modeled through an implicit neural representation $u_{\theta }\left(x\right)$. The neural network architecture is a multilayer perceptron (MLP) with sinusoidal periodic activation functions (SIRENs). Notably, no positional encoding is incorporated into the architecture. Random sampling is used to reduce the computational complexity of the algorithm. The loss function combines normalized cross-correlation (NCC) as the image similarity loss with physics-based regularizers, including hyperelastic and bending energy regularizers, as well as a regularizer for Jacobian control. The authors applied the proposed method to pulmonary CT registration (DIR-LAB 4D CT). It is particularly remarkable that, despite using very sparse random sampling, the model is able to learn a global representation of the displacement fields.

### 2.2. Nature: Exploring the Performance of INRs for Brain Image Registration

In [17], the authors used publicly available IDIR codes to compare the performance of various activation functions in brain image registration tasks. To retain detailed spatial information of the image domain, harmonic encoding was incorporated into the network architecture, addressing a common limitation of MLPs, which tend to lose sensitivity to fine spatial variations and often produce oversmoothed representations of the function of interest [23]. Patch sampling at random locations was employed to reduce the computational complexity of the algorithm. The loss function combined NCC with a regularizer for Jacobian control, and the emphasis was placed on the different activation functions. The authors conducted an extensive hyperparameter influence study, identifying the regularization parameters as the most influential for the performance of the methods. Among the activation functions tested, the registration performance was comparable to that achieved with SIRENs. Experiments were conducted on the Mindboggle101 brain MRI database.

### 2.3. DNVF: Diffeomorphic Image Registration with Neural Velocity Field

DNVF combines stationary diffeomorphic image registration with implicit neural representations [20]. Specifically, the implicit neural representation $v_{\theta }\left(x\right)$ is used to represent the stationary velocity field v(x). The transformation ϕ(x) is computed by solving the transport equation $\frac{d\phi }{dt}=v_{t}\circ \phi_{t}$ with initial condition ϕ(0)=id. In the stationary parametrization, the velocity field $v_{t}\left(x\right)$ remains constant over time v(x), and the Scaling and Squaring (SS) solver [24] is included in the forward and backward passes during training and inference.

Rather than using MLPs, DNVF adopts a fully convolutional network with a UNet-like structure. Image registration is approached with a two-stage cascade, where the initial estimation of $v_{\theta }$ is refined with a small deformation between the warped image and the reference. The loss function combines NCC with a regularizer for Jacobian control and a regularizer for the magnitude of the gradient of the transformation. In order to reduce the GPU memory usage, the implicit representation $v_{\theta }\left(x\right)$ is learned on a downsized domain. The method was evaluated on the OASIS and Mindboggle101 brain MRI datasets. As with IDIR, it is remarkable that the model is able to learn a global representation of the velocity field despite the reduction in the resolution, which should negatively impact the values of the image and the regularization losses.

### 2.4. MIRNF: Medical Image Registration via Neural Fields

MIRNF methods are built on previous IDIR and DNVF proposals to offer a comprehensive family of non-rigid image registration techniques with implicit neural representations [18]. The MIRNF variants are the result of combining the transformation parametrizations with the coordinate sampling strategies. Specifically, these methods use the small deformation and the stationary parametrizations of transformations, along with downsize, patch, and hybrid domain sampling.

The core algorithmic design features are Fourier coordinate encoding, the use of MLPs with SIREN activation functions, and the loss function based on the combination of the NCC with the Jacobian. For the stationary parametrization, the corresponding variant uses NODEs combined with Runge–Kutta in place of Scaling and Squaring. The authors conducted an extensive evaluation study in Mindboggle101 and OASIS, showing the superiority of the stationary method with hybrid sampling with respect to the other variants and selected baselines. Although the authors compared their method with an in-house implementation of NODEO [19], convergence issues prevented a demonstration of the superiority of one approach over the other. The authors identified time complexity as a primary limitation of the best-performing MINRF variants.

### 2.5. NODEO: Neural Ordinary Differential Equation Optimization

NODEO was introduced in [19] as a fresh learning-based approach to tackle diffeomorphic registration problems using neural ordinary differential equations (NODEs), a framework for learning solutions to ODE solvers [25]. In NODEO, the implicit neural representation $v_{\theta }\left(x\right)$ models the stationary velocity field v(x). The NODE formulation enables the model to learn the velocity field as the right-hand-side (rhs) of the LDDMM differential equation $\frac{d\phi }{dt}=v_{t}\circ \phi_{t}$ with initial condition ϕ(0)=id and compute the solution ϕ(1) of the ODE using a Euler solver. Like DNVF, NODEO adopts a fully convolutional network architecture. The CNN also includes a smoothing kernel *K* in its final layer. The loss function combines the localized version of NCC (lNCC), a regularizer for Jacobian control, a regularizer for the magnitude of the gradient of the transformation, and a regularizer for the magnitude of *v*. In this case, the optimization is conducted in the entire image domain, but NODEO optionally downsamples the image domain within the network’s forward pass to reduce memory usage. The method was evaluated on the OASIS dataset.

### 2.6. NODEO-LDDMM and NODEO-PDE-LDDMM

These methods were recently introduced to bridge the gap between LDDMM, PDE-LDDMM, and NODEO [21]. LDDMM provides a foundational framework for diffeomorphic registration by representing transformations as paths in the manifold of diffeomorphisms driven by smooth velocity fields. PDE-LDDMM was proposed in [11] and later enhanced in [12,13,26] to extend the principles of Stokes optical flow to the diffeomorphic setting. This extension allows PDE-LDDMM to model diffeomorphisms with complex properties, including compressibility and incompressibility, mass and intensity preservation, and boundary-preserving non-linear Stokes fluid behavior. In particular, PDE-LDDMM includes inverse consistency constraints, ensuring that transformations remain consistent in both directions. Both families of diffeomorphic registration methods provide mathematically rigorous formulations for diffeomorphic registration.

Combining LDDMM and PDE-LDDMM with NODEs offers the flexibility of deep learning in the computation of the ODE integrals. Both NODEO-LDDMM and NODEO-PDE-LDDMM include the strengths of deep learning in LDDMM, enabling robust optimization with a good balance between accuracy and transformation smoothness in their solutions. The methods were extensively evaluated on the NIREP16 and Learn2Reg OASIS databases.

### 2.7. NePhi: Neural Deformation Fields for Approximately Diffeomorphic Medical Image Registration

NePhi, introduced in [22], redefines the use of INRs for pairwise non-rigid registration by adopting a data-driven deep learning approach. Firstly, a latent code $z=[z_{g},z_{l}\left(x\right)]$, composed of a global and local map component, is extracted from image pairs using a CNN. Then, the forward and inverse transformations are modeled through INRs. During training, the three networks (the CNN and the two IRNs) are jointly optimized on a dataset of image pairs, consistent with the principles of conventional deep learning methodologies. However, unlike traditional INR-based methods, the INRs in NePhi are conditioned on latent codes inferred by the CNN, rather than being learned independently for each pair. This distinction places NePhi outside the conventional family of INRs, as its representations are not strictly pairwise and are inherently dependent on a global model. In addition, NePhi needs to be combined with previous pre-alignment and subsequent instance optimization to achieve competitive results, reflecting its reliance on alternative deep learning and pairwise techniques. The method was evaluated on a large variety of datasets, including DRIVE retina images, OAI knee MRI, HCP brain MRI, and COPDGene and DirLab pulmonary CT.

### 2.8. Key Observations from Related Work

The analysis of the related work leads to the following observations:

(1) The use of MLP architectures introduces significant computational complexity. While spatial encoding effectively enriches the input representation, it also adds to the dimensionality of the problem, increasing the overall complexity. To address memory constraints, many methods rely on downsizing strategies during training. Interestingly, these methods are often described as efficient in the literature, despite their computational demands in their forms without sampling strategies. However, it is worth noting that, even with reduced data sizes during training, these models have demonstrated an impressive capacity to learn accurate function estimations.
(2) The evaluation of existing methods spans anatomies of varied nature, such as pulmonary and brain registration problems. This lack of homogeneity in the evaluation datasets poses challenges in directly comparing the performance of different methods and identifying which method outperforms the others for specific tasks.
(3) The selection of hyperparameters remains a complex process, involving numerous parameters and often requiring exploration across a broad range of values.
(4) Although implicit neural representations (INRs) and neural ordinary differential equations (NODEs) share conceptual similarities, their relative advantages, limitations, and trade-offs in diffeomorphic registration remain insufficiently understood. Existing comparisons are limited in scope, making it difficult to draw clear conclusions about their behavior across different modeling and computational settings.

## 3. Large Deformation Diffeomorphic Metric Mapping (LDDMM)

In this section, we briefly describe the foundations of LDDMM and PDE-constrained variants. The input of the image registration problem is represented with $I_{0}$ (moving) and $I_{1}$ (fixed) images. The moving and fixed images are also referred to as source and target images in the extensive LDDMM literature. In the continuous domain, the images are considered square-integrable functions $I_{i}:\Omega \to R$, where Ω is a rectangular domain in $R^{d}$, and d=2 or 3. Diff(Ω) represents the Riemannian manifold of smooth diffeomorphisms on Ω, where *V* is the tangent space of the Riemannian structure at the identity diffeomorphism, id. *V* is a vector space of smooth vector fields on Ω. With the composition of transformations and the Lie group operator, Diff(Ω) has a Lie group structure, and *V* is the Lie algebra.

### 3.1. Original Large Deformation Diffeomorphic Metric Mapping

Large Deformation Diffeomorphic Metric Mapping (LDDMM) was proposed by Beg et al. in 2005 [8]. LDDMM is approached with a variational formulation from the minimization of the energy functional

(1) $$E\left(v\right)=E_{reg}\left(v\right)+\frac{1}{\sigma^{2}}E_{img}\left(I_{0}\circ \varphi^{-1},I_{1}\right),$$

where $\varphi^{-1}:\Omega \to R^{d}$ is the diffeomorphism that warps the moving image into the fixed image. The total energy *E* is decomposed into the regularization $E_{reg}$ and the image similarity metric $E_{img}$. The parameter $\frac{1}{\sigma^{2}}$ weights the contributions of the regularization and image similarity to the total energy.

LDDMM assumes that transformations belong to the Riemannian manifold Diff(Ω). The Riemannian metric of Diff(Ω) is defined from the scalar product in *V*,

(2) $${\langle v,w\rangle }_{V}={\langle Lv,Lw\rangle }_{L^{2}}={\langle L^{†}Lv,w\rangle }_{L^{2}}=\int_{\Omega }\langle L^{†}Lv\left(x\right),w\left(x\right)\rangle d\Omega ,$$

where $L={\left(Id-\alpha \Delta \right)}^{s}$, is the invertible self-adjoint differential operator defining the differential structure of Diff(Ω).

Instead of defining the energy on $\varphi^{-1}$ directly, the variational problem is parametrized with $v_{t}\in L^{2}\left(\left[0,1\right],V\right)$. The transport equation

(3) $$\frac{d\phi_{t}}{dt}=v_{t}\circ \phi_{t}$$

corresponds to the Riemannian exponential map between the elements in *V* and the corresponding elements in the manifold of diffeomorphisms Diff(Ω). Thus, $v_{t}$ is a time-varying velocity field that represents the tangent vectors of the path of diffeomorphisms $\phi_{t}$, beginning in the identity $\phi_{0}=id$ and ending in $\phi_{1}=\varphi$ and yielding the minimum energy for the LDDMM problem. Using Beg et al.’s notation trick $\phi_{s,t}=\phi_{t}\circ \phi_{s}^{-1}$, the transport equation can be written as

(4) $$\frac{d\phi_{0,t}}{dt}=v_{t}\circ \phi_{0,t}.$$

In LDDMM, the regularization energy is defined from

(5) $$E_{reg}\left(v_{t}\right)=\int_{0}^{1}{∥v_{t}∥}_{V}^{2}dt,$$

where ${∥\cdot ∥}_{V}^{2}={\langle \cdot ,\cdot \rangle }_{V}$. Thus, the length of the path of diffeomorphisms $\phi_{t}$ is given by $E_{reg}\left(v_{t}\right)$. If we assume that the intensities of the images exactly match at convergence, $E_{img}\left(I_{0}\circ \varphi^{-1},I_{1}\right)=0$. Then, the solution $v_{t}$ yields a flow of diffeomorphisms $\phi_{t}$, which is a geodesic in Diff(Ω) with the Riemannian metric. In practice, the matching is not exact, and the solutions depart slightly from belonging to geodesic paths.

The image similarity energy is usually defined from the sum of squared differences (SSD),

(6) $$E_{img}\left(I_{0}\circ \varphi^{-1},I_{1}\right)={∥I_{0}\circ \varphi^{-1}-I_{1}∥}_{L^{2}}^{2},$$

although we can replace SSD with the most commonly used image similarity metrics in medical image registration problems: normalized cross-correlation (NCC), its local version (lNCC), mutual information (MI), and normalized gradient fields (NGFs) [27,28,29]. Both NCC and lNCC have been shown to surpass the performance of SSD even in monomodal problems [29].

### 3.2. PDE-Constrained Large Deformation Diffeomorphic Metric Mapping

The original LDDMM formulation was extended to PDE-LDDMM with a constrained variational problem using an optimal control approach [11]. PDE-LDDMM constitutes a formulation that is analytically but not numerically equivalent to LDDMM. The constraints are included in the shape of differential equations, which yield physically meaningful solutions, such as incompressible transformations or transformations belonging to geodesics under the EPDiff equation [12,26,30].

The fundamental constraints in PDE-LDDMM are derived from the inverse consistency identity [8], yielding the deformation state equation

(7) $$\partial_{t}\phi_{t,0}+D\phi_{t,0}\cdot v_{t}=0\left(\mathrm{forward}\right)$$

and its inverse

(8) $$-\partial_{t}\phi_{0,t}-D\phi_{0,t}\cdot v_{t}=0\left(\mathrm{backward}\right)$$

with initial conditions $\phi_{0,0}=id$ and $\phi_{0,1}=id$, respectively. Three different PDE-LDDMM variants have been proposed in the literature. We focus on the two PDE-LDDMM variants proposed in [13], which have been shown to surpass the original PDE-LDDMM proposal [11,12].

The first one, PDE-LDDMM_st eq_, uses the temporal solution from the deformation state equation (Equation (7)) applied to the initial state variable $I_{0}$, yielding $m_{t}=I_{0}\circ \phi_{t,0}$. In addition, the temporal solution of the backward deformation state equation is applied to the initial adjoint variable $\lambda \left(1\right)=\frac{2}{\sigma^{2}}\left(I_{1}-m\left(1\right)\right)$, yielding $\lambda_{t}=J_{t}\lambda_{1}\circ \phi_{0,t}$, where $J_{t}=\left|D\phi_{0,t}\right|$ is computed from the inverse deformation equation (Equation (8)). The gradient needed for gradient descent optimization results in

(9) $$\nabla_{v}E\left(v_{t}\right)=2v_{t}+{\left(L^{†}L\right)}^{-1}\left(\lambda_{t}\nabla m_{t}\right).$$

The expression for $v_{t}$ can be computed from the gradient update equation or using the fixed-point iteration based on the gradient $\nabla_{v}E\left(v\right)$

(10) $$v_{t}\to -\frac{1}{2}{\left(L^{†}L\right)}^{-1}\left(\lambda_{t}\nabla m_{t}\right)$$

previously used in the LDDMM literature [31]. During optimization, $\nabla_{v}E\left(v\right)\to 0$. Consequently, the right-hand side of Equation (9) can be considered a rough approximation of *v* at the beginning of the optimization process that increases its precision iteratively.

The second one, PDE-LDDMM_def eq_, uses the deformation state equation directly in the constrained optimization problem. The differentiation of the augmented Lagrangian with respect to the state variable ϕ and its adjoint variable ρ yields the optimality conditions

(11) $$\begin{array}{cc} \partial_{t}\phi_{t,0}+D\phi_{t,0}\cdot v_{t} & =0,\phi_{0,0}=id\\ -\partial_{t}\rho_{t}-\nabla \cdot \left(\rho_{t}\cdot v_{t}\right) & =0,\rho (1)=\lambda (1)\cdot \nabla m(1). \end{array}$$

The gradient for gradient descent optimization is, in this case,

(12) $$\nabla_{v}E\left(v_{t}\right)=2v_{t}+{\left(L^{†}L\right)}^{-1}\left(D\phi \cdot \rho \right).$$

Analogously, the expression $v_{t}$ can be computed from the gradient update

(13) $$v_{t}\to -\frac{1}{2}{\left(L^{†}L\right)}^{-1}\left(D\phi_{t}\cdot \rho_{t}\right).$$

The time-varying parametrization of the velocity fields of the LDDMM problem has been replaced with stationary or steady velocity fields [9,10]. Due to its computational efficiency, the stationary parametrization has been extensively used as a fundamental building block in modern deep learning approaches [32,33,34,35].

## 4. Implicit Neural Representations for LDDMM and PDE-LDDMM

This section introduces the different variants that emerge from the integration of INRs into LDDMM and PDE-LDDMM. The following subsections present the problem formulation, detail the particularities of the implicit representations, and describe the different ODE solvers. Finally, we end with the classification of the proposed variants based on the LDDMM formulation, INR model, sampling strategy, and ODE solver.

### 4.1. Problem Formulation

The proposed methods are based on mathematical foundations similar to those in LDDMM and PDE-LDDMM, as described in Section 3. The main differences lie in the modeling of certain variables, where INRs are employed to learn their neural representations. Learning occurs during the optimization process for solving the minimization problem for each image pair using stochastic gradient descent on different domain samplings.

We denote by θ the parameters of the networks defined for the INR modeling. The loss function combines the terms of the LDDMM variational formulation with regularization terms, preventing the model from inferring solutions that develop foldings or aggressive contractions or expansions. Regularization is essential, as deep learning approaches are prone to minimizing the image similarity loss at the expense of the transformation’s quality, leading to undesirable artifacts. Thus,

(14) $$\begin{array}{cc} L\left(I_{0},I_{1},\theta \right)=\sigma_{reg}L_{reg}\left(v\right)+\sigma_{img}L_{img}\left(I_{0}\circ \varphi^{-1},I_{1}\right) & +\\ & \sigma_{Jdet}L_{Jdet}\left(\varphi^{-1}\right)+\sigma_{grad}L_{grad}\left(\varphi^{-1}\right), \end{array}$$

where $L_{reg}$ is equivalent to $E_{reg}$, and $L_{img}$ is selected as the local normalized cross-correlation metric (lNCC),

(15) $$lNCC\left(I_{0},I_{1}\right)=\int_{\Omega }\frac{\sum_{x\in W}\left(I_{0}\left(x\right)-{\bar{I}}_{0}\right)\left(I_{1}\left(x\right)-{\bar{I}}_{1}\right)}{\sqrt{\sum_{x\in W}{\left(I_{0}\left(x\right)-{\bar{I}}_{0}\right)}^{2}}\sqrt{\sum_{x\in W}{\left(I_{1}\left(x\right)-{\bar{I}}_{1}\right)}^{2}}}d\Omega .$$

In the expression of lNCC, *W* is a local window around each pixel in Ω, and $\bar{I}$ indicates the mean of image *I* inside that window. The losses $L_{Jdet}\left(\varphi^{-1}\right)$ and $L_{grad}\left(\varphi^{-1}\right)$ are defined from

(16) $$\begin{array}{ccc} L_{Jdet}\left(\varphi^{-1}\right) & = & \int_{\Omega }{\left(\max\left(0,-J_{\varphi^{-1}}\left(x\right)+\epsilon \right)\right)}^{2}d\Omega ,\mathrm{and} \end{array}$$

(17) $$\begin{array}{ccc} L_{grad}\left(\varphi^{-1}\right) & = & ∥\nabla \varphi^{-1}{∥}_{L^{2}}^{2}. \end{array}$$

These losses have been previously proposed in different registration methods like VoxelMorph [32], SyMNet [33], NODEO [19], MIRNF [18], or LDDMM-NODEO [21], showing their utility in controlling the quality of transformations [36]. In particular, the threshold value ϵ in $L_{Jdet}$ can be empirically seen as an important regularization hyperparameter, preventing the model from computing solutions with aggressive contractions and foldings. The hyperparameters σ balance the contributions of the different losses to the total loss.

In this work, we depart from the INRs defined in the small deformation paradigm, where the implicit representation is associated with the displacement field of the transformation. Thus, $\varphi^{-1}$ is parametrized by means of the stationary velocity field *v*. In LDDMM, the INR represents a neural approximation of *v* or a neural approximation of the right-hand side of the transport equation (Equation (3)). On the other hand, in the two variants of PDE-LDDMM, the INRs represent the right-hand sides of the deformation state equations and their adjoints (Equations (7), (8), and (11)). This backbone, shared across LDDMM and PDE-LDDMM with INRs, underpins the variants proposed in this work. The following sections detail the specific architectures, sampling strategies, and solvers leading to each one.

### 4.2. Implicit Neural Representations

Implicit neural representations (INRs) are coordinate-based neural networks that model continuous mappings from the map domain to its range. These representations offer a highly flexible framework for modeling various types of variables in the different non-rigid image registration formulations. By encoding functions implicitly as neural networks, INRs enable the representation of complex transformations in a compact and continuous manner. A key advantage of INRs is their inherent analytic differentiability, which facilitates the application of modern optimization techniques founded on automatic differentiation within deep learning frameworks.

In this work, we consider two different implicit neural representations. The first one is based on a multilayer perceptron architecture (MLP), while the second one is based on a convolutional neural network (CNN).

#### 4.2.1. Multilayer Perceptrons

The architecture of an implicit neural representation (INR) is typically a multilayer perceptron (MLP) with input and output dimensions equal to *d*—or d×T in the case of modeling time-varying functions. To enhance the optimization and expressive power of these models, several design choices are commonly employed, including coordinate encoding, sinusoidal activations, and coordinate sampling.

**Coordinate encoding.** Standard MLPs face significant challenges when learning mappings with high-frequency components or fine-grained details. This limitation, known as spectral bias, reflects the tendency of neural networks to prioritize learning low-frequency components over high-frequency variations. As a result, the spatial coherence and resolution are often compromised in problems that require detailed spatial representations.

To address these issues, the input domain Ω is transformed into a higher-dimensional space using a coordinate encoding technique [14]. This transformation enriches the input with periodic features that preserve positional consistency, ensuring spatial coherence. Furthermore, the encoding explicitly introduces high-frequency information, which mitigates the spectral bias of MLPs and improves their ability to represent complex structures and fine-grained details, thereby enabling accurate spatial modeling.

In this work, the embedding function is defined as

(18) $$\epsilon \left(x\right)=[e_{1}\left(x\right),e_{2}\left(x\right),\ldots ,e_{n}\left(x\right),x],$$

where Fourier encodings map the input coordinates to a higher-dimensional embedding space [14] using the family of functions $e_{i}:R^{d}\to R^{2d}$,

(19) $$e_{i}\left(x\right)=\left[cos\left(2\pi \omega_{i}x\right),sin\left(2\pi \omega_{i}x\right)\right].$$

The parameters $w_{i}\in R^{d}$ are sampled independently and identically from a Gaussian distribution. The use of Fourier encoding is crucial for achieving high accuracy in spatial tasks such as vector field modeling, where precise spatial relationships are essential.

**Sinusoidal activations.** Sinusoidal activations play a crucial role in enhancing the expressiveness of the MLPs used in INRs, called SIRENs. Unlike traditional activation functions such as ReLU or tanh, sinusoidal activations enable the network to directly model oscillatory patterns, making them particularly effective for representing high-frequency components in data. A key property of SIRENs is their exponentially large frequency support, which allows the network to efficiently capture a broad range of frequencies, including both low-frequency components and intricate high-frequency details. This property is a direct result of the sinusoidal activation function’s ability to propagate high-frequency information across layers when combined with proper parameter initialization. Sinusoidal activations contribute, together with Fourier encoding, to mitigating spectral bias. In addition, the periodic nature of sinusoidal functions naturally aligns with tasks involving wave-like or periodic phenomena, yielding better INRs for spatial and temporal data.

**Domain sampling.** A key limitation of MLP-based INRs is their high computational complexity, particularly in terms of memory consumption. However, this family of methods has shown extraordinary performance when trained in reduced domains. Various strategies for domain sampling have been proposed, including random sampling [16,17], downsizing [18], patch sampling [17,18], and hybrid sampling [18], which combines downsizing with patch sampling.

In this work, we focus on four domain sampling approaches founded on downsizing, patch selection, hybrid sampling, and downsampling/upsampling. In the first one, we use downsizing similarly to [18]. Let us define the downsizing granularity using a parameter *s*, which specifies the number of pixels skipped between consecutively selected ones. During training, the model learns the parameters across *s*-spaced domains, allowing it to approximate the target function effectively even in a reduced domain. For inference, the INR can reconstruct the function over the entire domain by sequentially processing consecutive pixels in small chunks that fit into memory. Although downsizing leads to some loss of accuracy during the computation of loss functions and gradients during both forward and backward propagation, the optimization still converges to a learned representation that generalizes effectively across the entire image domain, according to the loss function.

In the second one, we use a patch-based sampling strategy similarly to [17]. Let us define the patch size using a parameter *p*, which specifies the size of the patches in each dimension. Equal-size patches are used merely for the sake of simplicity. During training, the model learns the parameters across the *p*-sized domains. In this case, the computation of the loss functions and gradients in forward and backward propagation is conducted in a reduced computational domain of Ω. In this case, no loss of accuracy occurs during the computation of the loss functions and gradients at the patch level.

The third approach combines downsizing with patch-based sampling, as proposed in [18]. In this case, the learned representation from the downsizing domain sampling strategy is composed with the learned representation from the patch-based sampling strategy. During training, the model first learns the model representation with the loss of accuracy inherent to the downsizing approach. Then, patch-based sampling allows for refining the learned function locally with the accurate computation of the loss functions and gradients at the patch level. This hierarchical approach is intended for a balance between accuracy and computational efficiency.

The fourth domain sampling strategy incorporates downsampling and upsampling of the INR input as part of the network layers, similarly to [19,21]. During training, the model optimizes its parameters in the original domain, and memory reduction is achieved through efficient operations within the network’s computational pipeline. For inference, the INR operates seamlessly over the entire domain without requiring additional processing. This approach ensures no decrease in accuracy in the computation of the loss functions or the gradients during training.

Downsize, patch, and hybrid sampling strategies yield domain size-independent models, while the downsampling strategy does not. However, the downsampling strategy allows the use of globally accurate loss functions, while, for the patch strategy, the loss functions are only locally accurate. Strategies involving downsizing use less accurate loss functions in the optimization of the model.

#### 4.2.2. Convolutional Neural Networks

INRs can also be modeled using convolutional neural networks (CNNs), where the spatial coordinate system serves as the input. In this case, the model inherently incorporates the domain shape, eliminating the need for coordinate encoding. Additionally, these models do not require the replacement of ReLU activations with sinusoidal ones. Unlike MLPs, domain downsizing and patches cannot be consistently applied between models trained in the reduced domain and those tested in the original domain. In contrast, the downsampling strategy, which incorporates subsampling and upsampling of the INR input as part of the network layers, can achieve significant dimensionality reductions without compromising accuracy. Optionally, the LDDMM operator ${\left(L^{†}L\right)}^{-1}$ can be added as the final layer to enforce the additional regularization of the output.

### 4.3. Ordinary Differential Equation (ODE) Solvers

#### 4.3.1. Traditional ODE Solvers

The ODE involved in LDDMM is the transport equation given in Equation (3), assuming that *v* is a stationary velocity field (i.e., independent of time). This equation describes the evolution of the diffeomorphic path $\phi_{t,0}$ along the stationary flow *v*. One widely used method for solving the stationary transport equation is Scaling and Squaring (SS) [24], which mimics the computation of the exponential map in matrix Lie groups by recursively scaling the flow and squaring intermediate transformations (i.e., composing intermediate transformations with themselves). This approach is computationally efficient and well suited for the integration of stationary velocity fields.

Alternatively, numerical integration methods such as Euler or Runge–Kutta solvers can be employed to approximate the solution. In this case, on the right-hand-side of the transport equation, $v\circ \phi_{t,0}$ depends explicitly on time, and additional considerations are required. Specifically, the input space of the MLP representing the flow must be extended to include the temporal dimension, resulting in a 3D+t representation. This extension enables the network to model time-dependent dynamics explicitly, providing a more general formulation of the transport equation that can capture the temporal variations of the right-hand side and yield solutions for this scenario. We neglect the use of 3D+t CNNs representations as this is beyond the scope of our work.

The ODEs involved in PDE-LDDMM include the forward and backward deformation state equations, as well as the adjoint equation on the variable ρ, given in Equations (7), (8) and (11). In this case, Scaling and Squaring is not a suitable solver, and one must rely on Euler or Runge–Kutta solvers. The same considerations hold regarding the 3D+t dimensionality of the MLPs.

#### 4.3.2. Neural ODEs

Neural ordinary differential equations (NODEs) represent a learning-based approach to solving ODEs, first proposed in [25]. This method is inspired by the analogy between the Euler method for solving ODEs and the architecture of residual neural networks (ResNets). Instead of viewing the layers of a ResNet as discrete transformations, NODEs generalize this idea by treating the depth of the network as a continuous dimension, effectively replacing the discrete layers with a parameterized continuous function.

In NODEs, the model represents the right-hand side (rhs) of the differential equation, which describes the evolution of the system being modeled. During training, the network learns this rhs function, enabling it to encode the dynamics of the data. The solution to the differential equation is computed using numerical ODE solvers, such as Euler or Runge–Kutta methods, which integrate the learned rhs over time to generate the desired outputs.

One notable limitation of NODEs is the computational complexity of backpropagation through the ODE solver, as storing intermediate states during integration can be memory-intensive. This challenge is addressed by the adjoint sensitivity method, which efficiently computes gradients by solving an adjoint ODE backward in time, reducing the memory requirements.

In this work, NODEs can replace traditional ODE solvers. This involves modeling the rhs of the equations in LDDMM and PDE-LDDMM formulations with either MLPs or CNNs and using the NODE to solve the ODEs, instead of traditional solvers.

### 4.4. Considered Variants

Table 2 gathers the different LDDMM, PDE-LDDMM st. eq., and PDE-LDDMM def. eq. algorithms proposed in this work. Each variant results from combining the LDDMM or PDE-LDDMM formulations with the INRs, domain sampling strategies, and used ODE solvers described above. The table also indicates whether a given variant resembles a method previously proposed in the literature.

For instance, LDDMM combined with a SIREN for $v_{\theta }$, downsize sampling, and Scaling and Squaring is similar to DNVF. The most remarkable distinction lies in the INR model since DNVF uses a CNN. Similarly, LDDMM combined with a SIREN for $v_{\theta }$, downsize sampling, and a NODE with Runge–Kutta (RK) is comparable to one of the methods in MIRNF, with the key difference being the order of the RK scheme. Lastly, the variants closest to NODEO-LDDMM and NODEO-PDE-LDDMM, when combined with a SIREN, differ in the INR used. For completeness, the table also includes NODEO-LDDMM and NODEO-PDE-LDDMM as originally proposed in [21].

It should be noted that the methods using downsize and patch-based sampling cannot include the operator ${\left(L^{†}L\right)}^{-1}$ due to consistency problems between models during training in the reduced domain and testing in the original domain.

The backbone and the general algorithms for INR-LDDMM, INR-LDDMM st. eq., and INR-LDDMM def. eq. are detailed in Appendix A.

## 5. Datasets, Benchmark Methods, Implementation Details, and Parameters

### 5.1. Datasets

The methods in this study are evaluated using two different datasets extensively used for the evaluation of non-rigid registration methods.

#### 5.1.1. NIREP

The Non-Rigid Image Registration Evaluation Project (NIREP) was specifically devised in 2006 for the evaluation of non-rigid registration [37]. This dataset consists of 16 T1 magnetic resonance imaging (MRI) images of young normal individuals from the Human Neuroanatomy and Neuroimaging Laboratory, University of Iowa. The images are provided without the skull and distributed with the manual segmentation of 32 gray matter regions at the frontal, parietal, temporal, and occipital lobes. The geometry of the manual segmentations provides a specially challenging framework for diffeomorphic registration. The image dimensions are 180×212×180 with a voxel size of 1.0×1.0×1.0 mm. The registration pairs include the first image as the source and the remaining 15 images as the target images.

#### 5.1.2. OASIS Learn2Reg22

OASIS-L2R22 is a small-sample dataset composed of 416 3D T1 MRI scans from the Open-Access Series of Imaging Studies, OASIS (https://www.oasis-brains.org/, accessed on 6 July 2022). The dataset was proposed in the Learn2Reg challenge with the intention of assessing the performance of non-rigid registration methods in the alignment of small structures of variable shape and size from monomodal MRI [38]. A total of 35 segmentations are provided from FreeSurfer and SAMSEG from the neurite package (https://surfer.nmr.mgh.harvard.edu/fswiki/Samseg, accessed on 1 March 2024). The registration pairs were given by the challenge organizers for reproducibility. The image dimensions are 160 × 224 × 192. The test set is composed of 39 image pairs, but the segmentations are not publicly available. For this work, we segmented the test images using SAMSEG. We observed that this version of SAMSEG provided many more labels than the Learn2Reg segmentations, and there was a mismatch in the labels assigned to corresponding structures. We manually combined and reassigned the labels to be mostly in correspondence with the Learn2Reg segmentations.

We validated the differences in our segmentations in the validation set with respect to the official Learn2Reg segmentations. The overall mean DSC was 0.90, while the median DSC was 0.92. The maximum and minimum DSC values were 0.97 and 0.59, respectively. The agreement was generally high across anatomical structures, with most DSC values exceeding 0.90. Lower agreement was primarily observed for the inferior lateral ventricle, vessel, and choroid plexus, which are relatively small anatomical structures known to be challenging for automated segmentation methods. Visual inspection indicated that most discrepancies were localized around anatomical boundaries rather than reflecting substantial differences in anatomical delineation. These discrepancies are likely related to differences in SAMSEG software versions.

Overall, the observed differences are unlikely to substantially affect the relative ranking of the evaluated registration methods.

### 5.2. Methods Selected as Benchmark

In this work, we selected the traditional LDDMM and PDE-LDDMM methods closest to our work as benchmark methods. We also selected the most popular unsupervised deep learning approaches with available source code and models trained in the T1w MRI registration problem. Given the prevalence of the stationary parametrization of diffeomorphisms in unsupervised deep learning approaches, we focused our study on the evaluation of stationary LDDMM and PDE-LDDMM. Thus, we selected as benchmarks the best-performing stationary versions of LDDMM and PDE-LDDMM in [29] (NCC or lNCC image similarity and Gauss–Newton–Krylov optimization) and ANTS (lNCC image similarity) [39]. From the deep learning methods, we selected VoxelMorph [40], SyMNet [33], LapIRN [34], and the B-Spline version of TransMorph [35], since these methods provide nearly diffeomorphic transformations. We also selected NODEO-CVPR22 with the default parameters as a benchmark [19]. Finally, we included the results from our previous work for NODEO-LDDMM and NODEO-PDE-LDDMM, which used the default parameters given in NODEO-CVPR22 [21].

We considered including IDIR and Nature methods in the benchmark. However, these methods rely on a small-deformation parametrization, which fundamentally differs from the stationary framework used in our proposed methods. Since our study aimed to provide a fair comparison and evaluation within this framework, incorporating IDIR and Nature as benchmarks would not have allowed for a comparison aligned with our study’s objectives.

### 5.3. Implementation Details and Parameters

We performed our experiments on a machine equipped with one NVidia GeForce RTX 4090 with 24 GB of VRAM and an Intel Core i7 with 64 GB of DDR4 RAM. We used the C++ code of the ANTS library for the SyN method. The LDDMM codes were developed on the GPU with Matlab 2024. We used the models available for VoxelMorph, SyMNet, LapIRN, TransMorph, and NODEO-CVPR22 as in [36]. The Python 3.10 codes available in the GitHub repositories https://github.com/yifannnwu/NODEO-DIR (accessed on 31 January 2023),  https://github.com/MIAGroupUT/IDIR (accessed on 1 February 2024), and  https://github.com/BrainImageAnalysis/INRsRegExp (accessed on 1 February 2024) served as the basis for the implementation of our proposed methods.

#### 5.3.1. Benchmark Methods

For the traditional methods, we used the same implementation and parameters as in [29]. In sum, the methods were implemented in a multi-resolution scheme of three levels. Gauss–Newton and Gauss–Newton–Krylov were implemented with an efficient method for the update of the step size based on offline backtracking line-search combined with a check on Armijo’s condition. We used the stopping conditions in [12]. We conducted a search of the optimal parameters in the NIREP16 and OASIS datasets for regularization, selecting those with a good compromise between high DSC and moderate Jacobian extrema values. Importantly, the parameters used for the LDDMM differential operator *L* were α=0.0005 for NIREP and α=0.0010 for OASIS, with s=2.0.

NODEO-CVPR22 was executed with the default parameters. The mean filter was used as a smoothing kernel. The number of time steps was 2, yielding a stationary parametrization. The weighting parameters were $\sigma_{reg}=0.0005$, $\sigma_{grad}=0.05$, and $\sigma_{Jdet}=2.5$. The number of iterations was set to 300. The same parameters were used in the NODEO-LDDMM and NODEO-PDE-LDDMM benchmarks.

#### 5.3.2. Proposed Methods

For the proposed methods, we used the parameters identified in previous works as optimal. Specifically, we used the parameters reported in [18]. Some were selected differently, mainly due to efficiency or convergence issues (marked with ♣).

**Network architecture:** The Siren architecture was composed of three fully connected layers with input and output dimensions of 3 and 256 hidden layers. The 3D + t Siren architecture was composed of three fully connected layers with input and output dimensions of 3 × T, where T = 5. For both, the number of encoding functions was equal to $6^{♣}$ and $\omega_{0}\;=\;30$.

**Loss parameters:** The window size of lNCC was equal to 9. The loss weighting parameters were selected as $\sigma_{reg}=0.05$, $\sigma_{grad}=0.05$, and $\sigma_{Jdet}=100$. The ϵ in $\sigma_{Jdet}$ was selected equal to 0.5. We used the same parameters in NODEO-LDDMM and NODEO-PDE-LDDMM. The benchmark NODEO-CVPR22, NODEO-LDDMM, and NODEO-PDE-LDDMM were evaluated using the parameters proposed in [19]. However, our experimental analysis showed that the proposed loss parameters resulted in improved Jacobian determinants while maintaining competitive registration accuracy for all NODE-based configurations evaluated in this study.

**Coordinate sampler:** The downsizing step size was equal to $4^{♣}$. The patch size was equal to 32 × 32 × 32, although, for some experiments, we note the convenience of using 64 × 64 × 64. Two downsampling and upsampling operations of size 0.5 and 2 were conducted.

**Solvers:** Scaling and Squaring was implemented with t = 10 iterations. Euler and RK were implemented with t = 5 iterations and a time sampling Δt = 0.1. In NODE-based solvers, we selected the time increment to be the same value.

**Optimization:** The Adam optimizer was selected with a learning rate of $1.0\;\times \;10^{-4}$. Different numbers of iterations were considered, although we selected a fixed budget of $300^{♣}$ epochs as optimal. Hybrid sampling was completed with 10 patch-based epochs and 120 samples per epoch. This means that $1200^{♣}$ downsized domains were used for downsize sampling during optimization. Additionally, $1200^{♣}$ patches were randomly sampled for hybrid sampling. Finally, the image domain was used 300 times for the downsample strategy.

## 6. Results

### 6.1. Evaluation Metrics

In this study, we use the widespread Dice similarity coefficient (DSC) for the evaluation of the accuracy of non-rigid registration in the task of atlas-based segmentation [41,42]. As a proxy for transformation quality, we use the invertibility and smoothness of the transformations with different metrics based on the Jacobian determinant of the transformations (in the following, we use the word Jacobian to refer to its determinant). In particular, we use the Jacobian extrema (min and max) and the number of negative Jacobians. The Jacobian maximum allows for measuring the greatest changes in volume; the minimum allows for measuring whether there are foldings in the transformations and how aggressive they are. The number of negative Jacobians allows for measuring whether there is a general tendency to fold or whether foldings occur in a few isolated examples. In our evaluation, we depart from merely showing the results in terms of DSC accuracy or recommending the methods that yield the best DSC metrics. Instead, we analyze the compromise between accuracy and quality depending on the variant, in a similar way as conducted in [17,21,36].

### 6.2. NIREP Results with Downsize, Hybrid, and Downsample-Based Sampling

**Figure 1:**
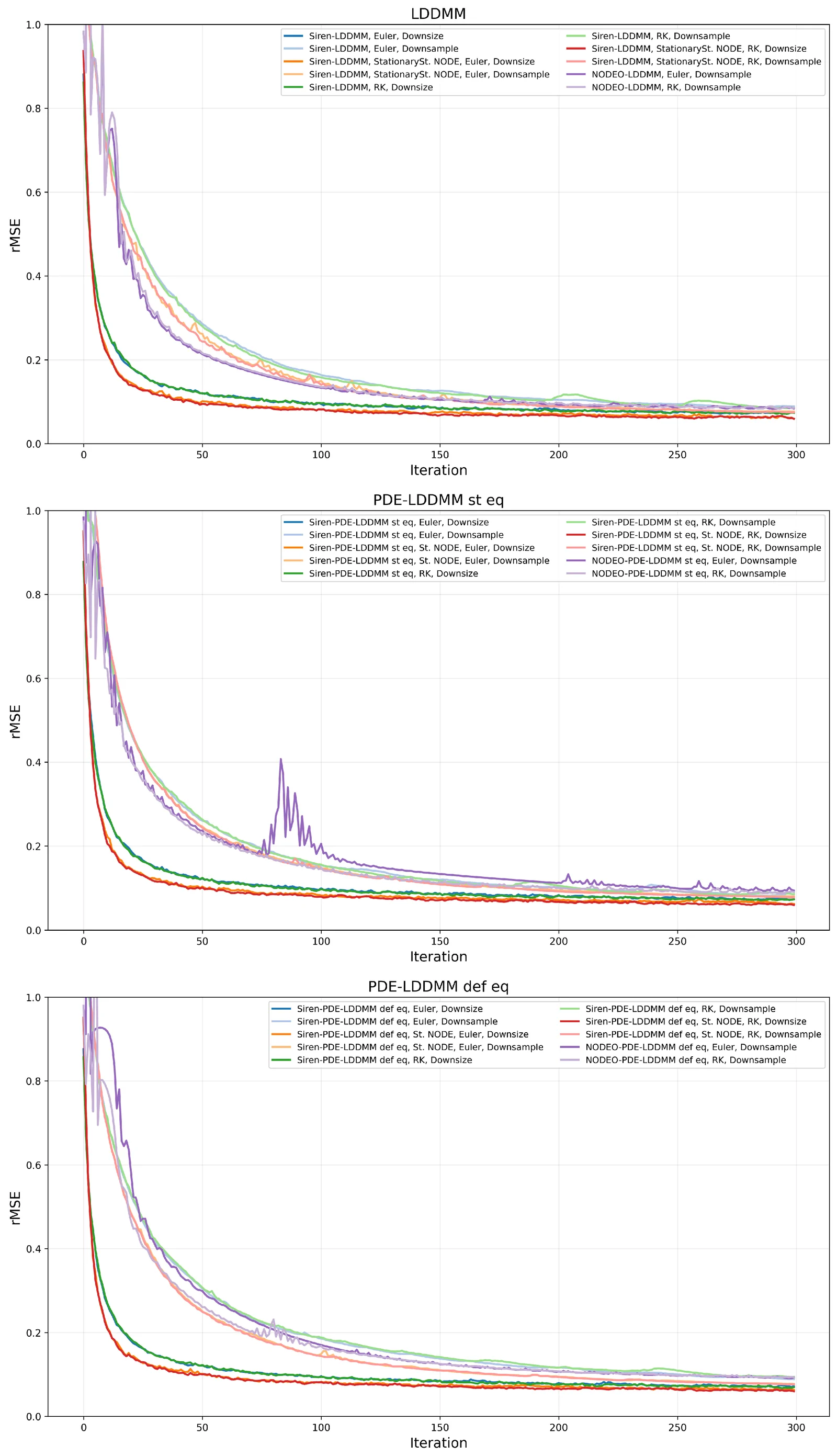
NIREP. Evolution of the relative mean squared error (rMSE) during optimization for a representative NIREP registration experiment. Results are shown for the LDDMM, PDE-LDDMM st eq, and PDE-LDDMM def eq formulations under Euler and RK solvers and downsize and downsample sampling strategies.

Table 3 shows the means and standard deviations of the evaluation metrics obtained after registration for the baseline methods. In addition, Table 4 shows the evaluation metrics after 300 iterations for a selection of variants, leaving aside the worst-performing ones. In particular, we show the results obtained for Siren-LDDMM and Siren-PDE-LDDMM for the downsize, hybrid, and downsample-based sampling strategies and for NODEO-LDDMM and NODEO-PDE-LDDMM for the downsample-based strategy. Tables S1 and S2 in the Supplementary Materials show the results obtained after 100 and 150 iterations. Figure 1 presents representative rMSE convergence curves for the evaluated formulations. The curves exhibit a rapid reduction in the registration error during the first few iterations, followed by a substantially slower convergence rate after approximately 100–150 iterations. Although further improvements are still observed up to 300 iterations, the optimization process appears to approach a plateau, suggesting that the use of a common optimization budget does not substantially affect the comparative evaluation.

**Figure 2:**
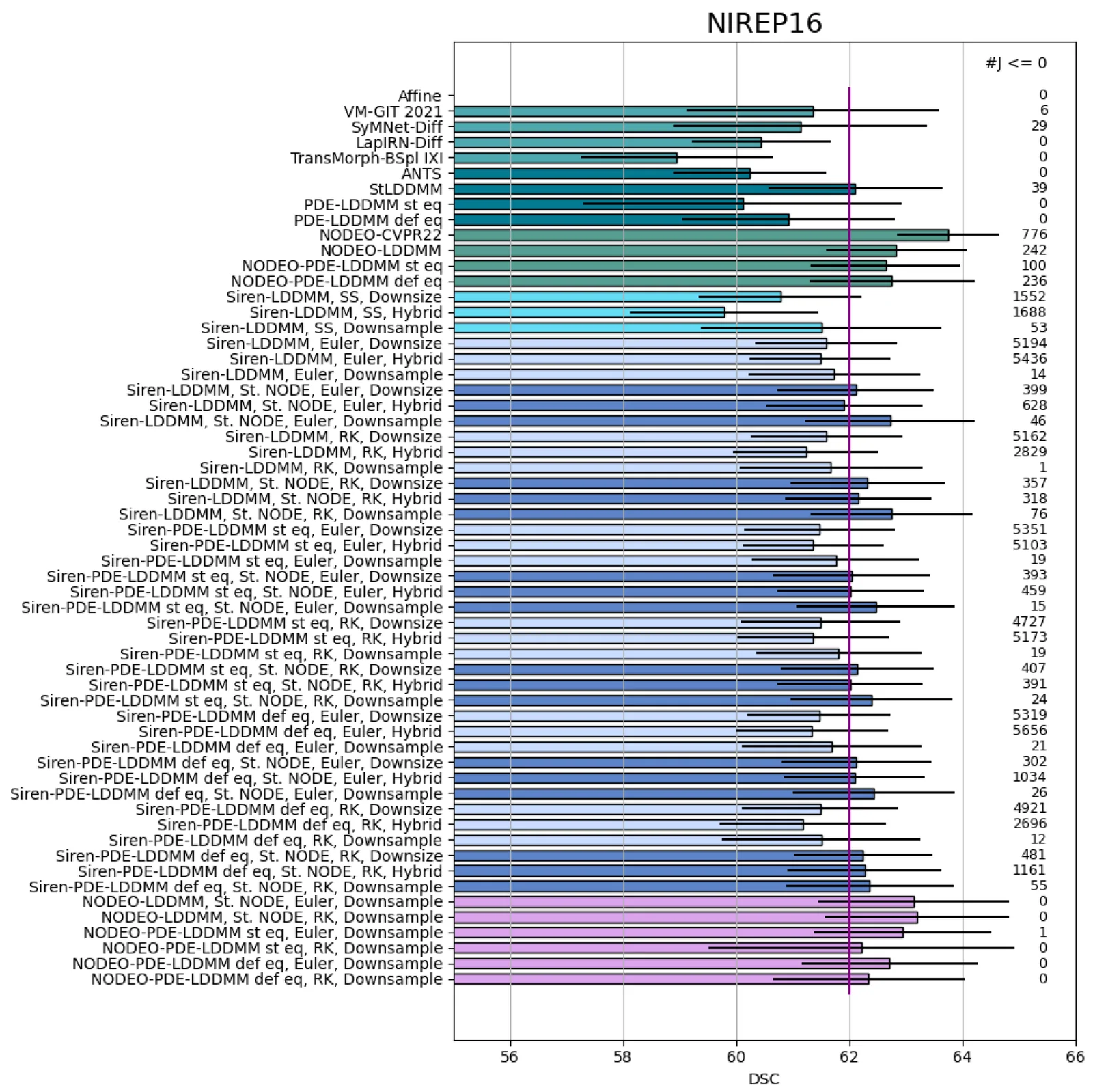
NIREP. Volume overlap obtained by the registration methods, measured in terms of the DSC between the warped and the corresponding manual target segmentations. Error bars show the mean and the standard deviation of the DSC values averaged over the 32 NIREP manual segmentations. The vertical purple line indicates the selected baseline threshold of DSC = 62%, facilitating the comparisons. The color coding in the bars is used to distinguish among deep learning, traditional, Siren-LDDMM, and NODEO-LDDMM methods. In Siren-LDDMM, methods with analogous ODE solvers across groups of variants are indicated using the same blue tone. The bar for affine registration is not visible in the selected range for DSC values. The number of negative Jacobians (#J ≤ 0) is included to better assess the compromise existing between the DSC values and negative Jacobian values for each method.

**Figure 3:**
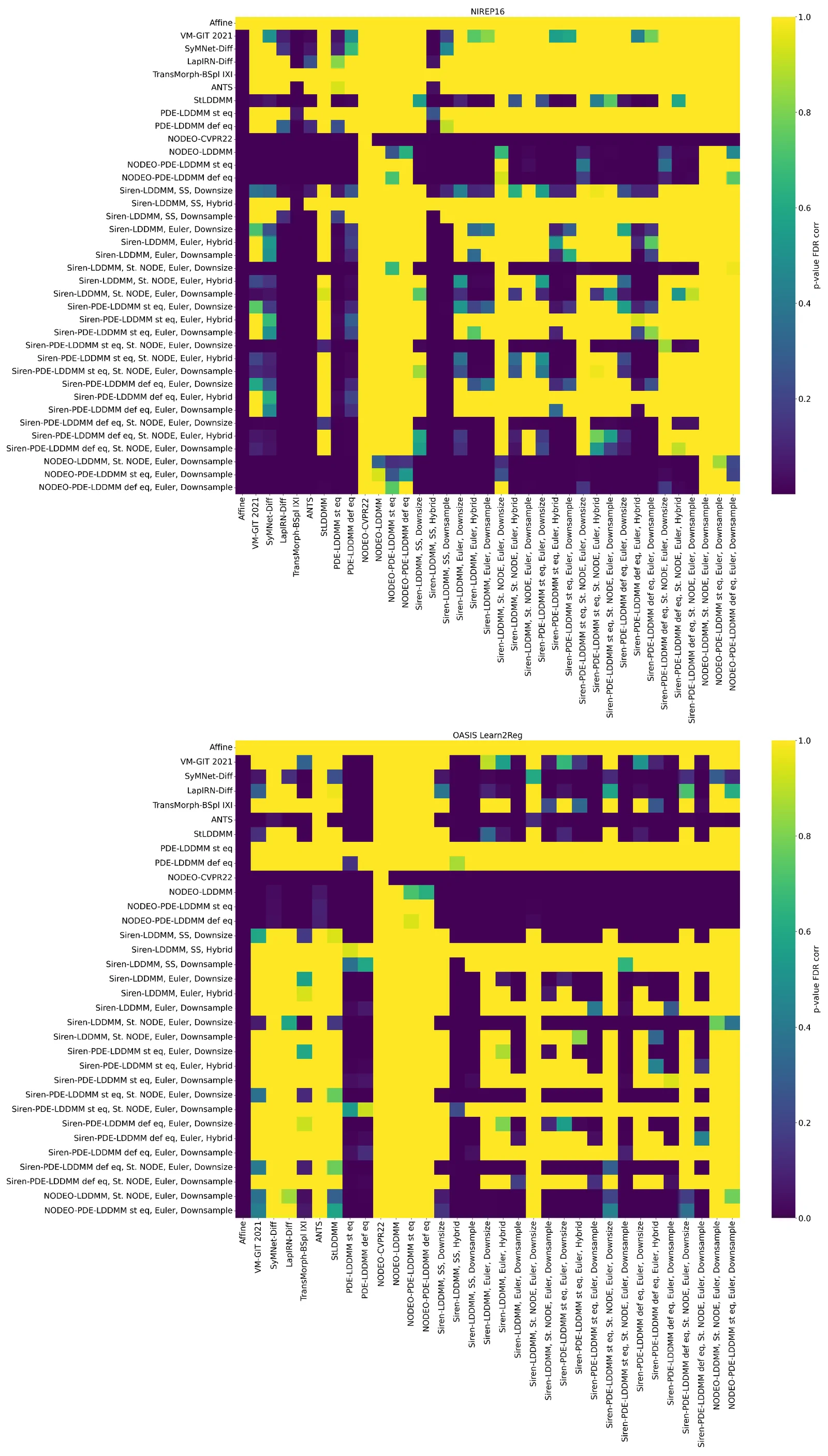
Results of the pairwise right-tailed Wilcoxon rank-sum tests used to assess the statistical significance of the differences among the methods. Scaling and Squaring and Euler solvers. Each matrix entry represents the pairwise *p*-values between every two methods with false discovery rate (FDR) correction, obtained when testing whether the method corresponding to the row achieved higher DSC values than the method corresponding to the column. Dark blue cells indicate statistically significant evidence in favor of the row method (low *p*-values), whereas lighter colors indicate insufficient evidence to reject the null hypothesis. A straightforward interpretation of the plots is that rows containing extended blue regions correspond to methods that significantly outperform a larger number of competing approaches.

The tables show that the DSC performance increases with the number of iterations. After 100 or 150 iterations, neither Siren-LDDMM nor Siren-PDE-LDDMM is able to surpass the baseline threshold of DSC = 62%, surpassed by the best-performing baseline methods. The number of variants reaching the threshold considerably increases after 300 iterations. NODEO-PDE-LDDMM also reaches the baseline threshold exclusively after 300 iterations. Therefore, we proceed with the analysis of the results obtained after 300 iterations. To facilitate comparisons, Figure 2 shows the DSC values of the baseline methods (Table 3) and the studied methods (Table 4) in the shape of error bars. In addition, Figure 3 shows the results of pairwise right-tailed Wilcoxon rank-sum tests that were conducted for the assessment of the statistical significance of the difference in medians for the distribution of the DSC values. The alternative hypothesis is that the median of the first distribution is higher than the median of the second one. The figure focuses on Scaling and Squaring and Euler solvers. The complete results, including RK solvers, are shown in the Supplementary Materials, Figure S1.

Both SIREN and NODE methods outperformed the DSC baselines established by the best-performing deep learning approaches (VM-GIT, SyMNet-Diff, LapIRN-Diff, TransMorph-BSpl IXI) and traditional benchmarks (ANTS, St-LDDMM, PDE-LDDMM) in certain combinations of sampling strategies and ODE solvers. This is clearly illustrated in Figure 2 for the methods with error bars surpassing the baseline threshold of DSC = 62%. The results of the pairwise right-tailed Wilcoxon rank-sum tests in Figure 3 showed statistical significance, coherent with all our observations of DSC-based superiority ($p<1.0\;\times \;10^{-3}$).

Regarding SIREN methods with SS, Euler, and RK solvers, all strategies produced DSC values below the baseline threshold. Scaling and Squaring slightly underperformed the other solvers for the downsize and hybrid strategies. The differences between Euler and RK solvers were negligible in all cases. The number of negative Jacobians reached several thousand for the downsize and hybrid strategies. On the other hand, downsampling strategies yielded DSC values that were slightly higher than for downsizing for each variant configuration. For these, statistical significance was found (compare two pixels left from the diagonal of the downsize methods in Figure 3). The number of negative Jacobians was significantly lower (typically only a few dozen). Combining SIRENs with NODE solvers produced competitive DSC values above the baseline threshold. Statistical significance in the DSC values was found in the majority of comparisons with SIREN methods without NODE solvers. Among them, the downsampling strategy outperformed the others and maintained an acceptable min (J)–max (J) range and a low number of negative Jacobians.

Regarding NODE methods, the number of negative Jacobians shown in Table 4 was significantly reduced compared to the baseline given in Table 3 thanks to the hyperparameter selection used in this work. The reduction is primarily associated with the use of stronger regularization parameters ($\sigma_{reg}$ and $\sigma_{Jdet}$), which promote geometrically consistent deformations and reduce folding artifacts. All variants surpassed the baseline threshold. In particular, NODEO-LDDMM using RK emerged as the second-best-performing method, with a DSC of 63.20% and no negative Jacobians. It was outperformed only by NODEO-CVPR22, with a DSC of 63.74% but with 776 negative Jacobians. The differences between Euler and RK solvers were also not relevant in all cases. The pairwise right-tailed Wilcoxon rank-sum tests showed statistical significance for the superiority of NODE methods in terms of DSC-based registration accuracy with respect to most baseline methods and SIREN variants without NODE solvers.

The most time-efficient strategy was downsizing, showing computation times below 100 s in the great majority of SIREN variants after 300 iterations. However, the complexity increased significantly when NODE was combined with ODE solvers. Hybrid sampling combined with Euler or RK solvers was the second most efficient strategy. For the SIREN variants using NODE solvers, downsampling was the third most efficient strategy. However, the most time-efficient variants were obtained for the combination of CNNs, downsampling, and NODEs. Despite the time efficiency of downsizing, patch-based strategies significantly increased the complexity of hybrid resizing, even for a small number of iterations. These results are complemented in the Supplementary Materials with Table S3, regarding the maximum VRAM memory usage achieved by the registration methods.

**Figure 4:**
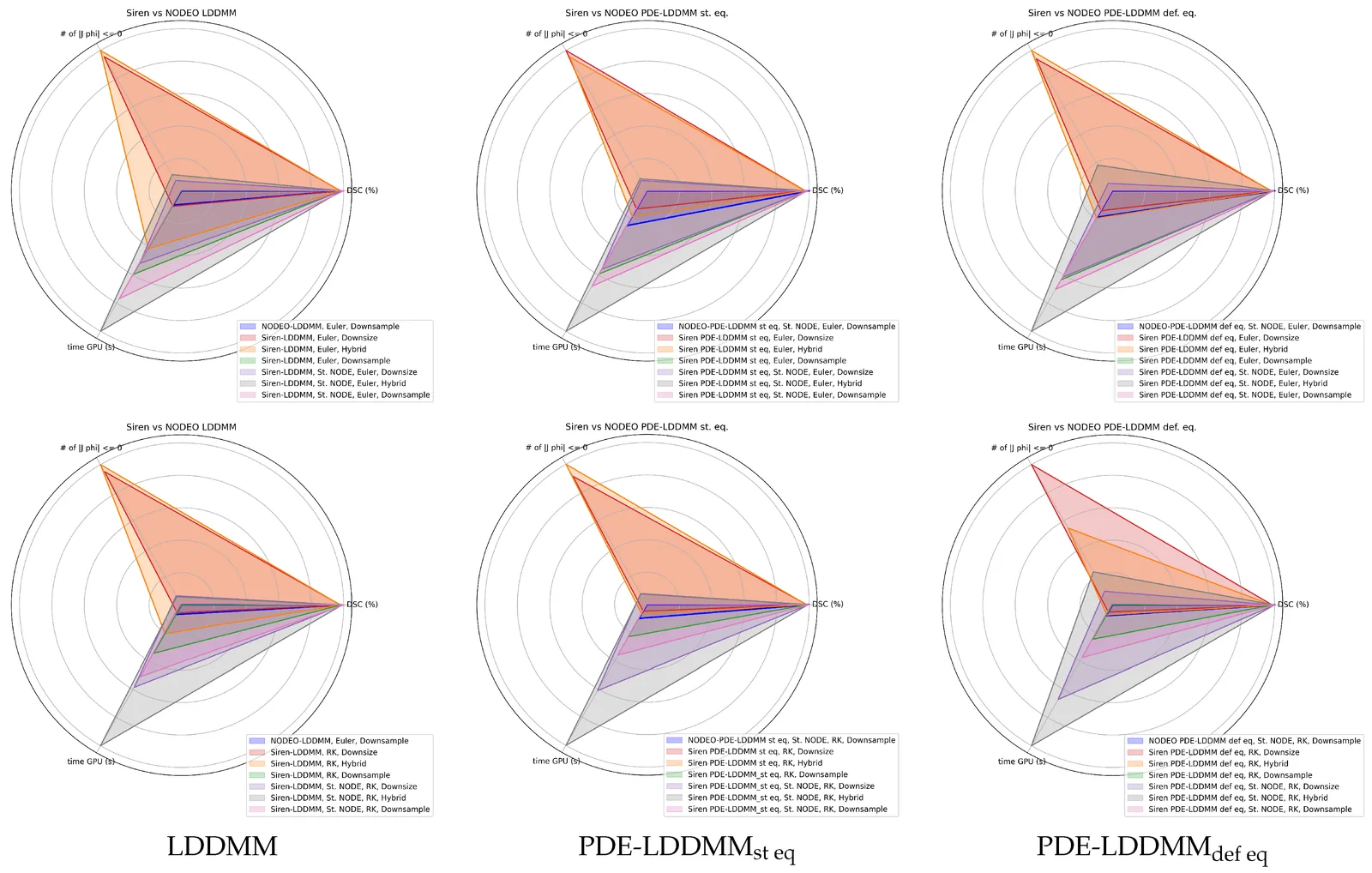
NIREP. Spider charts comparing the mean DSC, number of negative Jacobians (#J ≤ 0), and computation time of the results presented in Table 4. Charts are grouped by LDDMM variant and ODE solver. The upper row corresponds to the Euler method, while the lower row corresponds to the RK method.

Figure 4 presents a visual multi-objective comparison of the DSC, the number of negative Jacobians, and the computation time in the shape of spider charts. To ease the comparisons, the results are split in terms of the LDDMM variant (LDDMM, PDE-LDDMM st. eq., or PDE-LDDMM def. eq.) and the ODE solver (focusing on Euler or RK). NODEO-LDDMM and NODEO-PDE-LDDMM generally exhibit the most balanced trade-offs between registration accuracy, Jacobian positive values, and computational cost. The problems with the high number of negative Jacobians for the downsize and hybrid strategies become more evident in the chart. The compromise between the DSC and the number of negative Jacobians for SIRENs with downsample and NODEs is similar to that of their NODEO counterparts. However, the computational complexity is much higher, as already highlighted in the tables.

### 6.3. NIREP Failed Cases

Some of the variants listed in Table 2 did not achieve competitive results in certain metrics considered in this study. This section highlights the most relevant results to better illustrate the issues with the specific failing combinations of sampling strategies, ODE solvers, or architecture design.

#### 6.3.1. NIREP Results with Patch-Based Sampling

The experiments with patch-based sampling from scratch did not converge for patches of a size inferior to 64 × 64 × 64. Table 5 presents the evaluation results obtained using patch-based sampling methods of size 64 × 64 × 64. The table shows that, even after 300 iterations, the registration accuracy measured in terms of the DSC remained far from an acceptable baseline. The Jacobian extrema indicate the moderate local contraction and expansion of the transformations, resulting in few or no negative Jacobians. The computational time is on the order of hours, exceeding 10,000 s in the cases involving LDDMM. The results for the NODE-based version are excluded due to its extremely high computational demands in both time and memory. These results suggest that patch-based sampling should be utilized in hybrid sampling with a reduced number of iterations to ensure a computationally feasible workload.

#### 6.3.2. NIREP Results with Hybrid-Based Sampling and NODE-Based Solvers

The lack of convergence of the patch-based methods with patch sizes of less than 32 × 32 × 32 motivated us to obtain results with hybrid sampling and a patch size of 64 × 64 × 64. Table 6 shows the results obtained after 300 iterations for traditional ODE solvers. In this case, SIREN methods produced more competitive DSC values above the baseline threshold. However, the number of negative Jacobians still reached several thousand. The computational time increased with respect to smaller patch sizes. Regarding NODE-based solvers, Table S4 in the Supplementary Materials shows the computational time and the maximum memory load achieved by the registration methods with hybrid-based sampling in one representative experiment. In some cases, it can be appreciated that the resulting variant requires the order of GPU hours, which leads to unacceptable time complexity for a pairwise method given current standards.

#### 6.3.3. NIREP Results with LDDMM Operator as the Final Layer

Table 7 shows the evaluation metrics for the variants corresponding to the methods in Table 4 when swapping the kernel layer between ${\left(L^{†}L\right)}^{-1}$ and the identity. Interestingly, the Siren-LDDMM variants with the identity outperformed those with the LDDMM operator, while, for NODEO-LDDMM, the variants with the LDDMM operator performed better. In particular, the Siren-LDDMM and Siren-PDE-LDDMM variants greatly underperformed the baseline methods in terms of the DSC values. This was also the case with NODEO-LDDMM and NODEO-PDE-LDDMM_def eq_. NODEO-PDE-LDDMM_st eq_ showed the highest DSC values, reaching an impressive 65%, but this was at the cost of a huge number of negative Jacobians, in the order of ten thousand.

### 6.4. NIREP Qualitative Results

**Figure 5:**
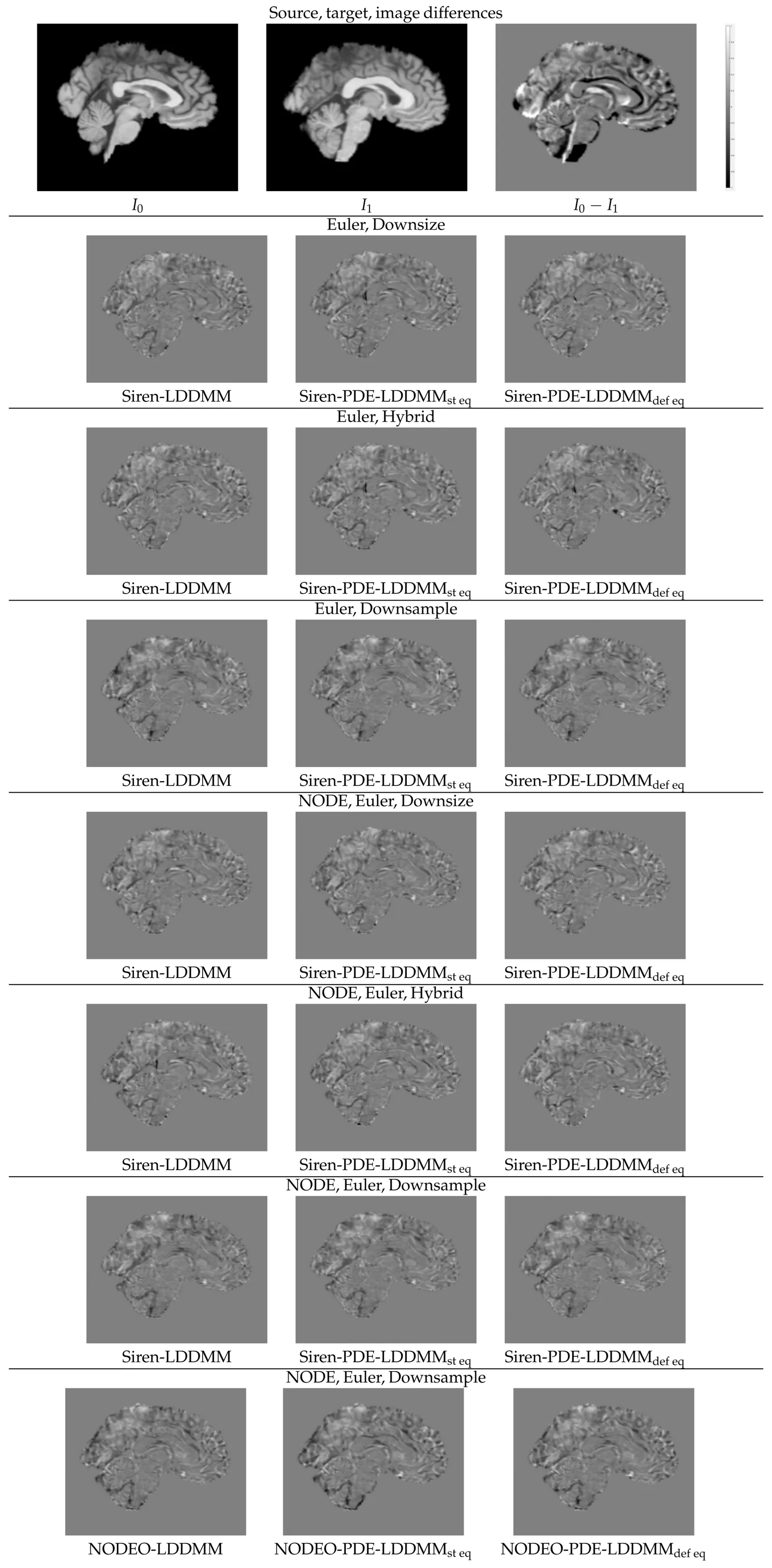
NIREP. Sagittal views of the differences after registration $I_{0}\circ \varphi^{-1}-I_{1}$ in a representative experiment. SIREN and NODE methods with Euler and NODE-Euler solvers.

Figure 5 shows sagittal views of the differences after the registration of the methods with Euler and NODE-Euler solvers. The Supplementary Materials show the results obtained with RK and NODE-RK solvers, Figures S4–S6. The methods effectively reduced visual discrepancies after registration in a comparable way. The lowest image similarity is observed in the parietal and occipital lobes, as well as in the cerebellum.

**Figure 6:**
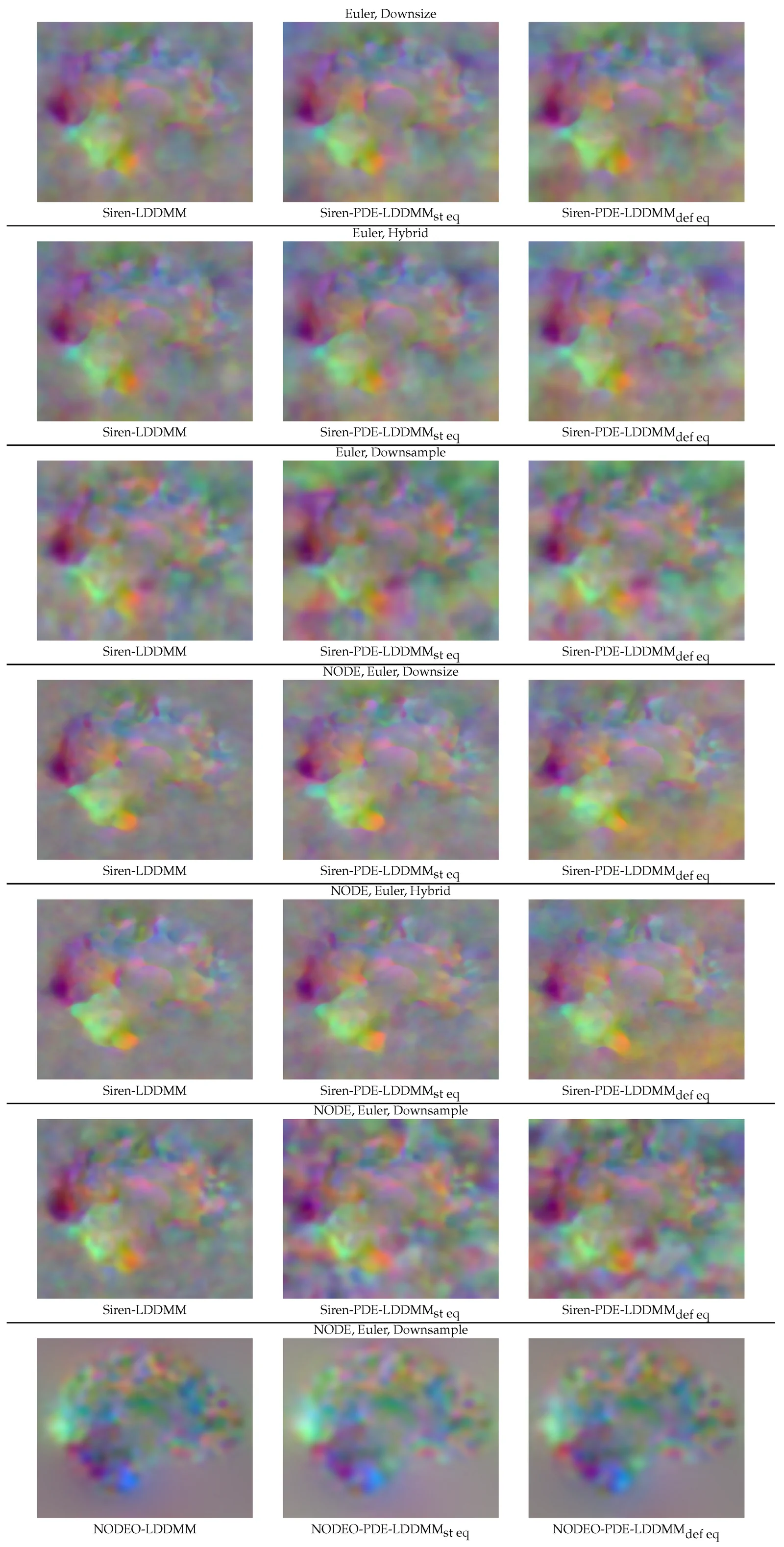
NIREP. Sagittal views of the displacement fields in a representative experiment. The RGB color map proposed in the VoxelMorph paper is used for the color representation of the vector fields. SIREN and NODE methods with Euler and NODE-Euler solvers.

**Figure 7:**
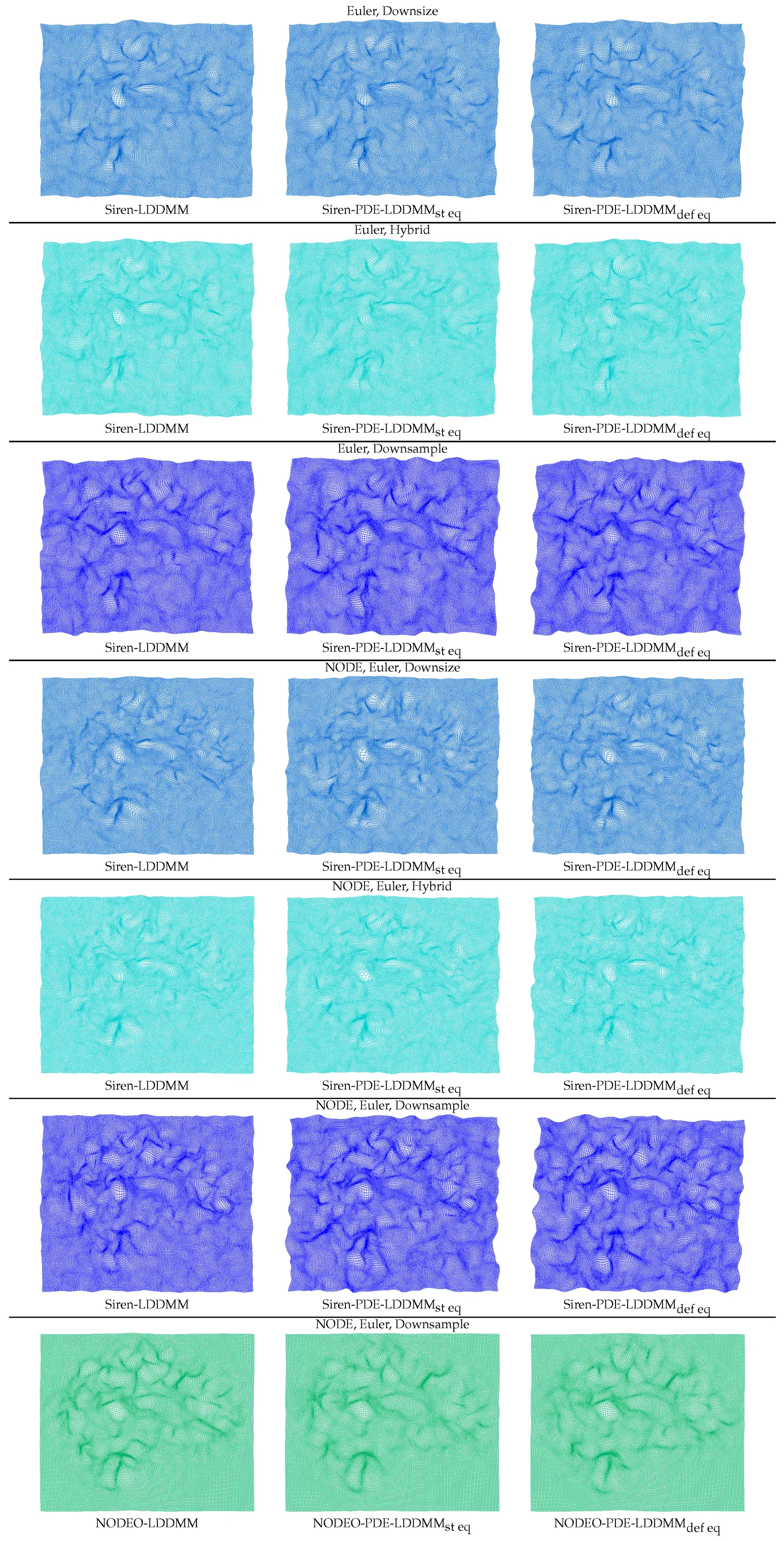
NIREP. Sagittal views of the transformation grids in a representative experiment. SIREN and NODE methods with Euler and NODE-Euler solvers.

Figure 6 and Figure 7 present sagittal views of the displacement fields and the transformation grids of the different variants. The results obtained with the baseline methods are provided in the Supplementary Materials, Figures S2 and S3. All displacement fields appear visually smooth. For each sampling strategy and ODE solver, the displacement fields of the three Siren-LDDMM and NODEO-LDDMM variants exhibit similar characteristics. However, the color coding of the displacement fields differs significantly between SIREN and NODE methods. In particular, the displacement field estimated in the background is closer to zero in NODEO compared to SIREN methods, suggesting that the filling-in effect is more plausible for the NODEO-LDDMM and NODEO-PDE-LDDMM variants.

The visual exploration of the transformation grids provides more informative insights than the visualization of the displacement fields. Subtle differences among the three Siren-LDDMM and NODEO-LDDMM variants can be observed across the cortex. In this case, the grids of all SIREN and NODE methods exhibit significant differences. The background deformation previously noted in the displacement field images is more clearly visible in the grids of the SIREN methods. The impact of the negative Jacobians obtained with the downsize sampling strategy does not result in visible grid defects.

### 6.5. OASIS Results with Downsize, Hybrid, and Downsample-Based Sampling

**Figure 8:**
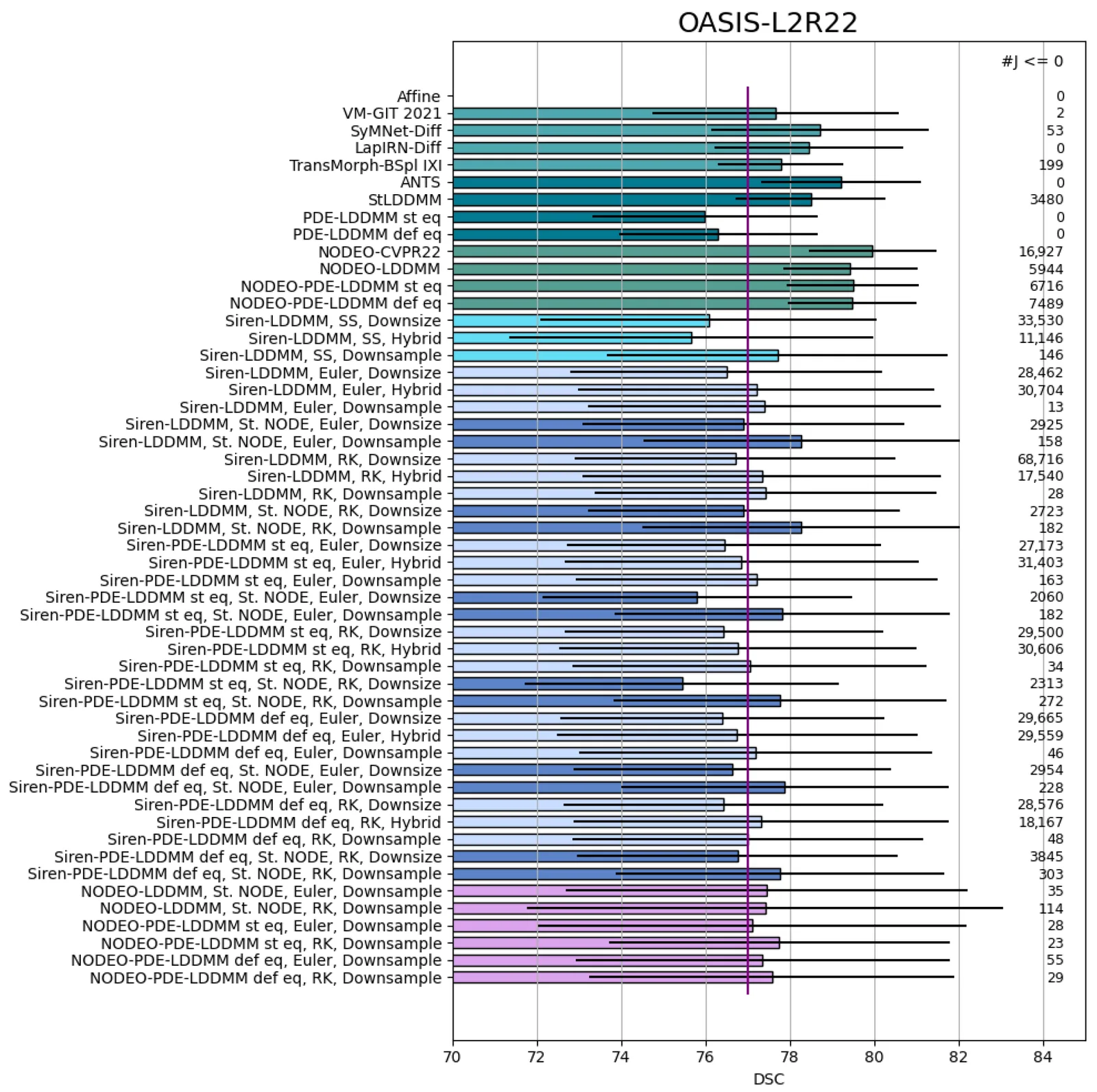
OASIS. Volume overlap obtained by the registration methods, measured in terms of the DSC between the warped and the corresponding manual target segmentations. Error bars show the mean and the standard deviation of the DSC values averaged over the 35 OASIS segmentations. The vertical purple line indicates the selected baseline threshold of DSC=77%, facilitating the comparisons. The color coding in the bars is used to distinguish among deep learning, traditional, Siren-LDDMM, and NODEO-LDDMM methods. In Siren-LDDMM, methods with analogous ODE solvers across groups of variants are indicated using the same blue tone. The bar for affine registration is not visible in the selected range of DSC values. The number of negative Jacobians (#J≤0) is included to better assess the compromise existing between the DSC values and negative Jacobian values for each method.

Table 8 shows the means and standard deviations of the evaluation metrics obtained after registration for the baseline methods. In addition, Table 9 shows the evaluation metrics obtained after 300 iterations for the same variants selected from the NIREP results. In this case, hybrid experiments were conducted with patch sizes of 64 × 64 × 64. Otherwise, we experienced convergence issues, preventing the methods from obtaining acceptable results. Figure 8 shows the DSC values of the baseline and studied methods in the shape of error bars, and Figure 3 shows the results of the pairwise right-tailed Wilcoxon rank-sum tests for the Scaling and Squaring and Euler solvers. The complete results, including RK solvers, are shown in the Supplementary Materials, Figure S1.

Among the baseline methods, the best-performing method was ANTS, with a mean DSC of 79.21 and no negative Jacobians. NODE methods reached similar DSC values; however, the number of negative Jacobians reached several thousand. The results of the pairwise right-tailed Wilcoxon rank-sum tests, shown in Figure 3, indicated statistical significance between ANTS and non-benchmark NODE methods ($p<1.0\times 10^{-3}$).

From the SIREN methods, the best DSC values were obtained for the variants with NODE and downsampling strategies. The Wilcoxon rank-sum tests showed statistical significance. The number of negative Jacobians was controlled, reaching several hundred. The second-best DSC values were obtained for the variants with downsampling strategies. The number of negative Jacobians rose to a few dozen. Downsize and hybrid strategies not only yielded slightly lower mean DSC values in the majority of cases but also obtained a number of negative Jacobians that was disproportionately large, reaching several tens of thousands. Among NODE methods, the DSC values were slightly lower than for SIREN with NODE and downsampling strategies, but the number of negative Jacobians was controlled to a few dozen. The Wilcoxon rank-sum tests indicated statistically significant differences for most methods yielding lower DSC values than NODEO.

### 6.6. OASIS Qualitative Results

**Figure 9:**
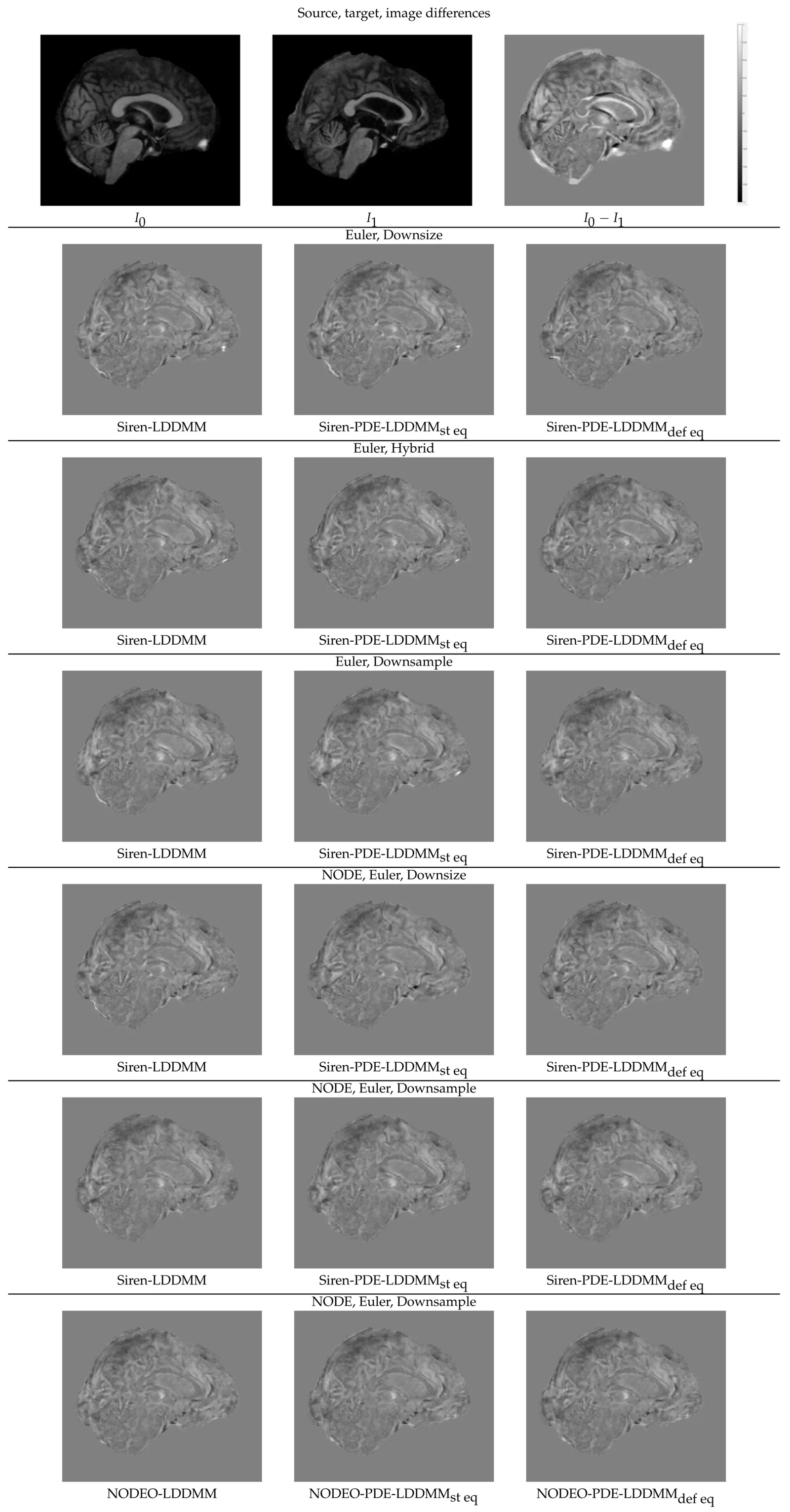
OASIS. Sagittal views of the differences after registration $I_{0}\circ \varphi^{-1}-I_{1}$ in a representative experiment. SIREN and NODE methods with Euler and NODE-Euler solvers.

Figure 9 shows sagittal views of the differences after the registration of the methods with Euler and NODE-Euler solvers. The Supplementary Materials show the results obtained with RK and NODE-RK solvers, Figures S7–S9. As with NIREP, all methods reduced the visual discrepancies in a comparable way. In this case, the major differences are observed in the parietal lobe. Despite the differences between the images in the corpus callosum and the lateral ventricle area, all methods were able to strongly align both structures with visual accuracy.

**Figure 10:**
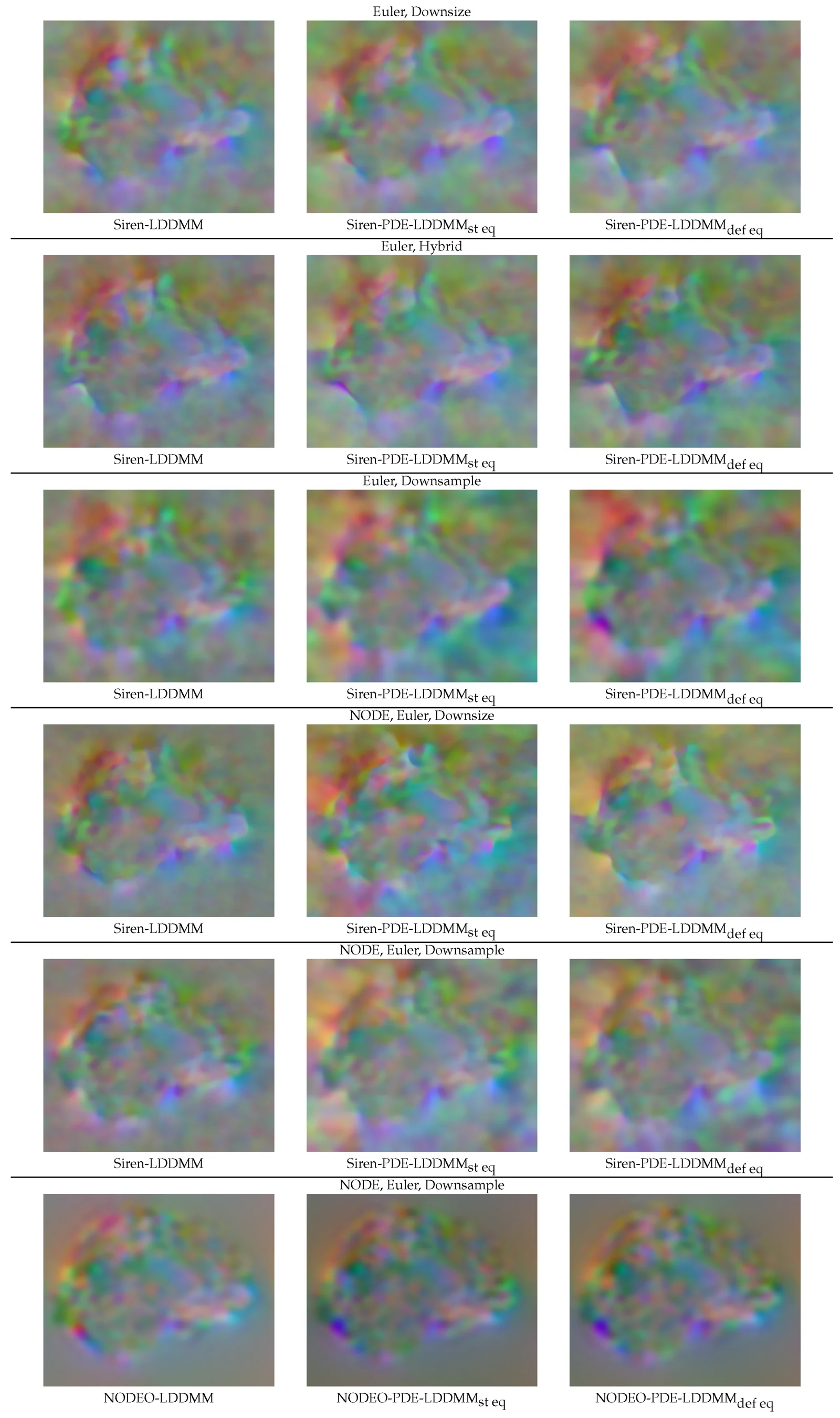
OASIS. Sagittal views of the displacement fields in a representative experiment. The RGB color map proposed in the VoxelMorph paper is used for the color representation of the vector fields. SIREN and NODE methods with Euler and NODE-Euler solvers.

**Figure 11:**
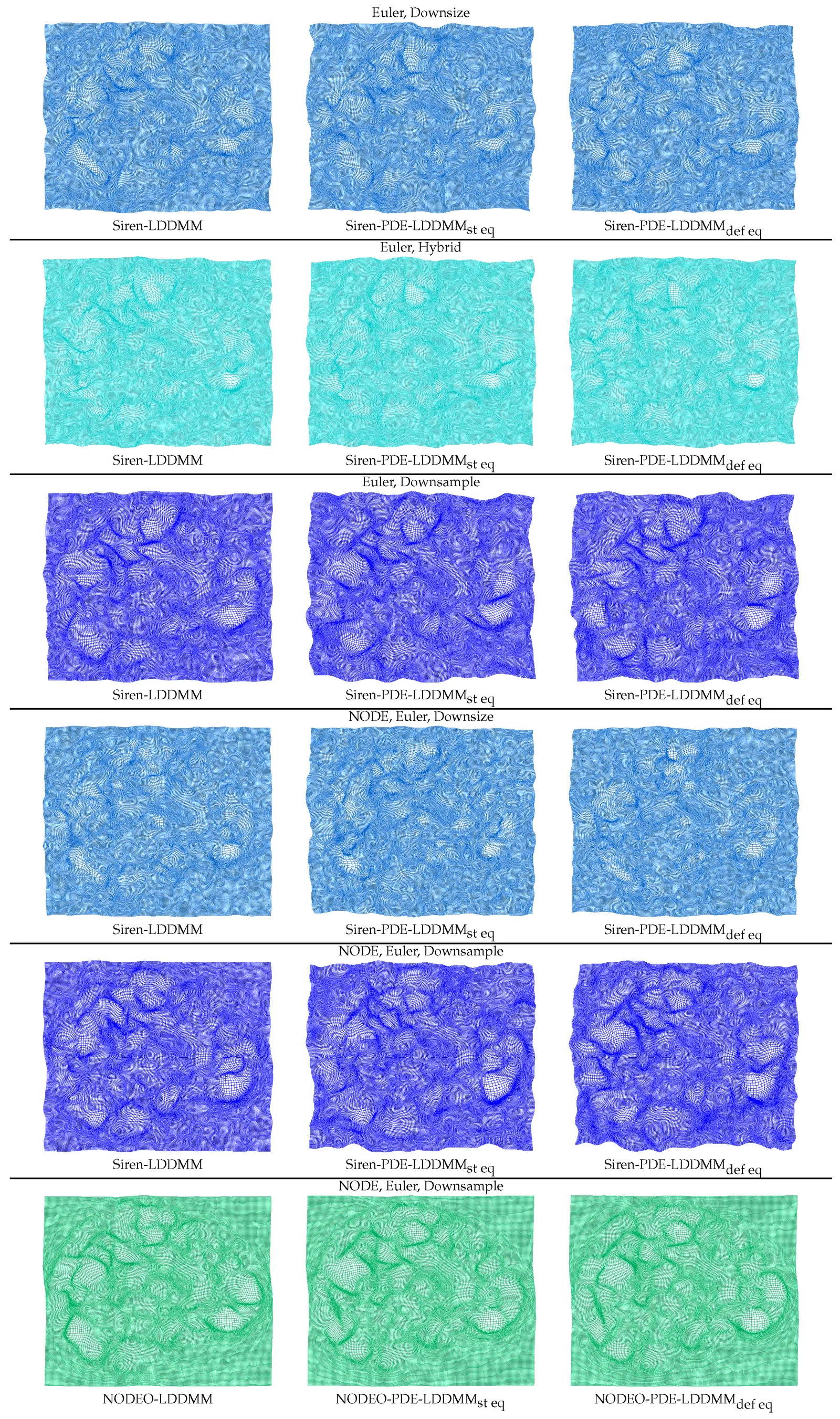
OASIS. Sagittal views of the transformation grids in a representative experiment. SIREN and NODE methods with Euler and NODE-Euler solvers.

Figure 10 and Figure 11 present sagittal views of the displacement fields and the transformation grids of the different variants. As with NIREP, all displacement fields appear visually smooth. The color coding of the displacement fields differs significantly not only between SIREN and NODE methods but also among LDDMM variants. The displacement field estimated in the background is more variable for SIREN methods. The visual exploration of the transformation grids reveals differences observed with the displacement fields across architectures and the LDDMM methodology. Some of the negative Jacobian areas for the downsize and hybrid strategies can be appreciated in the grids, although they do not impact the global smooth appearance seen across the grids.

## 7. Discussion

In recent years, the rapid development of learning-based methods has led to a growing number of approaches for non-rigid and diffeomorphic image registration. While this progress has expanded the range of available techniques, it has also made it increasingly challenging to obtain clear and fair comparisons between methods. When a novel approach is proposed, it is essential to assess its advantages and limitations thoroughly, as well as to ensure consistency and fairness in experimental evaluations. However, this is often hindered by differences in datasets, implementations, and experimental protocols. In addition, the complexity of these models can make it difficult to develop a clear theoretical understanding and to establish meaningful connections between different formulations. These challenges highlight the need for systematic and controlled studies. In this context, our work revisits the use of implicit neural representations for non-rigid registration, proposes extensions within the LDDMM and PDE-LDDMM frameworks, and provides a comprehensive and unbiased evaluation of different variants to identify those most suitable for practical applications.

### 7.1. Unifying Framework for INRs and NODEOs

IDIR was first proposed in 2022 for non-rigid registration using INRs within the small deformation paradigm [16]. In the same year, NODEO was introduced for diffeomorphic registration using NODEs in the solution of the transport equation [19]. Both methods employ neural networks to model functions that yield an implicit representation of either the displacement fields or the velocity fields in the transport equation. While INRs evaluate the learned functional representation of the displacements at specific spatial points, NODEs capture the temporal dynamics of the learned functional representation of the right-hand side of an ODE, enabling the model to represent time-dependent changes. This common underlying principle allows INRs and NODEO to be considered from the same family of methods, bringing NODEO within the broader family of INR-based methods.

Indeed, the MLP architecture and accompanying components from INRs can be integrated into the NODEO problem formulation. This idea was first proposed in [18] as part of the INR family. However, the comparison with the original NODEO formulation was limited, making it difficult to draw clear conclusions about the relative performance of the methods. In addition, some of the claims regarding the behavior of NODEO were not fully supported by the experimental evidence presented. In contrast, our work brings together successful implementations of both families of methods and their combinations.

### 7.2. MLPs Architectures and Computational Complexity

From our study of related work, we observed that using MLP architectures introduces significant computational complexity due to the fully connected layers and the spatial encoding used to enrich the input representation. In particular, memory constraints force these methods to rely on sampling strategies during training, such as downsizing or patch-based sampling. During inference, the image pairs need to be processed sequentially in small blocks, even though the memory burden associated with storing gradients is no longer present. Interestingly, despite their computational demands, these methods are often described as efficient in the literature [16]. Some recent articles acknowledge the time complexity as the main limitation of INRs but provide little specific detail [18]. Our work demonstrates that, despite the use of sampling strategies that effectively reduce the high computational complexity to a more manageable level, some variants remain prohibitively expensive in terms of time complexity.

### 7.3. Sampling Strategies

Both SIREN- and NODEO-based methods require the use of sampling strategies to reduce the computational complexity inherent to INRs. Both methods work under the assumption that the neural implicit representations can be accurately estimated from a reduced-size domain. For SIREN and the downsizing strategy, the regularization losses are computed by estimating differentials using points that are considerably separated with respect to the separation in the original domain. As a result, the losses do not accurately enforce smoothness constraints, increasing the risk of negative Jacobians at finer resolutions. We observed that this effect was mitigated with the use of NODE solvers. In contrast, the downsampling strategy includes downsizing within the network, ensuring that differentials are computed directly in the original domain. As a result, negative Jacobians are more controlled. However, this comes at the cost of losing the domain size independence afforded by the downsizing and hybrid strategies.

### 7.4. Factors Driving the Performance Improvements

An important observation arising from the ablation experiments is that the performance differences cannot be attributed to a single factor in isolation. The introduction of NODE formulations consistently improves the performance of the corresponding non-NODE configurations. Downsample sampling consistently outperforms the downsize and hybrid strategies, with the largest gains generally observed when combined with NODE formulations. The kernel swap experiments reveal that the effect of the operator ${\left(L^{†}L\right)}^{-1}$ is strongly architecture-dependent. While it substantially benefits the CNN-based NODEO formulations, it degrades the performance of the SIREN-based models. The best results are obtained from the combination of NODE formulations, downsample sampling, and the CNN-based architecture equipped with the LDDMM-inspired regularization strategy, which consistently provides the most favorable balance between registration accuracy, geometric consistency, and computational efficiency.

## 8. Conclusions

In this work, we have presented a unified formulation of implicit neural representations (INRs) and neural ODE-based approaches within the LDDMM and PDE-LDDMM frameworks for diffeomorphic image registration. This formulation enables a systematic and controlled comparison of different neural representations for continuous transformation modeling. Through an extensive empirical analysis, we have characterized the behavior of a wide range of methodological variants, including different neural architectures, sampling strategies, and ODE solvers. Our results highlight important trade-offs between computational efficiency, geometric consistency, and registration accuracy.

In particular, we show that INR-based formulations introduce non-trivial challenges related to computational complexity and the use of sampling strategies. While these strategies are necessary to make training and inference tractable, they can negatively affect transformation smoothness and diffeomorphic consistency, especially at higher resolutions. In contrast, NODE-based formulations demonstrate more stable and consistent behavior across different configurations, providing a better balance between computational cost and geometric properties. These findings provide a clearer understanding of the strengths and limitations of implicit neural representations for diffeomorphic registration and offer practical guidance for the design of future methods in continuous transformation modeling and related signal processing applications.

Future work will focus on improving the regularization of SIREN-based variants with downsizing strategies and on optimizing the computational efficiency of high-performing methods through improved solver implementations and alternative architectures. In addition, the proposed framework will be extended to multimodal registration settings. These directions may further facilitate the integration of advanced diffeomorphic registration techniques into practical computational anatomy applications.

## Figures and Tables

**Table 1:** Distinctive features of non-rigid image registration methods based on implicit neural representations (INRs). The parametrizations are divided into small deformations or stationary velocity fields. With $u_{\theta }$, we denote the INR over the displacement field *u*, while $v_{\theta }$ denotes the INR over the velocity field *v*. With rhs, we denote the right-hand-side of a differential equation. K-CNN indicates a CNN with a final layer applying a Gaussian Kernel. LDDMM-CNN indicates a CNN with a final layer applying the LDDMM operator ${\left(L^{†}L\right)}^{-1}$. The symbol ♠ indicates that the codes are available in GitHub repositories. n.a. means that no ODE solver is involved in the method.

Method	Parametrization	INR	Sampling	ODE Solver
IDIR [16] ♠	small def.	$u_{\theta }$ with MLP	Random	n.a.
Nature [17] ♠	small def.	$u_{\theta }$ with MLP	Patch	n.a.
DNVF [20]	st. vel.	$v_{\theta }$ with CNN	Downsize	Scaling and Squaring
MIRNF [18]	small def.	$u_{\theta }$ with MLP	Patch/Downsize /Hybrid	n.a.
MIRNF [18]	st. vel.	$v_{\theta }$ with MLP	Downsize /Hybrid	NODE Runge–Kutta
NODEO [19] ♠	st. vel.	$v_{\theta }$ with K-CNN	Downsample	NODE Euler
NODEO-LDDMM [21]	st. vel.	$v_{\theta }$ with LDDMM-CNN	Downsample	NODE Euler
NODEO- PDE-LDDMM [21]	st. vel.	rhs with LDDMM-CNN	Downsample	NODE Euler
NePhi [22] ♠	small def.	$u_{\theta }$ with MLP	Random	n.a.

**Table 2:** Summary of the 58 LDDMM and PDE-LDDMM variants evaluated in this work, detailing the INR model, domain sampling strategy, and ODE solver. Note that the INRs without downsizing may include the operator $K={\left(L^{†}L\right)}^{-1}$ in the architecture, yielding two different methods with a slight difference in the implementation. The column *Closest Method* highlights the variants resembling existing approaches in the literature. Of the 58 variants, ten correspond to or are closely related to existing methods, while the remaining are novel, to the best of our knowledge.

n	LDDMM Variant	INRs	Sampling	ODE Solver	Closest Method
1	LDDMM	$v_{\theta }$ with Siren	Downsize	SS	DNVF [20]
2	LDDMM	$v_{\theta }$ with Siren	Patch	SS	-
3	LDDMM	$v_{\theta }$ with Siren	Hybrid	SS	-
4	LDDMM	$v_{\theta }$ with (id or K)-Siren	Downsample	SS	-
5	LDDMM	$v_{\theta }$ with 3D+t Siren	Downsize	Euler	-
6	LDDMM	$v_{\theta }$ with 3D+t Siren	Patch	Euler	-
7	LDDMM	$v_{\theta }$ with 3D+t Siren	Hybrid	Euler	-
8	LDDMM	$v_{\theta }$ with 3D+t (id or K)-Siren	Downsample	Euler	-
9	LDDMM	$v_{\theta }$ with 3D+t Siren	Downsize	RK	-
10	LDDMM	$v_{\theta }$ with 3D+t Siren	Patch	RK	-
11	LDDMM	$v_{\theta }$ with 3D+t Siren	Hybrid	RK	-
12	LDDMM	$v_{\theta }$ with 3D+t (id or K)-Siren	Downsample	RK	-
13	LDDMM	$v_{\theta }$ with Siren	Downsize	NODE-Euler	-
14	LDDMM	$v_{\theta }$ with Siren	Patch	NODE-Euler	-
15	LDDMM	$v_{\theta }$ with Siren	Hybrid	NODE-Euler	-
16	LDDMM	$v_{\theta }$ with (id or K)-Siren	Downsample	NODE-Euler	NODEO-LDDMM [21]
17	LDDMM	$v_{\theta }$ with Siren	Downsize	NODE-RK	MIRNF [18]
18	LDDMM	$v_{\theta }$ with Siren	Patch	NODE-RK	MIRNF [18]
19	LDDMM	$v_{\theta }$ with Siren	Hybrid	NODE-RK	MIRNF [18]
20	LDDMM	$v_{\theta }$ with (id or K)-Siren	Downsample	NODE-RK	-
21, 22	PDE-LDDMM st. eq.	${\left(D\phi_{t,0}v_{t}\right)}_{\theta }$ and ${\left(D\phi_{0,t}v_{t}\right)}_{\theta }$ with 3D+t Siren	Downsize	Euler, RK	-
23, 24	PDE-LDDMM st. eq.	${\left(D\phi_{t,0}v_{t}\right)}_{\theta }$ and ${\left(D\phi_{0,t}v_{t}\right)}_{\theta }$ with 3D+t Siren	Patch	Euler, RK	-
25, 26	PDE-LDDMM st. eq.	${\left(D\phi_{t,0}v_{t}\right)}_{\theta }$ and ${\left(D\phi_{0,t}v_{t}\right)}_{\theta }$ with 3D+t Siren	Hybrid	Euler, RK	-
27, 28	PDE-LDDMM st. eq.	${\left(D\phi_{t,0}v_{t}\right)}_{\theta }$ and ${\left(D\phi_{0,t}v_{t}\right)}_{\theta }$ with 3D+t (id or K)-Siren	Downsample	Euler, RK	-
29	PDE-LDDMM st. eq.	${\left(D\phi_{t,0}v_{t}\right)}_{\theta }$ and ${\left(D\phi_{0,t}v_{t}\right)}_{\theta }$ with Siren	Downsize	NODE-Euler	-
30	PDE-LDDMM st. eq.	${\left(D\phi_{t,0}v_{t}\right)}_{\theta }$ and ${\left(D\phi_{0,t}v_{t}\right)}_{\theta }$ with Siren	Patch	NODE-Euler	-
31	PDE-LDDMM st. eq.	${\left(D\phi_{t,0}v_{t}\right)}_{\theta }$ and ${\left(D\phi_{0,t}v_{t}\right)}_{\theta }$ with Siren	Hybrid	NODE-Euler	-
32	PDE-LDDMM st. eq.	${\left(D\phi_{t,0}v_{t}\right)}_{\theta }$ and ${\left(D\phi_{0,t}v_{t}\right)}_{\theta }$ with (id or K)-Siren	Downsample	NODE-Euler	NODEO-PDE-LDDMM [21]
33	PDE-LDDMM st. eq.	${\left(D\phi_{t,0}v_{t}\right)}_{\theta }$ and ${\left(D\phi_{0,t}v_{t}\right)}_{\theta }$ with Siren	Downsize	NODE-RK	-
34	PDE-LDDMM st. eq.	${\left(D\phi_{t,0}v_{t}\right)}_{\theta }$ and ${\left(D\phi_{0,t}v_{t}\right)}_{\theta }$ with Siren	Patch	NODE-RK	-
35	PDE-LDDMM st. eq.	${\left(D\phi_{t,0}v_{t}\right)}_{\theta }$ and ${\left(D\phi_{0,t}v_{t}\right)}_{\theta }$ with Siren	Hybrid	NODE-RK	-
36	PDE-LDDMM st. eq.	${\left(D\phi_{t,0}v_{t}\right)}_{\theta }$ and ${\left(D\phi_{0,t}v_{t}\right)}_{\theta }$ with (id or K)-Siren	Downsample	NODE-RK	-
37, 38	PDE-LDDMM def. eq.	${\left(D\phi_{t,0}v_{t}\right)}_{\theta }$ and ${\left(\nabla \cdot \left(\rho_{t}v_{t}\right)\right)}_{\theta }$ with 3D+t Siren	Downsize	Euler, RK	-
39, 40	PDE-LDDMM def. eq.	${\left(D\phi_{t,0}v_{t}\right)}_{\theta }$ and ${\left(\nabla \cdot \left(\rho_{t}v_{t}\right)\right)}_{\theta }$ with 3D+t Siren	Patch	Euler, RK	-
41, 42	PDE-LDDMM def. eq.	${\left(D\phi_{t,0}v_{t}\right)}_{\theta }$ and ${\left(\nabla \cdot \left(\rho_{t}v_{t}\right)\right)}_{\theta }$ with 3D+t Siren	Hybrid	Euler, RK	-
43, 44	PDE-LDDMM def. eq.	${\left(D\phi_{t,0}v_{t}\right)}_{\theta }$ and ${\left(\nabla \cdot \left(\rho_{t}v_{t}\right)\right)}_{\theta }$ with 3D+t (id or K)-Siren	Downsample	Euler, RK	-
45	PDE-LDDMM def. eq.	${\left(D\phi_{t,0}v_{t}\right)}_{\theta }$ and ${\left(\nabla \cdot \left(\rho_{t}v_{t}\right)\right)}_{\theta }$ with Siren	Downsize	NODE-Euler	-
46	PDE-LDDMM def. eq.	${\left(D\phi_{t,0}v_{t}\right)}_{\theta }$ and ${\left(\nabla \cdot \left(\rho_{t}v_{t}\right)\right)}_{\theta }$ with Siren	Patch	NODE-Euler	-
47	PDE-LDDMM def. eq.	${\left(D\phi_{t,0}v_{t}\right)}_{\theta }$ and ${\left(\nabla \cdot \left(\rho_{t}v_{t}\right)\right)}_{\theta }$ with Siren	Hybrid	NODE-Euler	-
48	PDE-LDDMM def. eq.	${\left(D\phi_{t,0}v_{t}\right)}_{\theta }$ and ${\left(\nabla \cdot \left(\rho_{t}v_{t}\right)\right)}_{\theta }$ with (id or K)-Siren	Downsample	NODE-Euler	NODEO-PDE-LDDMM [21]
49	PDE-LDDMM def. eq.	${\left(D\phi_{t,0}v_{t}\right)}_{\theta }$ and ${\left(\nabla \cdot \left(\rho_{t}v_{t}\right)\right)}_{\theta }$ with Siren	Downsize	NODE-RK	-
50	PDE-LDDMM def. eq.	${\left(D\phi_{t,0}v_{t}\right)}_{\theta }$ and ${\left(\nabla \cdot \left(\rho_{t}v_{t}\right)\right)}_{\theta }$ with Siren	Patch	NODE-RK	-
51	PDE-LDDMM def. eq.	${\left(D\phi_{t,0}v_{t}\right)}_{\theta }$ and ${\left(\nabla \cdot \left(\rho_{t}v_{t}\right)\right)}_{\theta }$ with Siren	Hybrid	NODE-RK	-
52	PDE-LDDMM def. eq.	${\left(D\phi_{t,0}v_{t}\right)}_{\theta }$ and ${\left(\nabla \cdot \left(\rho_{t}v_{t}\right)\right)}_{\theta }$ with (id or K)-Siren	Downsample	NODE-RK	-
53	LDDMM	$v_{\theta }$ with (id or K)-CNN	Downsample	NODE-Euler	NODEO-LDDMM [21]
54	PDE-LDDMM st. eq.	${\left(D\phi_{t,0}v_{t}\right)}_{\theta }$ and ${\left(D\phi_{0,t}v_{t}\right)}_{\theta }$ with (id or K)-CNN	Downsample	NODE-Euler	NODEO-PDE-LDDMM [21]
55	PDE-LDDMM def. eq.	${\left(D\phi_{t,0}v_{t}\right)}_{\theta }$ and ${\left(\nabla \cdot \left(\rho_{t}v_{t}\right)\right)}_{\theta }$ with (id or K)-CNN	Downsample	NODE-Euler	NODEO-PDE-LDDMM [21]
56	LDDMM	$v_{\theta }$ with (id or K)-CNN	Downsample	NODE-RK	-
57	PDE-LDDMM st. eq.	${\left(D\phi_{t,0}v_{t}\right)}_{\theta }$ and ${\left(D\phi_{0,t}v_{t}\right)}_{\theta }$ with (id or K)-CNN	Downsample	NODE-RK	-
58	PDE-LDDMM def. eq.	${\left(D\phi_{t,0}v_{t}\right)}_{\theta }$ and ${\left(\nabla \cdot \left(\rho_{t}v_{t}\right)\right)}_{\theta }$ with (id or K)-CNN	Downsample	NODE-RK	-

**Table 3:** NIREP. Evaluation results obtained from the baseline methods. The evaluation metrics include the mean and standard deviation of the Dice similarity coefficient (DSC), the maximum and minimum Jacobian determinants, and the count of non-positive Jacobian determinants. The arrows indicate that high DSC values but non-extreme Jacobian determinant values are preferable. Gray cells indicate, for each family of methods, the one with the highest DSC average. Boldface indicates the method with the highest DSC value. NODEO results were obtained with the parameters selected in [21].

NIREP, Baseline Methods				
Method	DSC (%) ↑	Max (J) ↓	Min (J) ↑	# of $|J_{\phi }|\;\leq \;0$ ↓
Affine	43.56 ± 1.94	-	-	-
VM-GIT 2021	61.35 ± 2.24	12.48 ± 2.17	0.04 ± 0.06	$6\;(6.53\times 10^{-6})$
SyMNet-Diff	61.13 ± 2.24	15.27 ± 2.25	−0.15 ± 0.27	$29\;(3.16\times 10^{-5})$
LapIRN-Diff	60.44 ± 1.23	6.66 ± 0.78	0.16 ± 0.03	0
TransMorph-BSpl IXI	58.95 ± 1.69	10.63 ± 1.90	0.06 ± 0.02	0
ANTS	60.24 ± 1.35	4.11 ± 0.39	0.24 ± 0.03	0
StLDDMM	62.10 ± 1.54	15.09 ± 4.46	0.02 ± 0.22	$39\;(4.25\times 10^{-5})$
PDE-LDDMM_st eq_	60.11 ± 2.81	7.41 ± 3.56	0.01 ± 0.01	0
PDE-LDDMM_def eq_	60.92 ± 1.88	15.59 ± 5.89	0.05 ± 0.03	0
NODEO-CVPR22	63.74 ± 0.90	11.02 ± 2.69	−0.19 ± 0.12	$776\;(8.45\times 10^{-4})$
NODEO-LDDMM	62.83 ± 1.25	8.93 ± 1.50	−0.06 ± 0.12	$242\;(2.63\times 10^{-4})$
NODEO-PDE-LDDMM_st eq_	62.64 ± 1.32	8.55 ± 1.41	−0.01 ± 0.07	$100\;(1.09\times 10^{-4})$
NODEO-PDE-LDDMM_def eq_	62.75 ± 1.46	9.63 ± 3.07	−0.06 ± 0.08	$236\;(2.57\times 10^{-4})$

**Table 4:** NIREP. Evaluation results obtained from Siren-LDDMM, Siren-PDE-LDDMM, NODEO-LDDMM, and NODEO-PDE-LDDMM. Comparison of the performance of the methods across different solver configurations (SS, Euler, RK, and stationary NODEs) and downsize, hybrid, and downsample sampling strategies. The evaluation metrics include the mean and standard deviation of the Dice similarity coefficient (DSC), the maximum and minimum Jacobian determinants, and the count of non-positive Jacobian determinants. The arrows indicate that high DSC values but non-extreme Jacobian determinant values are preferable. The mean computation time is included in the table for a complementary assessment. Boldface indicates, for each variant, the one with the highest DSC average. The grey cells highlight the solvers with the highest DSC average within each family of methods. Red cells highlight methods with a number of negative Jacobians above one thousand.

NIREP, Siren-LDDMM and Siren-PDE-LDMM, K = id, 300 its					
Method	DSC (%) ↑	Max (J) ↓	Min (J) ↑	# of $|J_{\phi }|\;\leq \;0$ ↓	${\mathrm{Time}}_{\mathrm{GPU}}$ (s) ↓
LDDMM, SS, Downsize	60.78 ± 1.44	10.80 ± 2.63	−0.68 ± 0.55	$1552\;(1.69\times 10^{-3})$	79.75
LDDMM, SS, Hybrid	59.78 ± 1.67	11.78 ± 2.96	−0.74 ± 0.49	$1688\;(1.84\times 10^{-3})$	246.01
LDDMM, SS, Downsample	61.50 ± 2.12	13.27 ± 2.89	−0.06 ± 0.18	$53\;(5.77\times 10^{-5})$	343.25
LDDMM, Euler, Downsize	61.59 ± 1.25	15.49 ± 3.62	−1.55 ± 0.65	$5194\;(5.65\times 10^{-3})$	63.84
LDDMM, Euler, Hybrid	61.48 ± 1.25	16.09 ± 4.71	−1.87 ± 1.33	$5436\;(5.92\times 10^{-3})$	238.58
LDDMM, Euler, Downsample	61.73 ± 1.52	12.52 ± 2.74	0.08 ± 0.13	$14\;(1.52\times 10^{-5})$	345.06
LDDMM, St. NODE, Euler, Downsize	62.11 ± 1.38	28.00 ± 16.24	−1.51 ± 2.87	$399\;(4.34\times 10^{-4})$	300.11
LDDMM, St. NODE, Euler, Hybrid	61.91 ± 1.39	25.25 ± 10.82	−1.65 ± 2.50	$628\;(6.84\times 10^{-4})$	579.86
LDDMM, St. NODE, Euler, Downsample	62.72 ± 1.50	15.82 ± 5.92	−0.07 ± 0.13	$46\;(5.01\times 10^{-5})$	445.31
LDDMM, RK, Downsize	61.59 ± 1.34	14.85 ± 4.18	−1.51 ± 0.88	$5162\;(5.62\times 10^{-3})$	64.28
LDDMM, RK, Hybrid	61.23 ± 1.28	15.34 ± 4.84	−1.17 ± 0.88	$2829\;(3.08\times 10^{-3})$	238.51
LDDMM, RK, Downsample	61.67 ± 1.62	12.47 ± 4.13	0.15 ± 0.08	$1\;(1.09\times 10^{-6})$	401.56
LDDMM, St. NODE, RK, Downsize	62.32 ± 1.37	33.73 ± 23.42	−1.52 ± 0.91	$357\;(3.89\times 10^{-4})$	682.09
LDDMM, St. NODE, RK, Hybrid	62.15 ± 1.29	30.60 ± 9.90	−1.63 ± 1.33	$318\;(3.46\times 10^{-4})$	1167
LDDMM, St. NODE, RK, Downsample	62.75 ± 1.43	15.76 ± 6.33	−0.10 ± 0.13	$76\;(8.27\times 10^{-5})$	592.77
PDE-LDDMM_st eq_, Euler, Downsize	61.47 ± 1.33	16.05 ± 3.80	−1.19 ± 0.35	$5351\;(5.83\times 10^{-3})$	84.62
PDE-LDDMM_st eq_, Euler, Hybrid	61.36 ± 1.25	17.78 ± 5.42	−1.45 ± 0.62	$5103\;(5.56\times 10^{-3})$	119.55
PDE-LDDMM_st eq_, Euler, Downsample	61.76 ± 1.48	13.67 ± 4.16	0.09 ± 0.19	$19\;(2.07\times 10^{-5})$	387.19
PDE-LDDMM_st eq_, St. NODE, Euler, Downsize	62.03 ± 1.39	26.68 ± 10.91	−1.46 ± 2.21	$393\;(4.28\times 10^{-4})$	365.94
PDE-LDDMM_st eq_, St. NODE, Euler, Hybrid	62.02 ± 1.29	26.27 ± 9.57	−1.13 ± 1.19	$459\;(5.00\times 10^{-4})$	654.73
PDE-LDDMM_st eq_, St. NODE, Euler, Downsample	62.46 ± 1.40	14.41 ± 2.47	0.00 ± 0.13	$15\;(1.63\times 10^{-5})$	444.95
PDE-LDDMM_st eq_, RK, Downsize	61.49 ± 1.41	14.54 ± 2.85	−1.28 ± 0.55	$4727\;(5.15\times 10^{-3})$	85.69
PDE-LDDMM_st eq_, RK, Hybrid	61.36 ± 1.34	16.72 ± 5.89	−1.61 ± 0.81	$5173\;(5.63\times 10^{-3})$	121.79
PDE-LDDMM_st eq_, RK, Downsample	61.81 ± 1.46	13.37 ± 4.03	0.05 ± 0.15	$19\;(2.07\times 10^{-5})$	418.05
PDE-LDDMM_st eq_, St. NODE, RK, Downsize	62.13 ± 1.35	38.58 ± 15.74	−2.50 ± 2.89	$407\;(4.43\times 10^{-4})$	1118.20
PDE-LDDMM_st eq_, St. NODE, RK, Hybrid	62.01 ± 1.28	35.25 ± 18.62	−1.47 ± 0.85	$391\;(4.26\times 10^{-4})$	1832.58
PDE-LDDMM_st eq_, St. NODE, RK, Downsample	62.39 ± 1.43	15.81 ± 4.84	−0.02 ± 0.16	$24\;(2.61\times 10^{-5})$	658.58
PDE-LDDMM_def eq_, Euler, Downsize	61.46 ± 1.26	16.23 ± 4.02	−1.76 ± 1.17	$5319\;(5.79\times 10^{-3})$	83.65
PDE-LDDMM_def eq_, Euler, Hybrid	61.34 ± 1.34	16.48 ± 3.20	−1.74 ± 0.81	$5656\;(6.16\times 10^{-3})$	115.85
PDE-LDDMM_def eq_, Euler, Downsample	61.69 ± 1.59	13.15 ± 3.07	0.02 ± 0.15	$21\;(2.29\times 10^{-5}\%)$	374.68
PDE-LDDMM_def eq_, St. NODE, Euler, Downsize	62.12 ± 1.32	27.15 ± 9.78	−1.37 ± 2.12	$302\;(3.29\times 10^{-4})$	363.33
PDE-LDDMM_def eq_, St. NODE, Euler, Hybrid	62.09 ± 1.25	31.86 ± 28.40	−1.97 ± 2.70	$1034\;(1.13\times 10^{-3})$	591.42
PDE-LDDMM_def eq_, St. NODE, Euler, Downsample	62.43 ± 1.43	15.88 ± 3.64	−0.03 ± 0.14	$26\;(2.83\times 10^{-5})$	414.57
PDE-LDDMM_def eq_, RK, Downsize	61.48 ± 1.38	15.99 ± 3.83	−1.42 ± 0.53	$4921\;(5.36\times 10^{-3})$	84.46
PDE-LDDMM_def eq_, RK, Hybrid	61.18 ± 1.47	14.39 ± 3.83	−1.10 ± 0.48	$2696\;(2.94\times 10^{-3})$	117.93
PDE-LDDMM_def eq_, RK, Downsample	61.50 ± 1.75	13.51 ± 4.04	0.09 ± 0.13	$12\;(1.31\times 10^{-5})$	404.80
PDE-LDDMM_def eq_, St. NODE, RK, Downsize	62.24 ± 1.22	28.96 ± 10.65	−2.10 ± 1.25	$481\;(5.24\times 10^{-4})$	1115.11
PDE-LDDMM_def eq_, St. NODE, RK, Hybrid	62.27 ± 1.36	36.42 ± 18.28	−2.51 ± 2.26	$1161\;(1.26\times 10^{-3})$	1661.77
PDE-LDDMM_def eq_, St. NODE, RK, Downsample	62.36 ± 1.48	16.26 ± 2.82	−0.11 ± 0.27	$55\;(5.99\times 10^{-5})$	621.19
**NIREP, NODEO-LDDMM and NODEO-PDE-LDMM,** $K\;=\;{\;(L^{†}L)}^{-1}$**, 300 its**					
**Method**	**DSC (%) ↑**	**Max (J) ↓**	**Min (J) ↑**	**# of** $|J_{\phi }|\;\leq \;0$ **↓**	${\mathrm{time}}_{\mathrm{GPU}}$ **(s) ↓**
LDDMM, St. NODE, Euler, Downsample	63.13 ± 1.69	7.27 ± 2.05	0.25 ± 0.18	0	58.97
LDDMM, St. NODE, RK, Downsample	63.20 ± 1.63	7.55 ± 2.28	0.22 ± 0.19	0	79.26
PDE-LDDMM_st eq_, St. NODE, Euler, Downsample	62.94 ± 1.57	7.47 ± 0.75	0.19 ± 0.08	$1\;(1.09\times 10^{-6})$	161.92
PDE-LDDMM_st eq_, St. NODE, RK, Downsample	62.21 ± 2.71	6.79 ± 1.36	0.23 ± 0.09	0	180.26
PDE-LDDMM_def eq_, St. NODE, Euler, Downsample	62.71 ± 1.56	7.48 ± 0.98	0.24 ± 0.06	0	110.85
PDE-LDDMM_def eq_, St. NODE, RK, Downsample	62.34 ± 1.70	7.09 ± 1.23	0.26 ± 0.07	0	127.12

**Table 5:** NIREP. Siren-LDDMM and Siren-PDE-LDDMM with patch-based sampling strategy from scratch. Patch size of 64 × 64 × 64. Comparison of the performance of the methods across different solver configurations (Scaling and Squaring, Euler, and RK). The evaluation metrics include the mean and standard deviation of the Dice similarity coefficient (DSC), the maximum and minimum Jacobian determinants, and the count of non-positive Jacobian determinants. The table also shows the order of the computation time. The experiments with NODE solvers are excluded because of the extremely high computational demands of the resulting variants.

NIREP, Siren-LDDMM and Siren-PDE-LDDMM, K = id, 300 its					
Method	DSC (%) ↑	Max (J) ↓	Min (J) ↑	# of $|J_{\phi }|\;\leq \;0$ ↓	${\mathrm{Time}}_{\mathrm{GPU}}$ (s) ↓
LDDMM, SS, Patch	55.55 ± 1.34	3.87 ± 0.56	0.25 ± 0.03	0	O(10,000)
LDDMM, Euler, Patch	57.54 ± 1.22	5.45 ± 0.88	0.24 ± 0.08	0	O(10,000)
LDDMM, RK, Patch	57.32 ± 1.37	5.11 ± 0.61	0.21 ± 0.05	0	O(10,000)
PDE-LDDMM_st eq_, Euler, Patch	58.87 ± 1.22	7.03 ± 1.93	0.20 ± 0.08	0	O(1000)
PDE-LDDMM_st eq_, RK, Patch	58.88 ± 1.34	7.14 ± 1.14	0.15 ± 0.11	0	O(1000)
PDE-LDDMM_def eq_, Euler, Patch	59.01 ± 1.34	6.85 ± 1.64	0.20 ± 0.07	0	O(1000)
PDE-LDDMM_def eq_, RK, Patch	58.34 ± 1.20	6.64 ± 1.18	0.19 ± 0.13	5	O(1000)

**Table 6:** NIREP. Evaluation results obtained from Siren-LDDMM and Siren-PDE-LDDMM with hybrid sampling and patch sizes of 64 × 64 × 64. Boldface indicates the one with the highest DSC average. The gray cells highlight the solvers with the highest DSC average within each family of methods. Red cells highlight methods with a number of negative Jacobians above one thousand.

NIREP, Siren-LDDMM and Siren-PDE-LDMM, K = id, 300 its					
Method	DSC (%) ↑	Max (J) ↓	Min (J) ↑	# of $|J_{\phi }|\;\leq \;0$ ↓	${\mathrm{Time}}_{\mathrm{GPU}}$ (s) ↓
LDDMM, SS, Hybrid	60.70 ± 1.75	10.27 ± 2.18	−0.97 ± 0.48	$935\;(1.02\times 10^{-3})$	242.48
LDDMM, Euler, Hybrid	62.21 ± 1.31	17.44 ± 4.97	−1.95 ± 0.76	$3210\;(3.49\times 10^{-3})$	315.81
LDDMM, RK, Hybrid	62.14 ± 1.51	15.14 ± 4.04	−1.16 ± 0.56	$1505\;(1.64\times 10^{-3})$	339.26
PDE-LDDMM_st eq_, Euler, Hybrid	62.28 ± 1.32	16.59 ± 3.94	−1.60 ± 0.78	$3103\;(3.38\times 10^{-3})$	276.32
PDE-LDDMM_st eq_, RK, Hybrid	62.15 ± 1.48	16.82 ± 3.51	−1.91 ± 0.75	$2939\;(3.20\times 10^{-3})$	279.30
PDE-LDDMM_def eq_, Euler, Hybrid	62.16 ± 1.36	16.04 ± 2.99	−1.62 ± 0.49	$2922\;(3.18\times 10^{-3})$	191.00
PDE-LDDMM_def eq_, RK, Hybrid	61.94 ± 1.69	13.01 ± 2.20	−1.10 ± 0.40	$1517\;(1.65\times 10^{-3})$	193.09

**Table 7:** NIREP. Siren-LDDMM, Siren-PDE-LDDMM, NODEO-LDDMM, and NODEO-PDE-LDDMM. Comparison of the performance of the analogs of the methods in Table 4, swapping the kernel layer between ${\;(L^{†}L)}^{-1}$ and the identity. The evaluation metrics include the mean and standard deviation of the Dice similarity coefficient (DSC), the maximum and minimum Jacobian determinants, and the count of non-positive Jacobian determinants.

NIREP, Siren-LDDMM and Siren-PDE-LDDMM, $K\;=\;{\left(L^{†}L\right)}^{-1}$**, 300 its**				
Method	DSC (%) ↑	Max (J) ↓	Min (J) ↑	# of $|J_{\phi }|\;\leq \;0$ ↓
LDDMM, SS, Downsample	56.26 ± 1.38	6.25 ± 0.68	0.45 ± 0.01	0
LDDMM, Euler, Downsample	56.11 ± 1.45	6.09 ± 0.87	0.45 ± 0.01	0
LDDMM, St. NODE, Euler, Downsample	56.62 ± 1.32	5.39 ± 0.60	0.45 ± 0.01	0
LDDMM, RK, Downsample	55.87 ± 1.55	5.91 ± 0.93	0.45 ± 0.01	0
LDDMM, St. NODE, RK, Downsample	56.55 ± 1.31	5.40 ± 0.55	0.46 ± 0.01	0
PDE-LDDMM_st eq_, Euler, Downsample	56.06 ± 1.35	6.62 ± 0.96	0.44 ± 0.02	0
PDE-LDDMM_st eq_, St. NODE Euler, Downsample	56.14 ± 1.25	6.24 ± 0.83	0.45 ± 0.01	0
PDE-LDDMM_st eq_, RK, Downsample	55.91 ± 1.57	6.96 ± 1.21	0.45 ± 0.01	0
PDE-LDDMM_st eq_, St. NODE RK, Downsample	56.28 ± 1.30	6.40 ± 0.87	0.45 ± 0.01	0
PDE-LDDMM_def eq_, Euler, Downsample	56.14 ± 1.44	6.60 ± 0.96	0.45 ± 0.02	0
PDE-LDDMM_def eq_, St. NODE Euler, Downsample	56.19 ± 1.07	6.25 ± 0.82	0.45 ± 0.01	0
PDE-LDDMM_def eq_, RK, Downsample	55.85 ± 1.58	6.45 ± 0.98	0.45 ± 0.01	0
PDE-LDDMM_def eq_,, St. NODE, RK, Downsample	56.12 ± 1.43	6.36 ± 0.90	0.44 ± 0.01	0
**NIREP, NODEO-LDDMM and NODEO-PDE-LDDMM,** K = id **, 300 its**				
**Method**	**DSC (%) ↑**	**Max (J) ↓**	**Min (J) ↑**	**# of** $|J_{\phi }|\;\leq \;0$ **↓**
LDDMM, St. NODE, Euler, Downsample	52.70 ± 2.55	5.84 ± 0.52	−0.09 ± 0.08	$35\;(3.81\times 10^{-5})$
LDDMM, St. NODE, RK, Downsample	51.87 ± 2.86	5.59 ± 0.56	−0.06 ± 0.08	$23\;(2.50\times 10^{-5})$
PDE-LDDMM_st eq_,, St. NODE Euler, Downsample	65.08 ± 1.79	19.47 ± 5.23	−1.62 ± 0.71	15,187 $\;(1.65\times 10^{-2})$
PDE-LDDMM_st eq_, St. NODE RK, Downsample	65.02 ± 1.79	18.56 ± 4.01	−1.60 ± 0.66	13,691 $\;(1.49\times 10^{-2})$
PDE-LDDMM_def eq_, St. NODE Euler, Downsample	59.01 ± 7.60	11.82 ± 5.21	−0.62 ± 0.54	$5412\;(5.89\times 10^{-3})$
PDE-LDDMM_def eq_, St. NODE RK, Downsample	60.47 ± 6.18	12.94 ± 5.46	−0.99 ± 0.99	$2178\;(2.37\times 10^{-3})$

**Table 8:** OASIS. Evaluation results obtained from the baseline methods. The evaluation metrics include the mean and standard deviation of the Dice similarity coefficient (DSC), the maximum and minimum Jacobian determinants, and the count of non-positive Jacobian determinants. The arrows indicate that high DSC values but non-extreme Jacobian determinant values are preferable. Gray cells indicate, for each family of methods, the one with the highest DSC average. Boldface indicates the method with the highest DSC value. NODEO results were obtained with the parameters selected in [21]. Red cells highlight methods with a number of negative Jacobians above one thousand. The severity of folding artifacts is indicated with darker red.

OASIS, Baseline Methods				
Method	DSC (%) ↑	Max (J) ↓	Min (J) ↑	# of $|J_{\phi }|\;\leq \;0$ ↓
Affine	58.61 ± 5.09	-	-	-
VM-GIT 2021	77.66 ± 2.93	23.69 ± 9.58	0.00 ± 0.13	2 $\;(9.16\times 10^{-7})$
SyMNet-Diff	78.71 ± 2.57	21.74 ± 7.15	−0.10 ± 0.20	53 $\;(2.43\times 10^{-5})$
LapIRN-Diff	78.45 ± 2.23	4.52 ± 1.12	0.23 ± 0.05	0
TransMorph-BSpl IXI	77.78 ± 1.49	13.13 ± 2.87	−0.05 ± 0.10	199 $\;(9.12\times 10^{-5})$
ANTS	79.21 ± 1.89	4.65 ± 1.07	0.22 ± 0.04	0
StLDDMM	78.49 ± 1.77	16.55 ± 17.86	−0.35 ± 0.83	3480 $\;(1.59\times 10^{-3})$
PDE-LDDMM_st eq_	75.98 ± 2.67	13.82 ± 15.70	0.01 ± 0.01	0
PDE-LDDMM_def eq_	76.30 ± 2.36	18.25 ± 17.67	0.09 ± 0.04	0
NODEO-CVPR22	79.96 ± 1.52	18.47 ± 6.24	−0.46 ± 0.21	16,927 ($7.75\times 10^{-3}$)
NODEO-LDDMM	79.43 ± 1.59	14.63 ± 7.67	−0.22 ± 0.14	5944 ($2.72\times 10^{-3}$)
NODEO-PDE-LDDMM_st eq_	79.49 ± 1.57	14.56 ± 7.52	−0.21 ± 0.12	6716 ($3.08\times 10^{-3}$)
NODEO-PDE-LDDMM_def eq_	79.47 ± 1.52	14.38 ± 6.34	−0.24 ± 0.12	7489 ($3.43\times 10^{-3}$)

**Table 9:** OASIS. Evaluation results obtained from Siren-LDDMM, Siren-PDE-LDDMM, NODEO-LDDMM, and NODEO-PDE-LDDMM. Comparison of the performance of the methods across different solver configurations (SS, Euler, RK, and stationary NODEs) and downsize, hybrid, and downsample sampling strategies. The evaluation metrics include the mean and standard deviation of the Dice similarity coefficient (DSC), the maximum and minimum Jacobian determinants, and the count of non-positive Jacobian determinants. The arrows indicate that high DSC values but non-extreme Jacobian determinant values are preferable. The mean computation time is included in the table for a complementary assessment. Boldface indicates, for each variant, the one with the highest DSC average. The grey cells highlight the solvers with the highest DSC average within each family of methods. Red cells highlight methods with a number of negative Jacobians above one thousand. The severity of folding artifacts is indicated with darker red.

OASIS, Siren-LDDMM and Siren-PDE-LDDMM, K = id, 300 its				
Method	DSC (%) ↑	Max (J) ↓	Min (J) ↑	# of $|J_{\phi }|\;\leq \;0$ ↓
LDDMM, SS, Downsize	76.07 ± 3.99	18.48 ± 6.78	−2.17 ± 0.87	33,530$\;(1.54\times 10^{-2})$
LDDMM, SS, Hybrid	75.66 ± 4.32	16.99 ± 7.40	−1.72 ± 0.98	11,146$\;(5.11\times 10^{-3})$
LDDMM, SS, Downsample	77.70 ± 4.04	16.85 ± 6.10	−0.03 ± 0.14	$146\;(6.69\times 10^{-5})$
LDDMM, Euler, Downsize	76.49 ± 3.70	21.05 ± 7.13	−2.38 ± 1.20	28,562$\;(1.31\times 10^{-2})$
LDDMM, Euler, Hybrid	77.20 ± 4.22	27.44 ± 12.24	−2.80 ± 1.44	30,704$\;(1.41\times 10^{-2})$
LDDMM, Euler, Downsample	77.40 ± 4.18	15.07 ± 5.57	0.13 ± 0.16	$13\;(5.96\times 10^{-6})$
LDDMM, St. NODE, Euler, Downsize	76.89 ± 3.82	49.77 ± 26.94	−1.99 ± 1.37	$2925\;(1.34\times 10^{-3})$
LDDMM, St. NODE, Euler, Downsample	78.27 ± 3.75	20.42 ± 7.75	−0.10 ± 0.21	$158\;(7.24\times 10^{-5})$
LDDMM, RK, Downsize	76.70 ± 3.80	25.27 ± 12.47	−3.39 ± 1.56	68,716$\;(3.15\times 10^{-2})$
LDDMM, RK Hybrid	77.33 ± 4.25	24.57 ± 9.88	−1.79 ± 0.72	17,540$\;(8.04\times 10^{-3})$
LDDMM, RK, Downsample	77.43 ± 4.05	15.91 ± 5.64	0.12 ± 0.16	$28\;(1.28\times 10^{-5})$
LDDMM, St. NODE, RK, Downsize	76.90 ± 3.70	62.20 ± 27.79	−4.10 ± 4.56	$2723\;(1.25\times 10^{-3})$
LDDMM, St. NODE, RK, Downsample	78.27 ± 3.76	20.42 ± 7.46	−0.16 ± 0.29	$182\;(8.34\times 10^{-5})$
PDE-LDDMM_st eq_, Euler, Downsize	76.44 ± 3.73	23.90 ± 10.77	−1.98 ± 1.14	27,173$\;(1.24\times 10^{-2})$
PDE-LDDMM_st eq_, Euler, Hybrid	76.85 ± 4.20	26.00 ± 11.46	−2.77 ± 1.86	31,403$\;(1.44\times 10^{-2})$
PDE-LDDMM_st eq_, Euler, Downsample	77.21 ± 4.30	16.79 ± 7.17	0.06 ± 0.23	$163\;(7.47\times 10^{-5})$
PDE-LDDMM_st eq_, St. NODE, Euler, Downsize	75.80 ± 3.67	48.71 ± 26.41	−1.55 ± 2.08	$2060\;(9.44\times 10^{-4})$
PDE-LDDMM_st eq_, St. NODE, Euler, Downsample	77.82 ± 3.98	24.03 ± 8.30	−0.13 ± 0.24	$182\;(8.34\times 10^{-5})$
PDE-LDDMM_st eq_, RK, Downsize	76.43 ± 3.78	23.10 ± 12.16	−1.98 ± 0.86	29,590$\;(1.36\times 10^{-2})$
PDE-LDDMM_st eq_, RK, Hybrid	76.77 ± 4.24	27.95 ± 11.80	−2.99 ± 1.77	30,606$\;(1.40\times 10^{-2})$
PDE-LDDMM_st eq_, RK, Downsample	77.05 ± 4.20	16.57 ± 6.13	0.13 ± 0.18	$34\;(1.56\times 10^{-5})$
PDE-LDDMM_st eq_, St. NODE, RK, Downsize	75.44 ± 3.72	53.92 ± 22.33	−3.80 ± 6.24	$2313\;(1.06\times 10^{-3})$
PDE-LDDMM_st eq_, St. NODE, RK, Downsample	77.77 ± 3.95	23.39 ± 8.40	−0.14 ± 0.24	$272\;(1.25\times 10^{-4})$
PDE-LDDMM_def eq_, Euler, Downsize	76.40 ± 3.84	22.94 ± 7.11	−2.12 ± 1.19	29,665$\;(1.36\times 10^{-2})$
PDE-LDDMM_def eq_, Euler, Hybrid	76.75 ± 4.27	26.11 ± 11.24	−2.47 ± 1.30	29,559$\;(1.35\times 10^{-2})$
PDE-LDDMM_def eq_, Euler, Downsample	77.18 ± 4.18	17.06 ± 7.70	0.12 ± 0.17	$46\;(2.11\times 10^{-5})$
PDE-LDDMM_def eq_, St. NODE, Euler, Downsize	76.63 ± 3.76	56.69 ± 35.36	−3.23 ± 2.55	$2954\;(1.35\times 10^{-3})$
PDE-LDDMM_def eq_, St. NODE, Euler, Downsample	77.88 ± 3.88	23.08 ± 8.23	−0.14 ± 0.22	$228\;(1.04\times 10^{-4})$
PDE-LDDMM_def eq_, RK, Downsize	76.41 ± 3.79	21.27 ± 10.41	−2.31 ± 1.17	28,576$\;(1.31\times 10^{-2})$
PDE-LDDMM_def eq_, RK, Hybrid	77.32 ± 4.45	25.56 ± 12.25	−2.01 ± 0.92	18,167$\;(8.32\times 10^{-3})$
PDE-LDDMM_def eq_, RK, Downsample	77.01 ± 4.16	16.19 ± 7.82	0.12 ± 0.17	$48\;(2.20\times 10^{-5})$
PDE-LDDMM_def eq_, St. NODE, RK, Downsize	76.76 ± 3.80	59.69 ± 27.49	−4.70 ± 3.20	$3845\;(1.76\times 10^{-3})$
PDE-LDDMM_def eq_>, St. NODE, RK, Downsample	77.76 ± 3.90	24.34 ± 9.19	−0.20 ± 0.23	$303\;(1.39\times 10^{-4})$
**OASIS, NODEO-LDDMM and NODEO-PDE-LDDMM,** $K={\;(L^{†}L)}^{-1}$ **, 300 its**				
**Method**	**DSC (%) ↑**	**Max (J) ↓**	**Min (J) ↑**	**# of** $|J_{\phi }|\;\leq \;0$ **↓**
LDDMM, St. NODE, Euler, Downsample	77.45 ± 4.77	12.53 ± 7.00	0.16 ± 0.19	$35\;(1.60\times 10^{-5})$
LDDMM, St. NODE, RK, Downsample	77.41 ± 5.64	12.64 ± 6.68	0.13 ± 0.19	$114\;(5.22\times 10^{-5})$
PDE-LDDMM_st eq_, St. NODE, Euler, Downsample	77.11 ± 5.07	12.18 ± 6.97	0.17 ± 0.19	$28\;(1.28\times 10^{-5})$
PDE-LDDMM_st eq_, St. NODE, RK, Downsample	77.75 ± 4.04	11.11 ± 4.71	0.19 ± 0.17	$23\;(1.05\times 10^{-5})$
PDE-LDDMM_def eq_, St. NODE, Euler, Downsample	77.35 ± 4.43	10.04 ± 4.15	0.20 ± 0.18	$55\;(2.52\times 10^{-5})$
PDE-LDDMM_def eq_, St. NODE, RK, Downsample	77.57 ± 4.32	10.82 ± 4.64	0.19 ± 0.18	$29\;(1.33\times 10^{-5})$

## Data Availability

The NIREP dataset is publicly available through the repository maintained by Andreas Mang https://github.com/andreasmang/nirep (accessed on 3 August 2026). Additional information regarding the Non-rigid Image Registration Evaluation Project (NIREP) can be found at https://www.nitrc.org/projects/nirep/ (accessed on 3 August 2026). The OASIS Learn2Reg dataset is publicly available through the Learn2Reg challenge website https://learn2reg.grand-challenge.org/Learn2Reg2021 (accessed on 3 August 2026). The main experimental results generated in this work will be made publicly available through a public data repository upon acceptance of the manuscript. The source code used in this study, together with the documentation required to reproduce the main experiments, will be made publicly available upon acceptance of the manuscript at https://github.com/mhglddmm/INRvsNODE_LDDMM (accessed on 3 August 2026).

## Supplementary Materials

The following supporting information can be downloaded at https://www.mdpi.com/article/10.3390/jimaging12080384/s1. Table S1: NIREP. Evaluation results obtained from Siren-LDDMM, Siren-PDE-LDDMM, NODEO-LDDMM, and NODEO-PDE-LDDMM. 100 its; Table S2: NIREP. Evaluation results obtained from Siren-LDDMM, Siren-PDE-LDDMM, NODEO-LDDMM, and NODEO-PDE-LDDMM. 150 its; Table S3: NIREP. Maximum VRAM memory usage achieved by the registration methods from Table 4; Table S4: NIREP. Computation time and maximum VRAM memory usage achieved by the registration methods with hybrid-based sampling and NODE-based solvers; Figure S1: Results of the pairwise right-tailed Wilcoxon rank-sum tests to assess the statistical significance of the differences among the methods; Figure S2: NIREP. Baseline methods. Sagittal view of the displacement fields in a representative experiment; Figure S3: NIREP. Baseline methods. Sagittal view of the transformation grids in a representative experiment; Figure S4: NIREP. Sagittal view of the differences after registration in a representative experiment. Siren and NODEO methods with RK and NODE-RK solvers; Figure S5: NIREP. Sagittal view of the displacement fields in a representative experiment. Siren and NODEO methods with RK and NODE-RK solvers; Figure S6: NIREP. Sagittal view of the transformation grids in a representative experiment. Siren and NODEO methods with RK and NODE-RK solvers; Figure S7: OASIS. Sagittal view of the differences after registration in a representative experiment. Siren and NODEO methods with RK and NODE-RK solvers; Figure S8: OASIS. Sagittal view of the displacement fields in a representative experiment. Siren and NODEO methods with RK and NODE-RK solvers: Figure S9: OASIS. Sagittal view of the transformation grids in a representative experiment. Siren and NODEO methods with RK and NODE-RK solvers.

## Appendix A. Algorithms

Algorithm A1 gathers the steps for a gradient descent version of INR-LDDMM. The solution of the transport equation is approached using SS, Euler, or RK solvers. In the case of NODE methods, the right-hand side of the transport equation is computed from the NODE neural representation. The loss gradient is computed using automatic differentiation and used to perform backpropagation and update the neural representation of $-v_{t}^{\theta }\left(\phi_{t,0}\right)$. Algorithms A2 and A3 gather the steps for both variants of our INR-PDE-LDDMM methods. Using the algorithms of the INR-LDDMM variants as a backbone, the algorithms compute the ingredients needed in the computation of $v_{t}$, and backpropagation leads to the update of the implicitly represented variables involved in both approaches.

**Algorithm A1** INR-LDDMM
**Data:** $I_{0}$, $I_{1}$, ${\left(v_{t}^{\theta }\right)}^{0}$, *L*, *K*, σ, $n_{its}$. **Results:** $-v_{t}^{\theta }\left(\phi_{t,0}\right)$, arg min of Equation (14), $\phi_{t,0}$, solution of Equation (7). Build the INR network. Initialize randomly $-v_{t}^{\theta }\left(\phi_{t,0}\right)$. **for** n←0 **to** $n_{its}$ **do** (1) Sample the domain according to the variant strategy. (2) Compute $\phi_{t,0}^{n+1}$ from Equation (7) according to the variant solver. (3) Compute $-v_{t}\left(\phi_{t,0}\right)$ from $-v_{t}^{\theta }\left(\phi_{t,0}\right)$. (4) Compute $I_{0}\circ \phi_{t,0}$ from $I_{0}$ and $\phi_{t,0}^{n+1}$ using a spatial transformer. (5) Compute the image similarity, the regularization losses, and the loss function. (6) Perform backward propagation on $-v_{t}^{\theta }\left(\phi_{t,0}\right)$. (7) Update ${\left(-v_{t}^{\theta }\left(\phi_{t,0}\right)\right)}^{n+1}$. **end** Select the best-performing $-v_{t}^{\theta }\left(\phi_{t,0}\right)$ according to the stopping strategy.

**Algorithm A2** INR-PDE-LDDMM_st eq_
**Data:** $I_{0}$, $I_{1}$, ${\left({\left(D\phi_{t,0}\cdot v_{t}\right)}^{\theta }\right)}^{0}$, ${\left({\left(D\phi_{0,t}\cdot v_{t}\right)}^{\theta }\right)}^{0}$, *L*, *K*, σ, $n_{its}$. **Results:** $v_{t}$, arg min of Equation (14), $\phi_{t,0}$ and $\phi_{0,t}$, solutions of Equations (7) and (8). Build the INR networks. Initialize randomly ${\left(D\phi_{t,0}\cdot v_{t}\right)}^{\theta }$ and ${\left(D\phi_{0,t}\cdot v_{t}\right)}^{\theta }$. **for** n←0 **to** $n_{its}$ **do** (1) Sample the domain according to the variant strategy. (2) Compute $\phi_{t,0}^{n+1}$ and $\phi_{0,t}^{n+1}$ through the variant solvers. (3) Compute $m\left(t\right)=I_{0}\circ \phi_{t,0}$ using a spatial transformer. (4) Compute $\lambda \left(t\right)=J_{t}\lambda_{1}\circ \phi_{0,t}$ using a spatial transformer. (5) Compute v(t) from Equation (10). (6) Compute the image similarity, the regularization losses, and the loss function. (7) Perform backward propagation on ${\left(D\phi_{t,0}\cdot v_{t}\right)}^{\theta }$ and ${\left(D\phi_{0,t}\cdot v_{t}\right)}^{\theta }$. (8) Update ${\left({\left(D\phi_{t,0}\cdot v_{t}\right)}^{\theta }\right)}^{n+1}$ and ${\left({\left(D\phi_{0,t}\cdot v_{t}\right)}^{\theta }\right)}^{n+1}$. **end** Select the best-performing ${\left({\left(D\phi_{t,0}\cdot v_{t}\right)}^{\theta }\right)}^{n+1}$ and ${\left({\left(D\phi_{0,t}\cdot v_{t}\right)}^{\theta }\right)}^{n+1}$ according to the stopping strategy.

**Algorithm A3** INR-PDE-LDDMM_def eq_
**Data:** $I_{0}$, $I_{1}$, ${\left({\left(D\phi_{t,0}\cdot v_{t}\right)}^{\theta }\right)}^{0}$, ${\left({\left(\nabla \cdot \left(\rho_{t}\cdot v_{t}\right)\right)}^{\theta }\right)}^{0}$, *L*, *K*, σ, $n_{its}$. **Results:** $v_{t}$, arg min of Equation (14) and $\phi_{t,0}$ solution of Equation (7). Build the INR networks. Initialize randomly ${\left(D\phi_{t,0}\cdot v_{t}\right)}^{\theta }$ and ${\left(\nabla \cdot \left(\rho_{t}\cdot v_{t}\right)\right)}^{\theta }$. **for** n←0 **to** $n_{its}$ **do** (1) Sample the domain according to the variant strategy. (2) Compute $\phi_{t,0}^{n+1}$ through the variant solver. (3) Compute ρ(1) from Equation (13) using a spatial transformer for m(1). (4) Compute ρ(t) through the variant solver. (5) Compute v(t) from Equation (13). (6) Compute the image similarity, the regularization losses, and the loss function. (7) Perform backward propagation on ${\left(D\phi_{t,0}\cdot v_{t}\right)}^{\theta }$ and ${\left(\nabla \cdot \left(\rho_{t}\cdot v_{t}\right)\right)}^{\theta }$. (8) Update ${\left({\left(D\phi_{t,0}\cdot v_{t}\right)}^{\theta }\right)}^{n+1}$ and ${\left({\left(\nabla \cdot \left(\rho_{t}\cdot v_{t}\right)\right)}^{\theta }\right)}^{n+1}$. **end** Select the best-performing ${\left({\left(D\phi_{t,0}\cdot v_{t}\right)}^{\theta }\right)}^{n+1}$ and ${\left({\left(\nabla \cdot \left(\rho_{t}\cdot v_{t}\right)\right)}^{\theta }\right)}^{n+1}$ according to the stopping strategy.
