# Supplementary material for: Understanding Trade-Offs in Continuous Neural Representations for Diffeomorphic Image Registration: A Comparative Study of Implicit Neural Representations and Neural Ordinary Differential Equations
Source: J Imaging. 2026 Aug 14;12(8):384. doi: 10.3390/jimaging12080384 (PMC13514420; doi:10.3390/jimaging12080384)
Supplement: Supplementary file 1 [file jimaging-12-00384-s001.zip › jimaging-4442611-supplementary.pdf]

# Supplementary Materials: Understanding Trade-Offs in Continuous Neural Representations for Diffeomorphic Image Registration: A Comparative Study of Implicit Neural Representations and Neural Ordinary Differential Equations

**Salvador Rodriguez-Sanz [1,2] and Carlos Paesa-Lia [1,2] and Monica Hernandez [1,2*]**

## S1. Abbreviations used in this work

ANTS - Advanced Normalization ToolS
CNN - Convolutional Neural Network
CPU - Central Processing Unit
CT - Computed Tomography
def. eq. - deformation equation
DSC - Dice Similarity Coefficient
GPU - Graphics Processing Unit
id - identity
INRs - Implicit Neural Representations
LDDMM - Large Deformation Diffeomorphic Metric Mapping
Learn2Reg - Learn To Register
lNCC - local Normalized Cross-Correlation
MI - Mutual Information
MLP - Multi-Layer Perceptron
MRI - Magnetic Resonance Imaging
NCC - Normalized Cross-Correlation
NGF - Normalized Gradient Fields
NIREP - Non-rigid Image Registration Evaluation Project
NODES - Neural Ordinary Differential EquationS
OASIS - Open Access Series of Imaging Studies
ODE - Ordinary Differential Equation
PDE - Partial Differential Equation
rhs - right-hand-side
RK - Runge-Kutta
RKHS - Reproducing Kernel Hilbert Space
SIRENs - SInusoidal REpresentation Networks
SS - Scaling and Squaring
SSD - Sum of Squared Differences
st. eq. - state equation
VRAM - Video Random Access Memory

## S2. Remarks on the selection of parameters

### *S2.1. Number of epochs*

Regarding the selection of the maximum number of epochs, we opted for a comparison of all the approaches under a common optimization budget, similarly to the experimental protocol adopted by Sun et al. The use of a common optimization budget enables a controlled comparison of architectures and formulations under identical training conditions, avoiding the introduction of method-specific stopping criteria. Sun et al. reported convergence analyses up to 900 epochs and observed that DSC values largely stabilized between 100 and 300 epochs, whereas the number of negative Jacobians continued to increase with further training. For the hybrid method, the authors identified 200 epochs as optimal for the first stage, although no single optimal number of epochs was specified for the remaining methods. In our study, we conducted a convergence analysis up to a maximum of 300 epochs. This choice is also consistent with our previous work, where 300 epochs was

found to provide a good balance between registration accuracy, deformation quality, and computational efficiency.

### *S2.2. Encoding functions*

The value proposed in Sun et al. was 128 while Byra et al. proposed to use 6 encoding functions. We adopted the same value as Byra et al. since some variants in our study employ 3D+t Siren architectures, for which a larger number of encoding functions led to efficiency and convergence issues.

### *S2.3. Downsize step*

The value proposed in Sun et al. was 3 while we used 4. This reduced value was selected to improve computational efficiency while maintaining performance.

### *S2.4. Parameter samples for patch and hybrid methods*

While we used 1200 samples, Sun et al. reported 5 patches per optimization iteration, which means that for 300 epochs the method would use 1500 samples. We slightly reduced the number of samples for efficiency.

## S3. Results

### *S3.1. NIREP results with downsize, hybrid, and downsample-based sampling*

Tables S1 and S2 show the mean and standard deviation of the evaluation metrics obtained after registration after 100 and 150 iterations for the selection of variants in the main manuscript. In particular, we show the results obtained for Siren-LDDMM and Siren-PDE-LDDMM for downsize, hybrid, and downsample-based sampling strategies and for NODEO-LDDMM and NODEO-PDE-LDDMM for the downsample-based strategy.

### *S3.2. Computational Complexity*

#### S3.2.1. Memory requirements

Table S3 shows the GPU memory requirements of our proposed methods. For Siren-LDDMM, the most efficient variants are achieved with the downsize sampling strategy and SS, Euler, or RK solvers. The memory load required by the downsize strategy combined with NODEs is notably higher for LDDMM but similar to traditional solvers for PDE-LDDMM. The second most efficient variants are obtained with the hybrid strategy followed by downsampling strategy combined with traditional ODE solvers. It draws our attention the memory load needed by the downsampling strategy combined with NODE solvers, superior to 10 GB in most cases. Finally, NODEO-LDDMM was much more memory-efficient than Siren-LDDMM. On the other hand, NODEO-PDE-LDDMM showed a memory load similar to that of the corresponding Siren-PDE-LDDMM variants.

#### S3.2.2. Computation time and memory of hybrid-based sampling methods

Table S4 shows the computation time and the maximum memory load achieved by the registration methods with hybrid-based sampling in one representative experiment.

### *S3.3. Wilcoxon rank-sum tests in NIREP and OASIS*

Figure S1 shows the complete results of pairwise right-tailed Wilcoxon rank-sum tests that were conducted for the assessment of the statistical significance of the difference of medians for the distribution of the DSC values. The alternative hypothesis is that the median of the first distribution is higher than the median of the second one.

It should be noted the limitation of the NIREP evaluation on the relatively small number of available registration pairs (15). Consequently, the corresponding statistical tests should be interpreted with appropriate caution, as the statistical power is lower than in

larger contemporary benchmarks such as OASIS Learn2Reg test (39). For this reason, the NIREP results should be considered jointly with the OASIS experiments, which provide a substantially larger evaluation set.

### *S3.4. NIREP baseline qualitative results*

Figures S2 and S3 present sagittal views of the displacement fields and the transformation grids of the baseline methods for a reference with the qualitative results shown in the main manuscript.

### *S3.5. NIREP qualitative results*

Figure S4 shows sagittal views of the differences after registration of the methods with RK and NODE-RK solvers. Figures S5 and S6 present sagittal views of the displacement fields and the transformation grids of the different variants. The results are visually similar to those shown in the main manuscript for Euler solvers.

### *S3.6. OASIS qualitative results*

Figure S7 shows sagittal views of the differences after registration of the methods with RK and NODE-RK solvers. Figures S8 and S9 present sagittal views of the displacement fields and the transformation grids of the different variants. The results are visually similar to those shown in the main manuscript for Euler solvers. The main differences can be appreciated from the grid representation of the transformations for some variants.

**Table S1.** NIREP. Evaluation results obtained from Siren-LDDMM, Siren-PDE-LDDMM, NODEO-LDDMM, and NODEO-PDE-LDDMM. 100 its. Continues in Table S2 and Table 4 in the main manuscript. Comparison of the performance of the methods across different solver configurations (SS, Euler, RK, and stationary NODEs) and downsize, hybrid, and downsample sampling strategies. The evaluation metrics include the mean and standard deviation of the Dice Similarity Coefficient (DSC), maximum and minimum Jacobian determinant, and the count of non-positive Jacobian determinants. The arrows indicate that high DSC values while not extreme Jacobian determinant values are preferable. The mean computation time is included in the table for a complementary assessment. Boldface indicates, for each family of methods, the one with the highest DSC average. The grey cells highlight the solvers with the highest DSC average within each family of methods. Red cells highlight methods with a number of negative Jacobians above one thousand. Light grey indicates the metrics reached with downsize strategy for the hybrid sampling.

NIREP, Siren-LDDMM and Siren-PDE-LDMM, K = id, 100 its

| Method | DSC (%) ↑ | max(J) ↓ | min(J) ↑ | # of $\lvert J_\phi \rvert \leq 0$ ↓ | time$_{GPU}$ (s) ↓ |
|---|---|---|---|---|---|
| St. LDDMM, SS, Downsize | $60.07 \pm 1.46$ | $9.76 \pm 2.61$ | $-0.33 \pm 0.22$ | 594 ($6.47e^{-4}$) | 36.47 |
| St. LDDMM, SS, Downsize ↷ | $60.04 \pm 1.43$ | $9.28 \pm 2.66$ | $-0.52 \pm 0.53$ | 795 ($8.66e^{-4}$) | ↷ |
| St. LDDMM, SS, Hybrid | $59.19 \pm 1.76$ | $9.61 \pm 2.63$ | $-0.56 \pm 0.55$ | 828 ($9.01e^{-4}$) | 202.96 |
| St. LDDMM, SS, Downsample | $57.85 \pm 2.22$ | $7.82 \pm 1.49$ | $0.36 \pm 0.04$ | 0 | 114.66 |
| St. LDDMM, Euler, Downsize | $60.56 \pm 1.25$ | $14.30 \pm 3.85$ | $-0.50 \pm 0.31$ | 1318 ($1.43e^{-3}$) | 36.47 |
| St. LDDMM, Euler, Downsize ↷ | $60.52 \pm 1.24$ | $13.64 \pm 3.66$ | $-0.42 \pm 0.25$ | 920 ($1.00e^{-3}$) | ↷ |
| St. LDDMM, Euler, Hybrid | $60.41 \pm 1.23$ | $14.05 \pm 3.59$ | $-0.47 \pm 0.25$ | 985 ($1.07e^{-3}$) | 205.44 |
| St. LDDMM, Euler, Downsample | $58.12 \pm 1.51$ | $8.57 \pm 3.31$ | $0.40 \pm 0.03$ | 0 | 115.41 |
| St. LDDMM, St. NODE, Euler, Downsize | $61.32 \pm 1.27$ | $25.36 \pm 12.22$ | $-0.40 \pm 1.01$ | 76 ($8.27e^{-5}$) | 114.04 |
| St. LDDMM, St. NODE, Euler, Downsize ↷ | $61.24 \pm 1.37$ | $26.39 \pm 10.87$ | $-0.83 \pm 2.77$ | 71 ($7.73e^{-5}$) | ↷ |
| St. LDDMM, St. NODE, Euler, Hybrid | $60.94 \pm 1.52$ | $26.13 \pm 10.43$ | $-1.10 \pm 2.82$ | 97 ($1.06e^{-4}$) | 387.60 |
| St. LDDMM, St. NODE, Euler, Downsample | $59.38 \pm 1.39$ | $9.80 \pm 2.42$ | $0.37 \pm 0.03$ | 0 | 147.44 |
| St. LDDMM, RK, Downsize | $60.48 \pm 1.25$ | $12.92 \pm 3.51$ | $-0.75 \pm 0.79$ | 1289 ($1.40e^{-3}$) | 31.02 |
| St. LDDMM, RK, Downsize ↷ | $60.49 \pm 1.33$ | $13.55 \pm 3.97$ | $-0.55 \pm 0.59$ | 1088 ($1.18e^{-3}$) | ↷ |
| St. LDDMM, RK, Hybrid | $60.24 \pm 1.39$ | $12.80 \pm 3.67$ | $-0.42 \pm 0.52$ | 598 ($6.51e^{-4}$) | 205.75 |
| St. LDDMM, RK, Downsample | $58.18 \pm 1.34$ | $8.16 \pm 1.84$ | $0.40 \pm 0.02$ | 0 | 120.40 |
| St. LDDMM, St. NODE, RK, Downsize | $\mathbf{61.41 \pm 1.41}$ | $31.29 \pm 21.70$ | $-1.05 \pm 1.56$ | 44 ($4.79e^{-5}$) | 239.85 |
| St. LDDMM, St. NODE, RK, Downsize ↷ | $61.51 \pm 1.32$ | $32.29 \pm 18.92$ | $-0.73 \pm 2.26$ | 39 ($4.25e^{-5}$) | ↷ |
| St. LDDMM, St. NODE, RK, Hybrid | $61.36 \pm 1.26$ | $32.51 \pm 18.73$ | $-0.72 \pm 1.67$ | 52 ($5.66e^{-5}$) | 731.06 |
| St. LDDMM, St. NODE, RK, Downsample | $59.18 \pm 1.56$ | $10.45 \pm 2.51$ | $0.37 \pm 0.03$ | 0 | 197.68 |
| PDE-LDDMM$_{st\ eq}$, Euler, Downsize | $60.34 \pm 1.26$ | $14.70 \pm 4.47$ | $-0.50 \pm 0.40$ | 1053 ($1.15e^{-3}$) | 37.73 |
| PDE-LDDMM$_{st\ eq}$, Euler, Downsize ↷ | $60.38 \pm 1.20$ | $16.38 \pm 5.78$ | $-0.70 \pm 0.57$ | 1435 ($1.56e^{-3}$) | ↷ |
| PDE-LDDMM$_{st\ eq}$, Euler, Hybrid | $60.18 \pm 1.23$ | $16.78 \pm 5.47$ | $-0.80 \pm 0.59$ | 1510 ($1.64e^{-3}$) | 72.55 |
| PDE-LDDMM$_{st\ eq}$, Euler, Downsample | $58.11 \pm 1.21$ | $8.55 \pm 2.33$ | $0.39 \pm 0.04$ | 0 | 129.26 |
| PDE-LDDMM$_{st\ eq}$, St. NODE, Euler, Downsize | $61.19 \pm 1.38$ | $24.16 \pm 8.55$ | $-0.47 \pm 1.57$ | 43 ($4.68e^{-5}$) | 132.47 |
| PDE-LDDMM$_{st\ eq}$, St. NODE, Euler, Downsize ↷ | $61.28 \pm 1.29$ | $23.77 \pm 10.41$ | $-0.54 \pm 2.05$ | 75 ($8.17e^{-5}$) | ↷ |
| PDE-LDDMM$_{st\ eq}$, St. NODE, Euler, Hybrid | $61.12 \pm 1.39$ | $23.89 \pm 9.84$ | $-0.80 \pm 1.95$ | 120 ($1.31e^{-4}$) | 389.68 |
| PDE-LDDMM$_{st\ eq}$, St. NODE, Euler, Downsample | $58.81 \pm 1.53$ | $9.50 \pm 1.48$ | $0.38 \pm 0.03$ | 0 | 148.33 |
| PDE-LDDMM$_{st\ eq}$, RK, Downsize | $60.36 \pm 1.46$ | $12.35 \pm 2.59$ | $-0.56 \pm 0.50$ | 1053 ($1.15e^{-3}$) | 37.86 |
| PDE-LDDMM$_{st\ eq}$, RK, Downsize ↷ | $60.41 \pm 1.33$ | $14.46 \pm 4.61$ | $-0.72 \pm 0.56$ | 1190 ($1.30e^{-3}$) | ↷ |
| PDE-LDDMM$_{st\ eq}$, RK, Hybrid | $60.23 \pm 1.31$ | $15.13 \pm 4.53$ | $-0.81 \pm 0.55$ | 1250 ($1.36e^{-3}$) | 74.05 |
| PDE-LDDMM$_{st\ eq}$, RK, Downsample | $58.15 \pm 1.21$ | $8.54 \pm 3.15$ | $0.39 \pm 0.05$ | 0 | 139.33 |
| PDE-LDDMM$_{st\ eq}$, St. NODE, RK, Downsize | $\mathbf{61.30 \pm 1.38}$ | $30.84 \pm 7.47$ | $-0.74 \pm 1.92$ | 37 ($4.03e^{-5}$) | 386.05 |
| PDE-LDDMM$_{st\ eq}$, St. NODE, RK, Downsize ↷ | $61.30 \pm 1.41$ | $27.37 \pm 12.95$ | $-0.26 \pm 0.56$ | 56 ($6.10e^{-5}$) | ↷ |
| PDE-LDDMM$_{st\ eq}$, St. NODE, RK, Hybrid | $60.96 \pm 1.46$ | $27.55 \pm 12.27$ | $-0.48 \pm 0.48$ | 87 ($9.47e^{-5}$) | 1058.52 |
| PDE-LDDMM$_{st\ eq}$, St. NODE, RK, Downsample | $58.86 \pm 1.51$ | $9.95 \pm 1.52$ | $0.39 \pm 0.04$ | 0 | 217.82 |
| PDE-LDDMM$_{def\ eq}$, Euler, Downsize | $60.31 \pm 1.29$ | $15.05 \pm 3.05$ | $-0.72 \pm 0.52$ | 1417 ($1.54e^{-3}$) | 37.10 |
| PDE-LDDMM$_{def\ eq}$, Euler, Downsize ↷ | $60.33 \pm 1.38$ | $13.90 \pm 3.58$ | $-0.51 \pm 0.40$ | 962 ($1.05e^{-3}$) | ↷ |
| PDE-LDDMM$_{def\ eq}$, Euler, Hybrid | $60.21 \pm 1.29$ | $14.26 \pm 3.70$ | $-0.56 \pm 0.41$ | 1000 ($1.09e^{-3}$) | 69.82 |
| PDE-LDDMM$_{def\ eq}$, Euler, Downsample | $57.98 \pm 1.32$ | $8.60 \pm 2.85$ | $0.40 \pm 0.04$ | 0 | 124.84 |
| PDE-LDDMM$_{def\ eq}$, St. NODE, Euler, Downsize | $61.29 \pm 1.32$ | $27.21 \pm 11.53$ | $-0.61 \pm 2.04$ | 134 ($1.46e^{-4}$) | 131.63 |
| PDE-LDDMM$_{def\ eq}$, St. NODE, Euler, Downsize ↷ | $61.21 \pm 1.25$ | $28.85 \pm 22.32$ | $-0.51 \pm 0.81$ | 66 ($7.19e^{-5}$) | ↷ |
| PDE-LDDMM$_{def\ eq}$, St. NODE, Euler, Hybrid | $61.10 \pm 1.38$ | $29.89 \pm 24.74$ | $-0.93 \pm 0.86$ | 169 ($1.84e^{-4}$) | 327.59 |
| PDE-LDDMM$_{def\ eq}$, St. NODE, Euler, Downsample | $58.96 \pm 1.34$ | $10.57 \pm 2.50$ | $0.38 \pm 0.03$ | 0 | 138.36 |
| PDE-LDDMM$_{def\ eq}$, RK, Downsize | $60.42 \pm 1.35$ | $12.81 \pm 3.09$ | $-0.59 \pm 0.58$ | 1102 ($1.20e^{-3}$) | 37.57 |
| PDE-LDDMM$_{def\ eq}$, RK, Downsize ↷ | $60.38 \pm 1.34$ | $13.64 \pm 3.65$ | $-0.56 \pm 0.36$ | 1258 ($1.37e^{-3}$) | ↷ |
| PDE-LDDMM$_{def\ eq}$, RK, Hybrid | $60.11 \pm 1.42$ | $12.78 \pm 3.62$ | $-0.43 \pm 0.32$ | 678 ($7.38e^{-4}$) | 71.45 |
| PDE-LDDMM$_{def\ eq}$, RK, Downsample | $58.02 \pm 1.40$ | $8.96 \pm 2.82$ | $0.39 \pm 0.05$ | 0 | 135.03 |
| PDE-LDDMM$_{def\ eq}$, St. NODE, RK, Downsize | $\mathbf{61.42 \pm 1.36}$ | $30.79 \pm 26.21$ | $-0.97 \pm 2.49$ | 41 ($4.46e^{-5}$) | 384.59 |
| PDE-LDDMM$_{def\ eq}$, St. NODE, RK, Downsize ↷ | $61.42 \pm 1.28$ | $29.36 \pm 12.94$ | $-0.71 \pm 0.84$ | 92 ($1.00e^{-4}$) | ↷ |
| PDE-LDDMM$_{def\ eq}$, St. NODE, RK, Hybrid | $61.27 \pm 1.32$ | $29.99 \pm 13.51$ | $-1.04 \pm 0.90$ | 174 ($1.89e^{-4}$) | 897.86 |
| PDE-LDDMM$_{def\ eq}$, St. NODE, RK, Downsample | $58.89 \pm 1.38$ | $11.40 \pm 2.55$ | $0.39 \pm 0.04$ | 0 | 207.85 |

NIREP, NODEO-LDDMM and NODEO-PDE-LDMM, $K = (L^\dagger L)^{-1}$, 100 its

| Method | DSC (%) ↑ | max(J) ↓ | min(J) ↑ | # of $\lvert J_\phi \rvert \leq 0$ ↓ | time$_{GPU}$ (s) ↓ |
|---|---|---|---|---|---|
| St. LDDMM, St. NODE, Euler, Downsample | $\mathbf{58.11 \pm 2.31}$ | $4.22 \pm 1.68$ | $0.47 \pm 0.11$ | 0 | 20.25 |
| St. LDDMM, St. NODE, RK, Downsample | $57.56 \pm 2.46$ | $3.95 \pm 1.13$ | $0.46 \pm 0.11$ | 0 | 26.77 |
| PDE-LDDMM$_{st\ eq}$, St. NODE, Euler, Downsample | $57.65 \pm 2.15$ | $4.04 \pm 0.97$ | $0.43 \pm 0.03$ | 0 | 62.58 |
| PDE-LDDMM$_{st\ eq}$, St. NODE, RK, Downsample | $56.66 \pm 3.36$ | $3.65 \pm 1.13$ | $0.45 \pm 0.07$ | 0 | 93.61 |
| PDE-LDDMM$_{def\ eq}$, St. NODE, Euler, Downsample | $56.47 \pm 3.09$ | $4.17 \pm 1.83$ | $0.44 \pm 0.04$ | 0 | 40.49 |
| PDE-LDDMM$_{def\ eq}$, St. NODE, RK, Downsample | $56.15 \pm 4.06$ | $3.85 \pm 1.32$ | $0.47 \pm 0.11$ | 0 | 59.94 |

**Table S2.** NIREP. Evaluation results obtained from Siren-LDDMM, Siren-PDE-LDDMM, NODEO-LDDMM, and NODEO-PDE-LDDMM. 150 its. Continues in Table 4 in the main manuscript. Comparison of the performance of the methods across different solver configurations (SS, Euler, RK, and stationary NODEs) and downsize, hybrid, and downsample sampling strategies. The evaluation metrics include the mean and standard deviation of the Dice Similarity Coefficient (DSC), maximum and minimum Jacobian determinant, and the count of non-positive Jacobian determinants. The arrows indicate that high DSC values while not extreme Jacobian determinant values are preferable. The mean computation time is included in the table for a complementary assessment. Boldface indicates, for each family of methods, the one with the highest DSC average. The grey cells highlight the solvers with the highest DSC average within each family of methods. Red cells highlight methods with a number of negative Jacobians above one thousand. Light grey indicates the metrics reached with downsize strategy for the hybrid sampling.

NIREP, Siren-LDDMM and Siren-PDE-LDMM, K = id, 150 its

| Method | DSC (%) ↑ | max(J) ↓ | min(J) ↑ | # of $\lvert J_\phi \rvert \leq 0$ ↓ | $\text{time}_{\text{GPU}}$ (s) ↓ |
|---|---|---|---|---|---|
| St. LDDMM, SS, Downsize | $60.41 \pm 1.43$ | $9.74 \pm 2.62$ | $-0.38 \pm 0.21$ | 888 ($9.67e^{-4}$) | 48.12 |
| St. LDDMM, SS, Downsize ↷ | $60.40 \pm 1.45$ | $9.84 \pm 2.28$ | $-0.50 \pm 0.49$ | 948 ($1.03e^{-3}$) | ↷ |
| St. LDDMM, SS, Hybrid | $59.39 \pm 1.78$ | $10.24 \pm 2.18$ | $-0.61 \pm 0.58$ | 981 ($1.07e^{-3}$) | 214.01 |
| St. LDDMM, SS, Downsample | $59.72 \pm 2.21$ | $10.44 \pm 1.94$ | $0.20 \pm 0.11$ | 0 | 171.98 |
| St. LDDMM, Euler, Downsize | $61.06 \pm 1.24$ | $13.93 \pm 3.22$ | $-1.00 \pm 0.57$ | 2435 ($2.65e^{-3}$) | 39.49 |
| St. LDDMM, Euler, Donwsize ↷ | $60.99 \pm 1.31$ | $14.97 \pm 3.46$ | $-0.85 \pm 0.37$ | 2160 ($2.35e^{-3}$) | ↷ |
| St. LDDMM, Euler, Hybrid | $60.87 \pm 1.30$ | $15.50 \pm 3.49$ | $-0.94 \pm 0.31$ | 2282 ($2.48e^{-3}$) | 213.78 |
| St. LDDMM, Euler, Downsample | $59.80 \pm 1.45$ | $10.06 \pm 1.88$ | $0.29 \pm 0.08$ | 0 | 173.16 |
| St. LDDMM, St. NODE, Euler, Downsize | $61.68 \pm 1.37$ | $25.20 \pm 11.73$ | $-0.78 \pm 1.78$ | 223 ($2.43e^{-4}$) | 204.45 |
| St. LDDMM, St. NODE, Euler, Downsize ↷ | $61.65 \pm 1.40$ | $24.01 \pm 8.11$ | $-0.80 \pm 2.83$ | 132 ($1.44e^{-4}$) | ↷ |
| St. LDDMM, St. NODE, Euler, Hybrid | $61.59 \pm 1.38$ | $24.15 \pm 7.83$ | $-1.05 \pm 3.08$ | 188 ($2.05e^{-4}$) | 440.69 |
| St. LDDMM, St. NODE, Euler, Downsample | $61.10 \pm 1.47$ | $12.40 \pm 3.76$ | $0.22 \pm 0.08$ | 0 | 234.95 |
| St. LDDMM, RK, Downsize | $60.98 \pm 1.34$ | $14.04 \pm 3.74$ | $-1.10 \pm 0.80$ | 2082 ($2.27e^{-3}$) | 39.57 |
| St. LDDMM, RK, Downsize ↷ | $60.96 \pm 1.31$ | $13.26 \pm 3.50$ | $-0.94 \pm 0.70$ | 1962 ($2.14e^{-3}$) | ↷ |
| St. LDDMM, RK, Hybrid | $60.65 \pm 1.33$ | $12.84 \pm 3.71$ | $-0.79 \pm 0.68$ | 1088 ($1.18e^{-3}$) | 213.05 |
| St. LDDMM, RK, Downsample | $59.90 \pm 1.49$ | $10.34 \pm 2.68$ | $0.27 \pm 0.08$ | 0 | 180.44 |
| St. LDDMM, St. NODE, RK, Downsize | **$61.85 \pm 1.38$** | $34.24 \pm 19.29$ | $-1.24 \pm 2.08$ | 88 ($9.58e^{-5}$) | 370.43 |
| St. LDDMM, St. NODE, RK, Downsize ↷ | $61.87 \pm 1.35$ | $28.75 \pm 11.07$ | $-0.63 \pm 0.70$ | 96 ($1.05e^{-4}$) | ↷ |
| St. LDDMM, St. NODE, RK, Hybrid | $61.77 \pm 1.27$ | $28.98 \pm 10.98$ | $-0.83 \pm 0.74$ | 136 ($1.48e^{-4}$) | 846.40 |
| St. LDDMM, St. NODE, RK, Downsample | $61.04 \pm 1.43$ | $13.23 \pm 4.76$ | $0.24 \pm 0.07$ | 0 | 334.33 |
| $\text{PDE-LDDMM}_{\text{st eq}}$, Euler, Downsize | $60.87 \pm 1.35$ | $16.42 \pm 5.35$ | $-1.02 \pm 0.43$ | 2912 ($3.17e^{-3}$) | 49.63 |
| $\text{PDE-LDDMM}_{\text{st eq}}$, Euler, Downsize ↷ | $60.82 \pm 1.27$ | $17.19 \pm 5.90$ | $-0.90 \pm 0.58$ | 2514 ($2.74e^{-3}$) | ↷ |
| $\text{PDE-LDDMM}_{\text{st eq}}$, Euler, Hybrid | $60.67 \pm 1.24$ | $17.59 \pm 5.38$ | $-1.01 \pm 0.66$ | 2588 ($2.82e^{-3}$) | 84.61 |
| $\text{PDE-LDDMM}_{\text{st eq}}$, Euler, Downsample | $59.93 \pm 1.36$ | $11.00 \pm 2.76$ | $0.31 \pm 0.07$ | 0 | 193.83 |
| $\text{PDE-LDDMM}_{\text{st eq}}$, St. NODE, Euler, Downsize | $61.54 \pm 1.41$ | $24.08 \pm 7.83$ | $-0.65 \pm 1.28$ | 96 ($1.05e^{-4}$) | 190.55 |
| $\text{PDE-LDDMM}_{\text{st eq}}$, St. NODE, Euler, Downsize ↷ | $61.64 \pm 1.30$ | $25.12 \pm 7.13$ | $-0.90 \pm 2.85$ | 139 ($1.51e^{-4}$) | ↷ |
| $\text{PDE-LDDMM}_{\text{st eq}}$, St. NODE, Euler, Hybrid | $61.52 \pm 1.46$ | $25.34 \pm 7.49$ | $-1.10 \pm 2.95$ | 214 ($2.33e^{-4}$) | 463.85 |
| $\text{PDE-LDDMM}_{\text{st eq}}$, St. NODE, Euler, Downsample | $60.65 \pm 1.41$ | $11.31 \pm 1.84$ | $0.29 \pm 0.05$ | 0 | 222.48 |
| $\text{PDE-LDDMM}_{\text{st eq}}$, RK, Downsize | $60.93 \pm 1.40$ | $13.48 \pm 3.05$ | $-0.81 \pm 0.49$ | 2324 ($2.53e^{-3}$) | 49.90 |
| $\text{PDE-LDDMM}_{\text{st eq}}$, RK, Downsize ↷ | $60.84 \pm 1.34$ | $15.13 \pm 3.90$ | $-1.13 \pm 0.53$ | 2138 ($2.33e^{-3}$) | ↷ |
| $\text{PDE-LDDMM}_{\text{st eq}}$, RK, Hybrid | $60.69 \pm 1.34$ | $15.62 \pm 3.76$ | $-1.19 \pm 0.58$ | 2264 ($2.46e^{-3}$) | 86.23 |
| $\text{PDE-LDDMM}_{\text{st eq}}$, RK, Downsample | $59.95 \pm 1.39$ | $10.57 \pm 2.99$ | $0.27 \pm 0.08$ | 0 | 209.11 |
| $\text{PDE-LDDMM}_{\text{st eq}}$, St. NODE, RK, Downsize | **$61.69 \pm 1.36$** | $40.40 \pm 24.33$ | $-0.73 \pm 0.90$ | 113 ($1.23e^{-4}$) | 568.04 |
| $\text{PDE-LDDMM}_{\text{st eq}}$, St. NODE, RK, Downsize ↷ | $61.69 \pm 1.33$ | $39.19 \pm 23.10$ | $-0.86 \pm 1.27$ | 159 ($1.73e^{-4}$) | ↷ |
| $\text{PDE-LDDMM}_{\text{st eq}}$, St. NODE, RK, Hybrid | $61.52 \pm 1.34$ | $38.90 \pm 22.56$ | $-1.02 \pm 1.33$ | 181 ($1.97e^{-4}$) | 1268.12 |
| $\text{PDE-LDDMM}_{\text{st eq}}$, St. NODE, RK, Downsample | $60.51 \pm 1.42$ | $12.14 \pm 1.22$ | $0.27 \pm 0.08$ | 0 | 329.30 |
| $\text{PDE-LDDMM}_{\text{def eq}}$, Euler, Downsize | $60.81 \pm 1.31$ | $15.64 \pm 2.73$ | $-1.15 \pm 0.88$ | 2502 ($2.72e^{-3}$) | 48.94 |
| $\text{PDE-LDDMM}_{\text{def eq}}$, Euler, Downsize ↷ | $60.81 \pm 1.27$ | $16.40 \pm 4.58$ | $-0.90 \pm 0.69$ | 2146 ($2.34e^{-3}$) | ↷ |
| $\text{PDE-LDDMM}_{\text{def eq}}$, Euler, Hybrid | $60.71 \pm 1.24$ | $16.57 \pm 4.62$ | $-1.00 \pm 0.73$ | 2196 ($2.39e^{-3}$) | 81.19 |
| $\text{PDE-LDDMM}_{\text{def eq}}$, Euler, Downsample | $59.77 \pm 1.37$ | $10.32 \pm 2.55$ | $0.32 \pm 0.04$ | 0 | 187.43 |
| $\text{PDE-LDDMM}_{\text{def eq}}$, St. NODE, Euler, Downsize | $61.64 \pm 1.31$ | $28.18 \pm 12.23$ | $-0.34 \pm 0.60$ | 100 ($1.09e^{-4}$) | 189.55 |
| $\text{PDE-LDDMM}_{\text{def eq}}$, St. NODE, Euler, Downsize ↷ | $61.62 \pm 1.26$ | $31.39 \pm 19.01$ | $-0.97 \pm 1.24$ | 123 ($1.34e^{-4}$) | ↷ |
| $\text{PDE-LDDMM}_{\text{def eq}}$, St. NODE, Euler, Hybrid | $61.66 \pm 1.27$ | $31.23 \pm 18.13$ | $-1.11 \pm 1.32$ | 354 ($3.85e^{-3}$) | 400.36 |
| $\text{PDE-LDDMM}_{\text{def eq}}$, St. NODE, Euler, Downsample | $60.66 \pm 1.41$ | $12.62 \pm 2.73$ | $0.25 \pm 0.07$ | 0 | 207.53 |
| $\text{PDE-LDDMM}_{\text{def eq}}$, RK, Downsize | $60.92 \pm 1.39$ | $13.81 \pm 3.25$ | $-0.91 \pm 0.56$ | 2191 ($2.39e^{-3}$) | 49.22 |
| $\text{PDE-LDDMM}_{\text{def eq}}$, RK, Downsize ↷ | $60.88 \pm 1.37$ | $14.05 \pm 2.65$ | $-0.96 \pm 0.78$ | 2057 ($2.24e^{-3}$) | ↷ |
| $\text{PDE-LDDMM}_{\text{def eq}}$, RK, Hybrid | $60.66 \pm 1.43$ | $13.47 \pm 2.28$ | $-0.76 \pm 0.62$ | 1111 ($1.21e^{-3}$) | 83.01 |
| $\text{PDE-LDDMM}_{\text{def eq}}$, RK, Downsample | $59.70 \pm 1.49$ | $11.08 \pm 2.88$ | $0.29 \pm 0.07$ | 0 | 202.43 |
| $\text{PDE-LDDMM}_{\text{def eq}}$, St. NODE, RK, Downsize | **$61.79 \pm 1.23$** | $29.86 \pm 19.49$ | $-1.45 \pm 1.69$ | 147 ($1.60e^{-4}$) | 567.18 |
| $\text{PDE-LDDMM}_{\text{def eq}}$, St. NODE, RK, Downsize ↷ | $61.76 \pm 1.35$ | $29.57 \pm 14.75$ | $-1.12 \pm 1.35$ | 101 ($1.01e^{-4}$) | ↷ |
| $\text{PDE-LDDMM}_{\text{def eq}}$, St. NODE, RK, Hybrid | $61.61 \pm 1.36$ | $30.19 \pm 14.09$ | $-2.10 \pm 1.89$ | 230 ($2.50e^{-4}$) | 1098.14 |
| $\text{PDE-LDDMM}_{\text{def eq}}$, St. NODE, RK, Downsample | $60.51 \pm 1.46$ | $13.92 \pm 3.82$ | $0.28 \pm 0.06$ | 0 | 312.68 |

NIREP, NODEO-LDDMM and NODEO-PDE-LDMM, $K = (L^\dagger L)^{-1}$, 150 its

| Method | DSC (%) ↑ | max(J) ↓ | min(J) ↑ | # of $\lvert J_\phi \rvert \leq 0$ ↓ | $\text{time}_{GPU}$ (s) ↓ |
|---|---|---|---|---|---|
| St. LDDMM, St. NODE, Euler, Downsample | **$60.33 \pm 2.11$** | $5.29 \pm 1.78$ | $0.41 \pm 0.14$ | 0 | 29.80 |
| St. LDDMM, St. NODE, RK, Downsample | $60.24 \pm 1.87$ | $5.37 \pm 1.84$ | $0.40 \pm 0.14$ | 0 | 39.85 |
| $\text{PDE-LDDMM}_{\text{st eq}}$, St. NODE, Euler, Downsample | $59.07 \pm 2.74$ | $5.18 \pm 1.68$ | $0.42 \pm 0.06$ | 0 | 93.41 |
| $\text{PDE-LDDMM}_{\text{st eq}}$, St. NODE, RK, Downsample | **$59.76 \pm 1.93$** | $4.77 \pm 0.83$ | $0.42 \pm 0.06$ | 0 | 139.82 |
| $\text{PDE-LDDMM}_{\text{def eq}}$, St. NODE, Euler, Downsample | $58.26 \pm 4.05$ | $4.82 \pm 1.37$ | $0.44 \pm 0.11$ | 0 | 60.08 |
| $\text{PDE-LDDMM}_{\text{def eq}}$, St. NODE, RK, Downsample | $59.05 \pm 2.71$ | $4.99 \pm 1.31$ | $0.41 \pm 0.06$ | 0 | 89.41 |

**Table S3.** NIREP. Maximum VRAM memory usage achieved by the registration methods from Table 4 in the main manuscript.

| NIREP, Siren-LDDMM and Siren-PDE-LDDMM, K = id, 300 its | |
|---|---|
| Method | peak VRAM (MBs) ↓ |
| St. LDDMM, SS, Downsize | 2896 |
| St. LDDMM, SS, Hybrid | 4320 |
| St. LDDMM, SS, Downsample | 6242 |
| St. LDDMM, Euler, Downsize | 2162 |
| St. LDDMM, Euler, Hybrid | 4688 |
| St. LDDMM, Euler, Downsample | 6680 |
| St. LDDMM, St. NODE, Euler, Downsize | 8754 |
| St. LDDMM, St. NODE, Euler, Hybrid | 5670 |
| St. LDDMM, St. NODE, Euler, Downsample | 16108 |
| St. LDDMM, RK, Downsize | 2162 |
| St. LDDMM, RK, Hybrid | 4636 |
| St. LDDMM, RK, Downsample | 7128 |
| St. LDDMM, St. NODE, RK, Downsize | 8860 |
| St. LDDMM, St. NODE, RK, Hybrid | 5670 |
| St. LDDMM, St. NODE, RK, Downsample | 17992 |
| PDE-LDDMM$_{\text{st eq}}$, Euler, Downsize | 4146 |
| PDE-LDDMM$_{\text{st eq}}$, Euler, Hybrid | 4146 |
| PDE-LDDMM$_{\text{st eq}}$, Euler, Downsample | 12834 |
| PDE-LDDMM$_{\text{st eq}}$, St. NODE, Euler, Downsize | 3194 |
| PDE-LDDMM$_{\text{st eq}}$, St. NODE, Euler, Hybrid | 3194 |
| PDE-LDDMM$_{\text{st eq}}$, St. NODE, Euler, Downsample | 9134 |
| PDE-LDDMM$_{\text{st eq}}$, RK, Downsize | 4146 |
| PDE-LDDMM$_{\text{st eq}}$, RK, Hybrid | 4146 |
| PDE-LDDMM$_{\text{st eq}}$, RK, Downsample | 14110 |
| PDE-LDDMM$_{\text{st eq}}$, St. NODE, RK, Downsize | 3512 |
| PDE-LDDMM$_{\text{st eq}}$, St. NODE, RK, Hybrid | 4146 |
| PDE-LDDMM$_{\text{st eq}}$, St. NODE, RK, Downsample | 9550 |
| PDE-LDDMM$_{\text{def eq}}$, Euler, Downsize | 4140 |
| PDE-LDDMM$_{\text{def eq}}$, Euler, Hybrid | 4140 |
| PDE-LDDMM$_{\text{def eq}}$, Euler, Downsample | 12470 |
| PDE-LDDMM$_{\text{def eq}}$, St. NODE, Euler, Downsize | 3190 |
| PDE-LDDMM$_{\text{def eq}}$, St. NODE, Euler, Hybrid | 3192 |
| PDE-LDDMM$_{\text{def eq}}$, St. NODE, Euler, Downsample | 8394 |
| PDE-LDDMM$_{\text{def eq}}$, RK, Downsize | 4138 |
| PDE-LDDMM$_{\text{def eq}}$, RK, Hybrid | 4140 |
| PDE-LDDMM$_{\text{def eq}}$, RK, Downsample | 13348 |
| PDE-LDDMM$_{\text{def eq}}$, St. NODE, RK, Downsize | 3508 |
| PDE-LDDMM$_{\text{def eq}}$, St. NODE, RK, Hybrid | 3510 |
| PDE-LDDMM$_{\text{def eq}}$, St. NODE, RK, Downsample | 9058 |
| NIREP, NODEO-LDDMM and NODEO-PDE-LDMM, $K = (L^{\dagger}L)^{-1}$, 300 its | |
| Method | peak VRAM (MBs) ↓ |
| St. LDDMM, St. NODE, Euler, Downsample | 4272 |
| St. LDDMM, St. NODE, RK, Downsample | 5022 |
| PDE-LDDMM$_{\text{st eq}}$, St. NODE, Euler, Downsample | 9774 |
| PDE-LDDMM$_{\text{st eq}}$, St. NODE, RK, Downsample | 9824 |
| PDE-LDDMM$_{\text{def eq}}$, St. NODE, Euler, Downsample | 6786 |
| PDE-LDDMM$_{\text{def eq}}$, St. NODE, RK, Downsample | 8238 |

**Table S4.** NIREP. Computation time and maximum VRAM memory usage achieved by the registration methods with hybrid-based sampling and NODE-based solvers. Patch size of $64 \times 64 \times 64$. Values are given for 300 iterations for all methods in a single representative experiment.

| Method | time$_{\text{GPU}}$ (s) ↓ | peak VRAM (MBs) ↓ |
|---|---|---|
| St. LDDMM, St. NODE, Euler, Hybrid | 1377.29 | 22072 |
| St. LDDMM, St. NODE, RK, Hybrid | 3512.31 | 22092 |
| PDE-LDDMM$_{\text{st eq}}$, St. NODE, Euler, Hybrid | 2526.33 | 9070 |
| PDE-LDDMM$_{\text{st eq}}$, St. NODE, RK, Hybrid | 8597.55 | 9494 |
| PDE-LDDMM$_{\text{def eq}}$, St. NODE, Euler, Hybrid | 2012.22 | 9066 |
| PDE-LDDMM$_{\text{def eq}}$, St. NODE, RK, Hybrid | 6751.76 | 9490 |

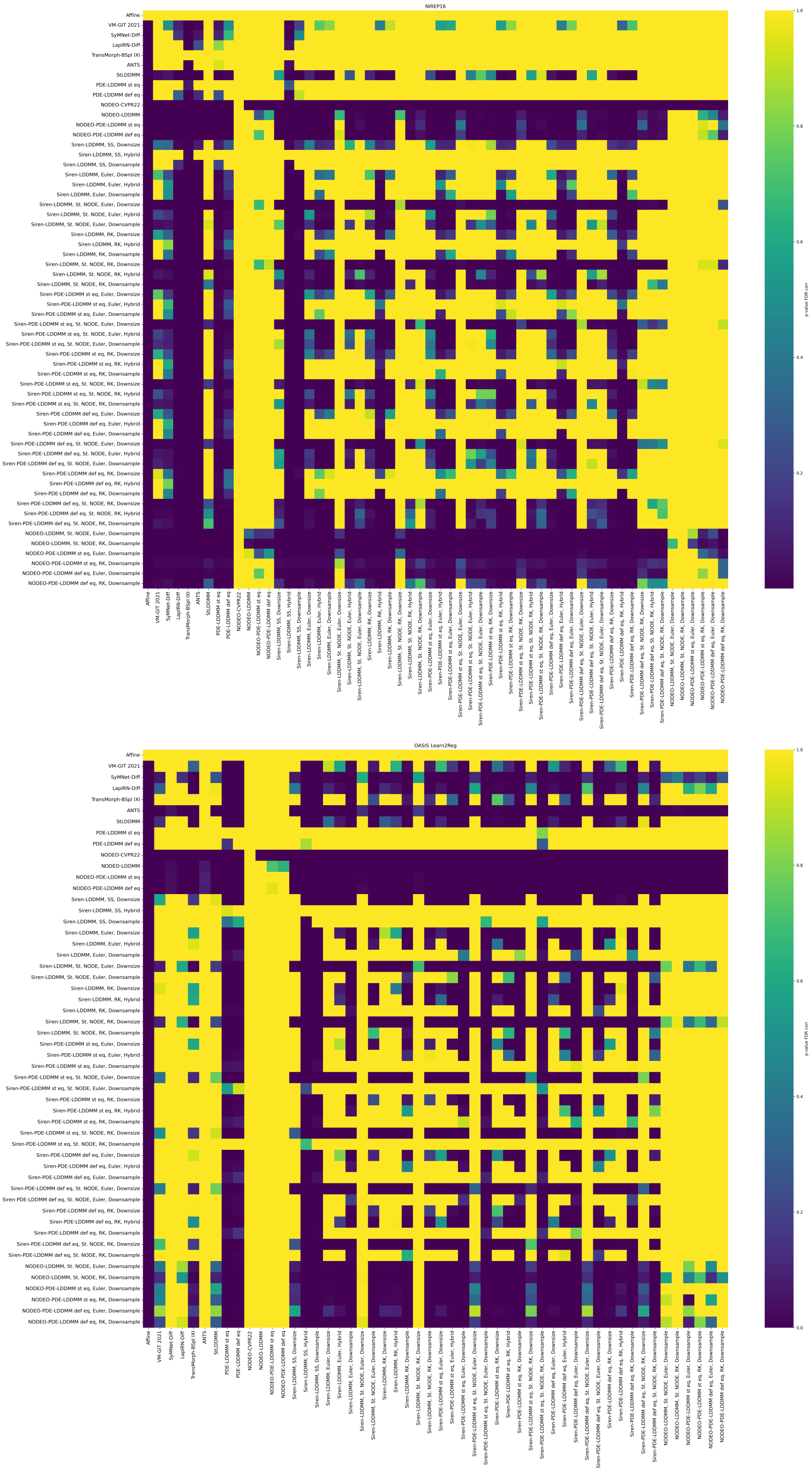


**Figure S1.** Results of the pairwise right-tailed Wilcoxon rank-sum tests to assess the statistical significance of the differences among the methods. The matrix depicts the pairwise p-values between every two methods with False Discovery Rate (FDR) correction. Up, NIREP experiments. Down, OASIS experiments.

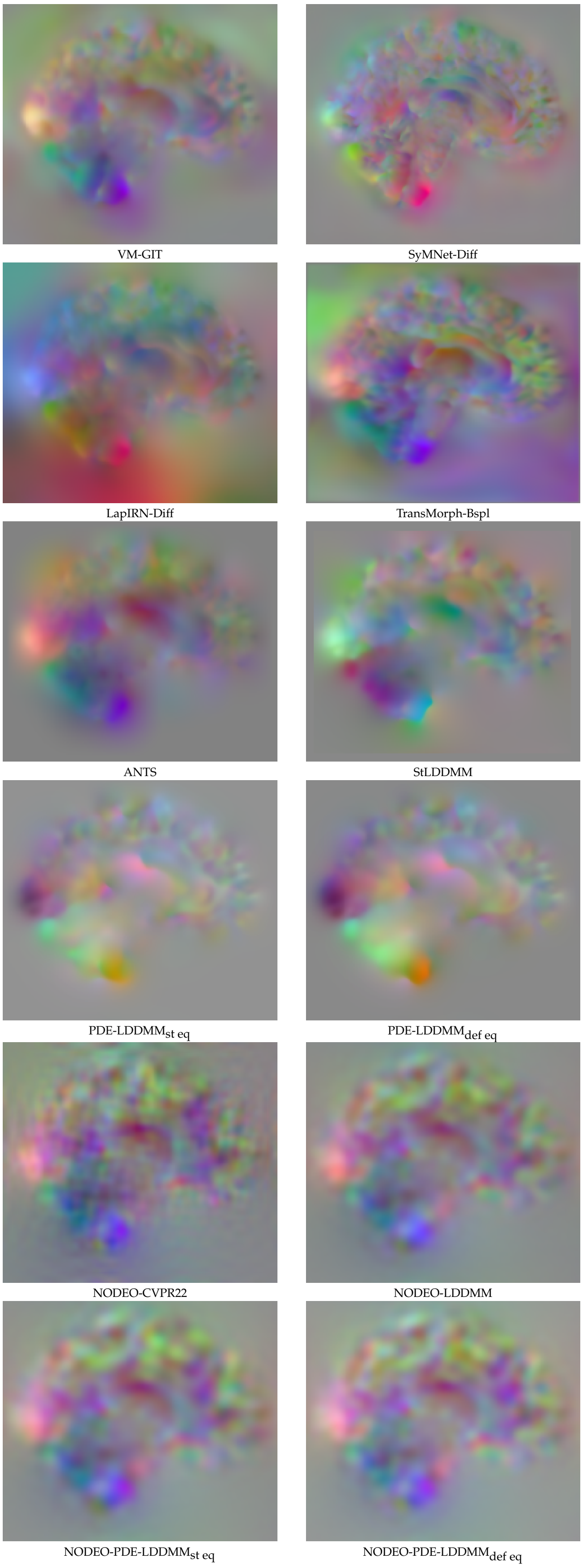


**Figure S2.** NIREP. Baseline methods. Sagittal view of the displacement fields in a representative experiment. The RGB color map proposed in VoxelMorph paper is used for the color representation of the vector fields.

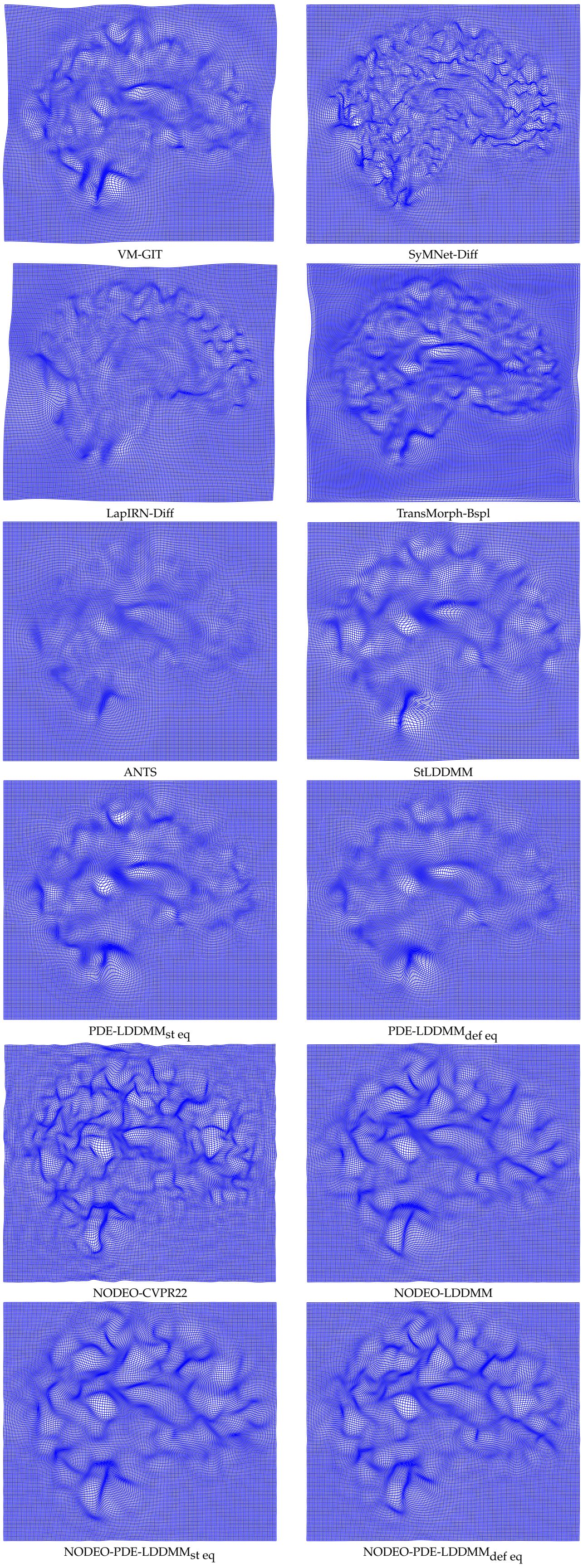


**Figure S3.** NIREP. Baseline methods. Sagittal view of the transformation grids in a representative experiment.

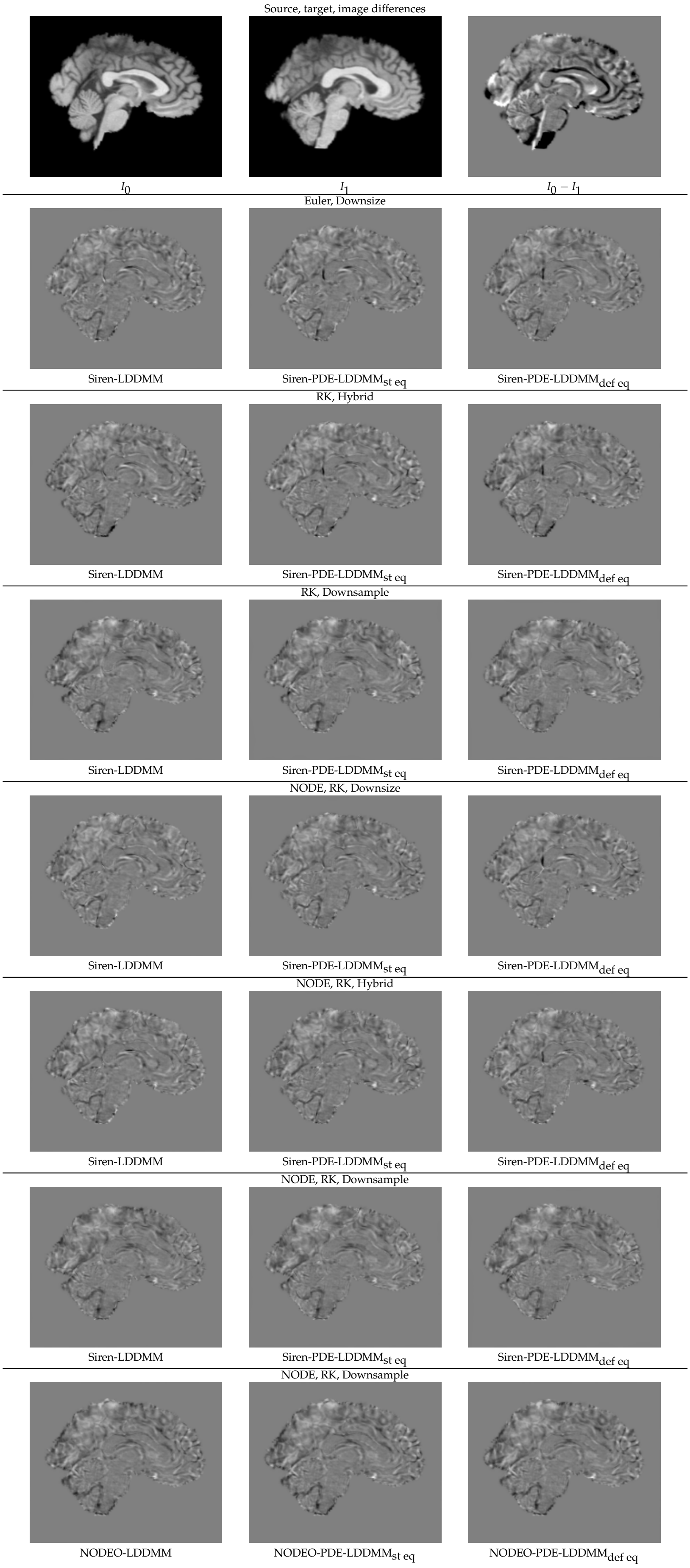


**Figure S4.** NIREP. Sagittal view of the differences after registration $I_0 \circ \varphi^{-1} - I_1$ in a representative experiment. Siren and NODEO methods with RK and NODE-RK solvers.

https://doi.org/10.3390/jimaging12080384

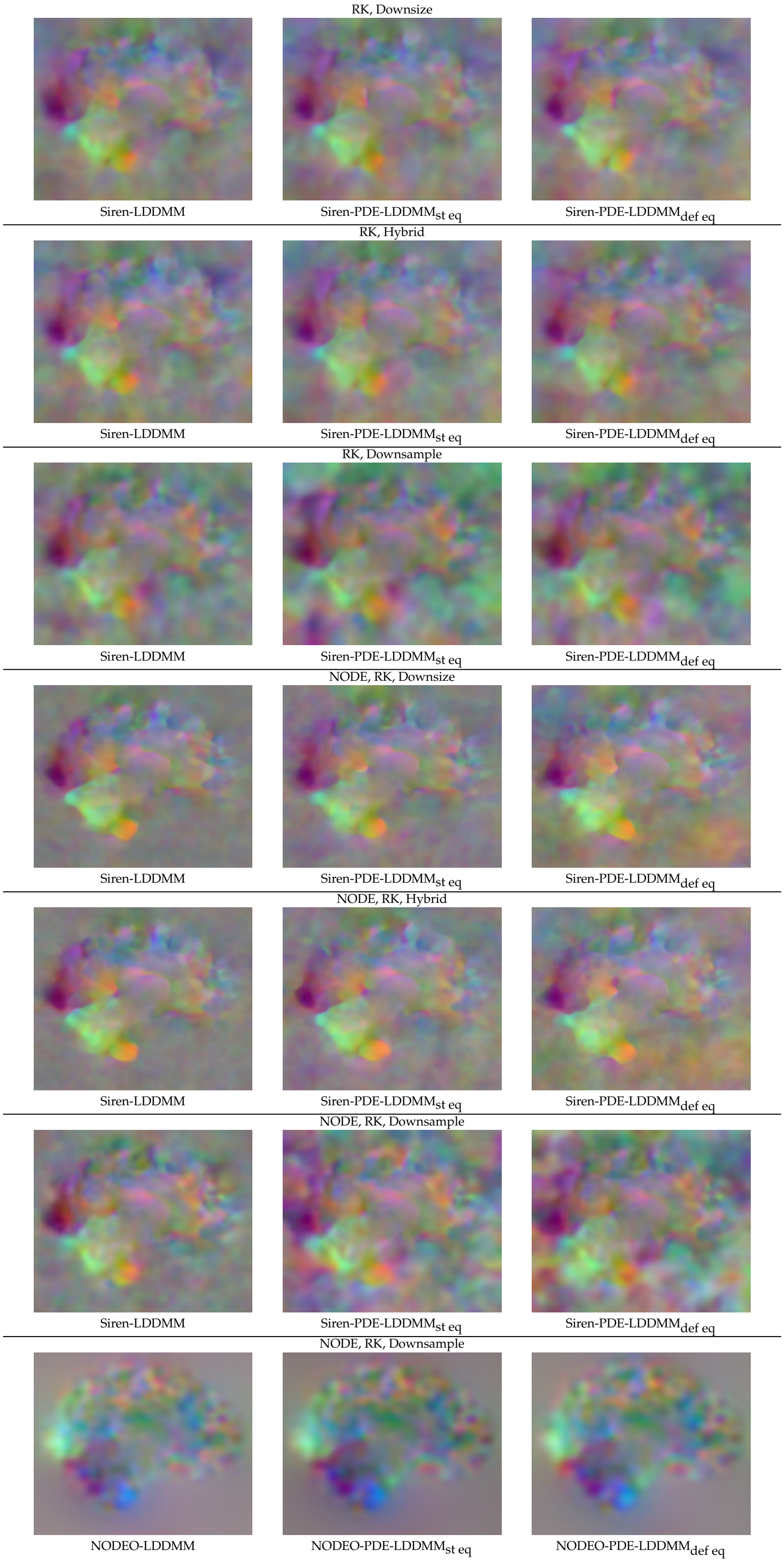


**Figure S5.** NIREP. Sagittal view of the displacement fields in a representative experiment. Siren and NODEO methods with RK and NODE-RK solvers. The RGB color map proposed in VoxelMorph paper is used for the color representation of the vector fields.

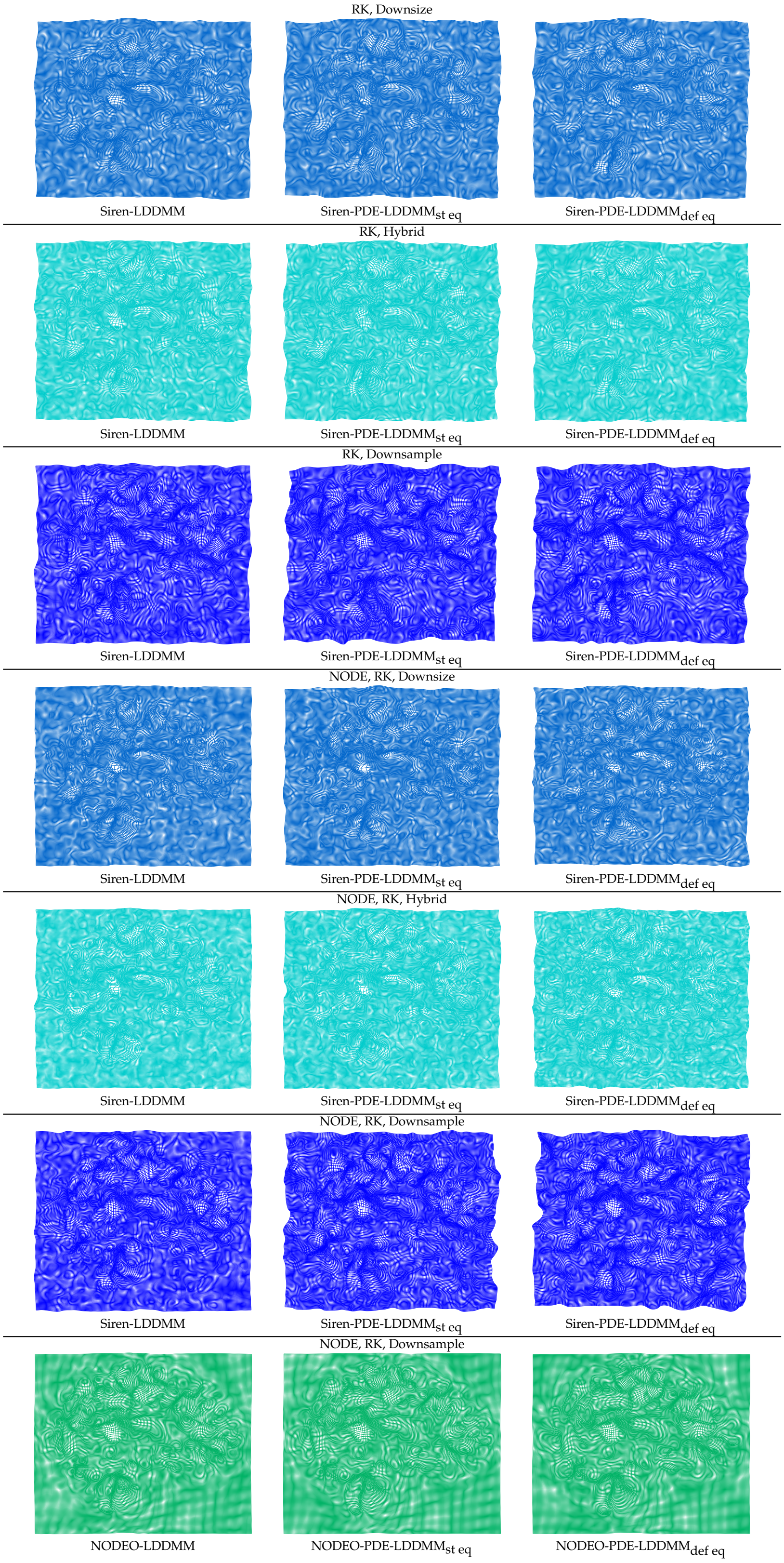


**Figure S6.** NIREP. Sagittal view of the transformation grids in a representative experiment. Siren and NODEO methods with RK and NODE-RK solvers.

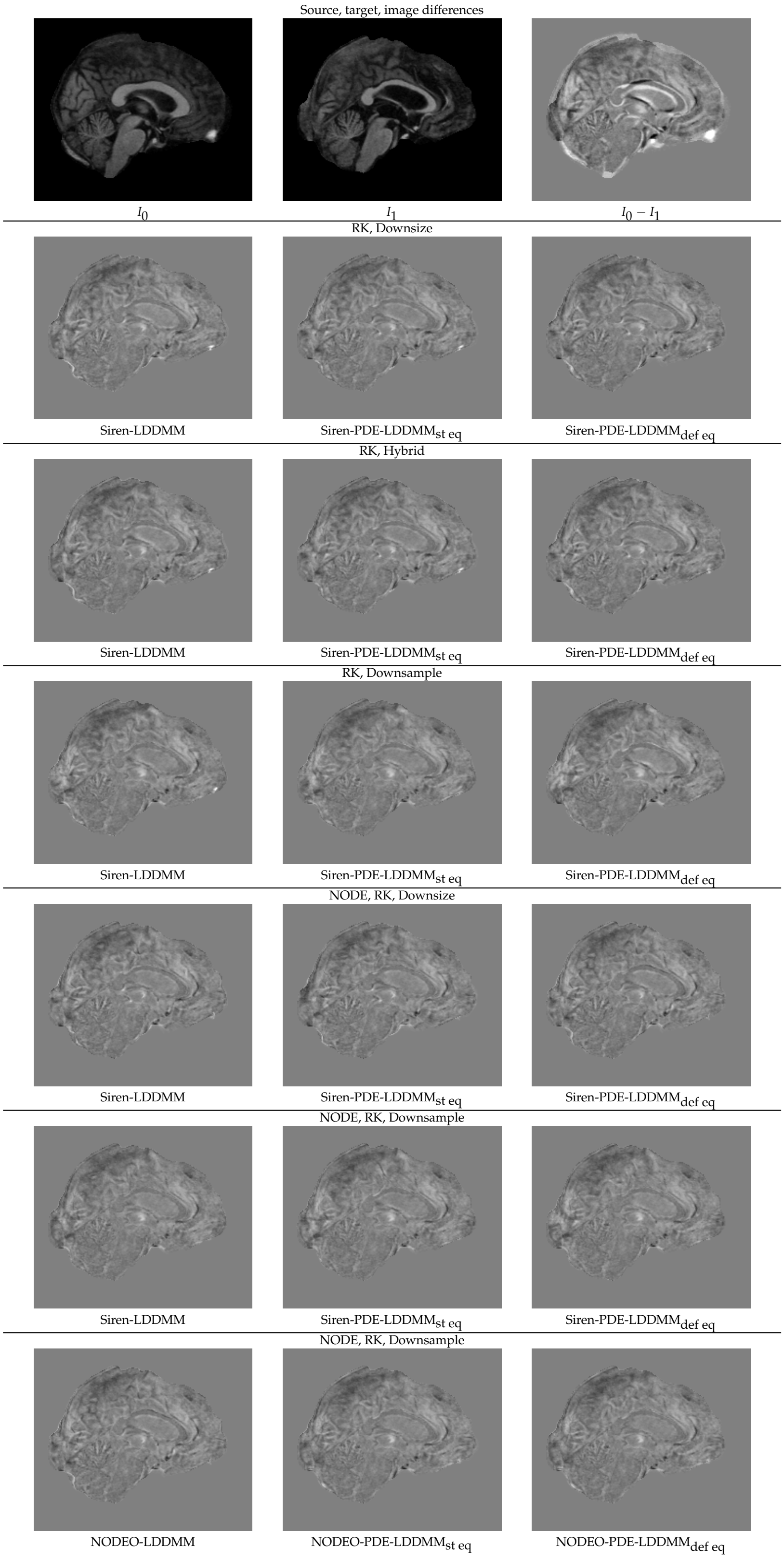


**Figure S7.** OASIS. Sagittal view of the differences after registration $I_0 \circ \varphi^{-1} - I_1$ in a representative experiment. Siren and NODEO methods with RK and NODE-RK solvers.

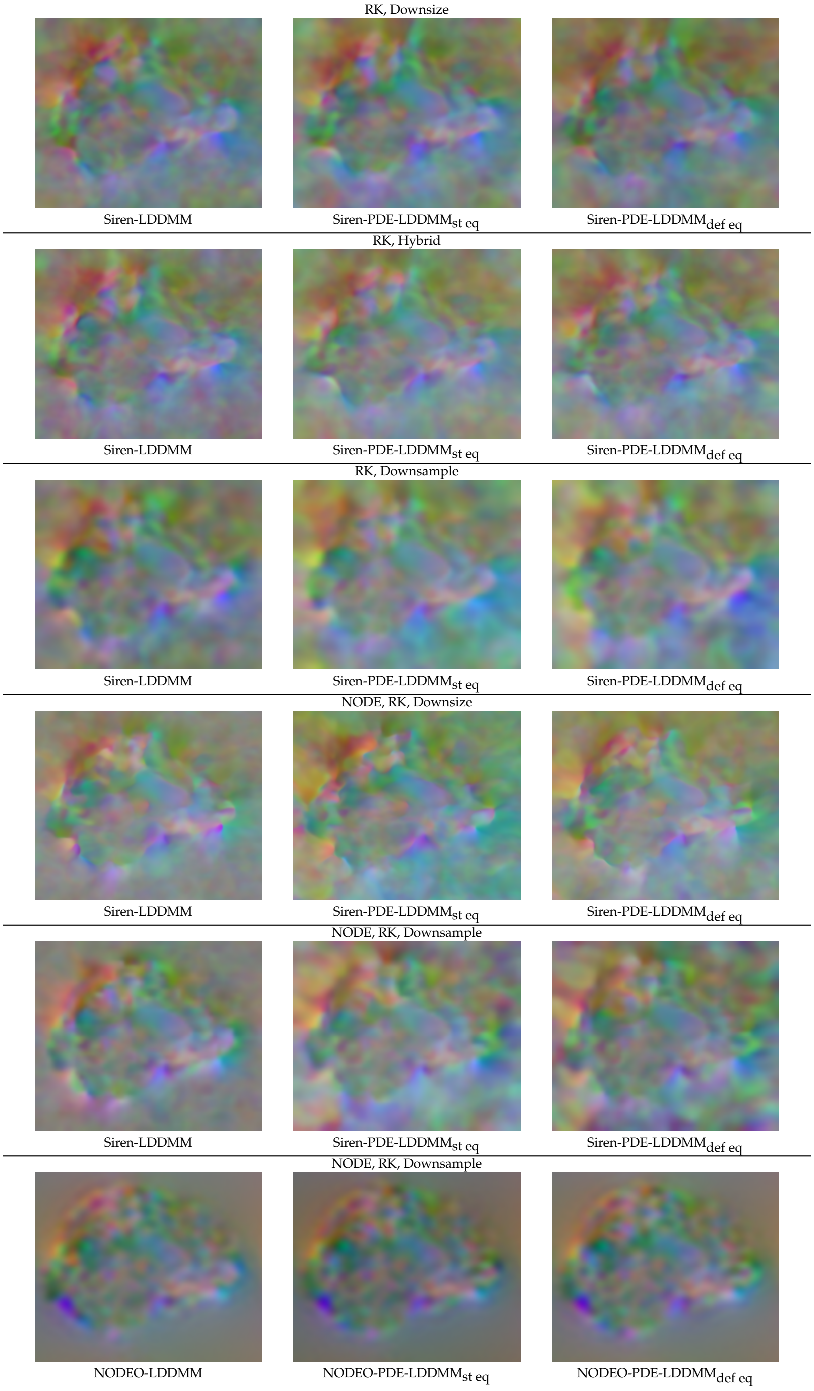


**Figure S8.** OASIS. Sagittal view of the displacement fields in a representative experiment. Siren and NODEO methods with RK and NODE-RK solvers. The RGB color map proposed in VoxelMorph paper is used for the color representation of the vector fields.

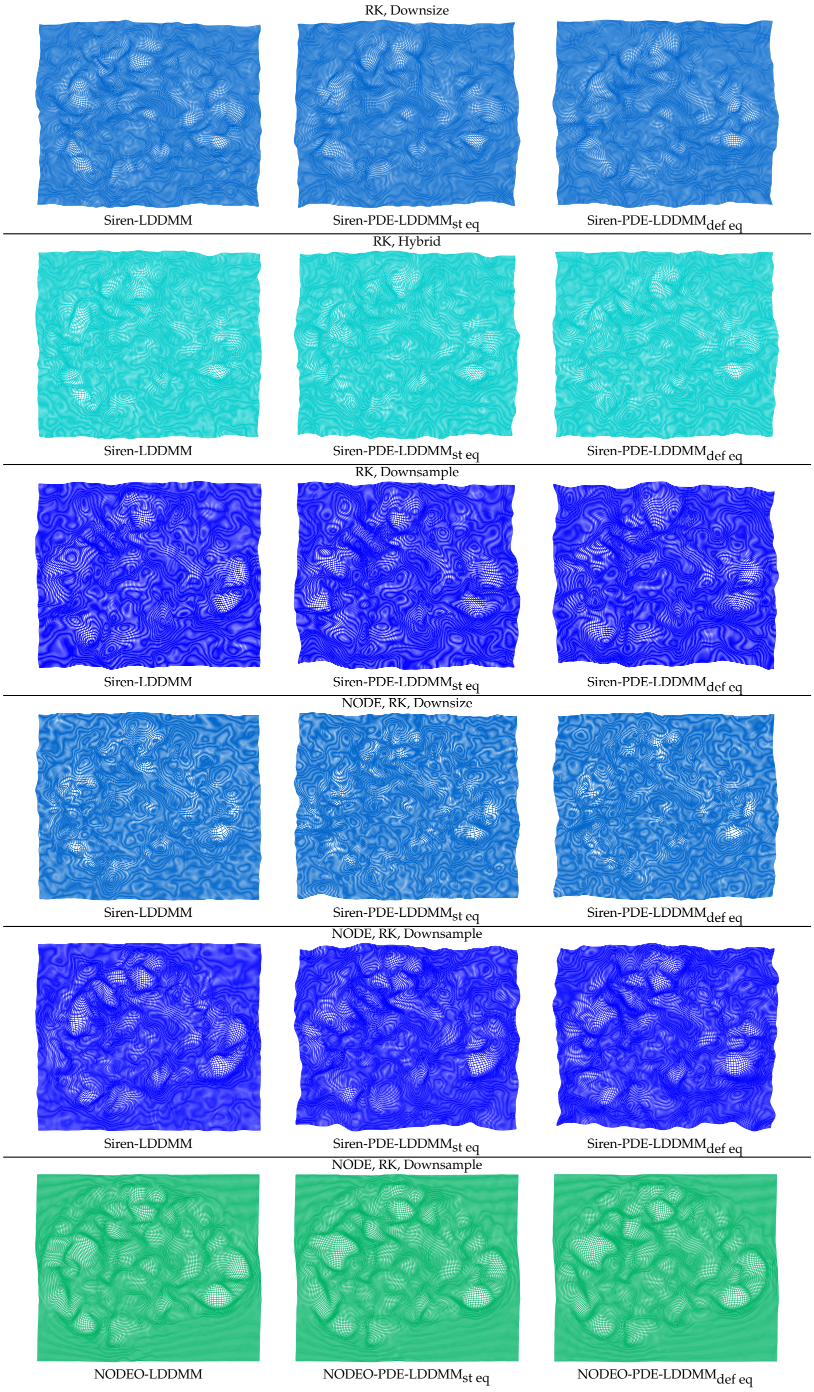


**Figure S9.** OASIS. Sagittal view of the transformation grids in a representative experiment. Siren and NODEO methods with RK and NODE-RK solvers.
